# An LRRK2 variant blocks NCOA4 trafficking upon iron overload, leading to ferroptotic death

**DOI:** 10.1242/jcs.264977

**Published:** 2026-07-27

**Authors:** Andres Goldman, Mai Nguyen, Joël Lanoix, Chongyang Li, Ahmed Fahmy, Yong Zhong Xu, Erwin Schurr, Pierre Thibault, Michel Desjardins, Heidi M. McBride

**Affiliations:** ^1^ Montreal Neurological Institute, McGill University, 3801 University Ave, Montreal, Quebec H3A 2B4, Canada; ^2^ Aligning Science Across Parkinson's (ASAP) Collaborative Research Network, Chevy Chase, MD 20815, USA; ^3^ Institute for Research in Immunology and Cancer (IRIC), Université de Montréal, Montreal, Quebec H3T 1J7, Canada; ^4^ Department of Chemistry, Université de Montréal, Montreal, Quebec H3T 1J7, Canada; ^5^ Département de pathologie et biologie cellulaire, Université de Montréal, Montreal, Quebec H3T 1J7, Canada; ^6^ Program in Infectious Diseases and Immunity in Global Health, The Research Institute of the McGill University Health Centre, Montreal, Quebec H4A 3J1, Canada; ^7^ Departments of Human Genetics and Medicine, Faculty of Medicine and Health Science, McGill University, Montreal, Quebec H3A 0G4, Canada

**Keywords:** LRRK2, Parkinson's disease, Iron, Ferritinophagy, Ferritin, NCOA4

## Abstract

Altered iron homeostasis has long been implicated in Parkinson's disease (PD), although the mechanisms have not been clear. Given the critical role of PD-related activating variants in leucine-rich repeat protein kinase 2 (LRRK2) within membrane trafficking pathways, we examined the impact of the homozygous variant LRRK2_G2019S_ on iron homeostasis within the RAW264.7 mouse macrophage cell line with high iron capacity. Proteomics analysis revealed a dysregulation of iron-related proteins in steady state, with highly elevated levels of ferritin light chain (FTL1) and a reduction of ferritin heavy chain (FTH1). LRRK2_G2019S_ mutant cells showed efficient ferritinophagy upon iron chelation, but upon iron overload, there was a near-complete block in the degradation of the ferritinophagy adaptor NCOA4. These conditions led to an accumulation of phosphorylated Rab8 (RAB8A) at the plasma membrane, which is selectively inhibited by LRRK type II kinase inhibitors. Iron overload then led to increased oxidative stress and ferroptotic cell death. These data implicate LRRK2 as a key regulator of iron homeostasis and point to the need for an increased focus on the mechanisms of iron dysregulation in PD.

## Teaser

**Highlighted Article:** The Parkinson's disease-related LRRK2_G2019S_ variant affects the ability of macrophages to handle iron overload due to alterations in NCOA4 and ferritin turnover, implicating a role for LRRK2 in the regulation of iron homeostasis.

## INTRODUCTION

Age-related neurodegenerative diseases are an increasing burden on the global population. Detailing the precise mechanisms responsible pathology in these complex diseases has been challenging. Hallmark features of Parkinson's disease (PD) are motor symptoms, namely tremor and rigidity, and selective death of neuromelanin-containing dopamine neurons in the *substantia nigra pars compacta* (SNpc) and *locus coeruleus*. About 5–15% of PD is familial and current estimates recognize over 50 causal variants and 100 risk alleles (Lim et al., 2024). These highlight contributions of mitochondria and lysosomal function, the spreading of prion-like synuclein filaments, innate and adaptive immune pathways, and more (Cannon and Gruenheid, 2022; Johnson et al., 2019; Tansey et al., 2022). In the remaining ∼90% of sporadic PD, it is unclear what the underlying causes might be, although environmental factors, such as heavy metals or pesticides (Aravindan et al., 2024), and various types of infection (Cannon and Gruenheid, 2022) are under intense investigation.

An early candidate cause of selective vulnerability of dopamine neurons in PD was their high dependence on iron metabolism required for synthesis of dopamine and production of neuromelanin, which captures and stores excess iron (Jenner, 1989; Sulzer et al., 2000). Patients with PD show dramatic losses of pigmented neuromelanin, which is a hallmark feature in autopsied brain. The loss of neuromelanin results in higher levels of free iron, which is being explored as a biomarker in patients with early-stage PD by high-resolution magnetic resonance imaging (MRI) quantitative susceptibility mapping (Guan et al., 2024). The mechanisms by which iron might be mishandled in the SNpc are understudied and causality to PD pathology has not been clearly established. However, there is increasing speculation that, in PD, increased iron toxicity causes ferroptotic cell death, making iron the Achilles' heel of dopaminergic neurons (Jenner, 1989; Masaldan et al., 2019; Riederer et al., 2023). To test these ideas clinically, a series of trials was initiated employing iron chelators in patients with PD, with mixed results (Devos et al., 2025). New formulations and approaches are still under investigation, highlighting the importance of a better understanding of the impact of iron on PD pathology.

A common risk factor for inherited PD are variants in the *LRRK2* gene, which encodes a large kinase with a complex array of domains including armadillo repeats, ankyrin, WD40, Ras of complex proteins (ROC) and C-terminal of ROC (COR) (ROC-COR) GTPase domains, leucine-rich repeats and a kinase domain (Alessi and Pfeffer, 2024). The penetrance of PD in carriers of LRRK2 pathogenic variants is typically ∼50% at age 70, rising to 75% at 80 years, implicating additional genetic and/or environmental factors in disease progression (Mata et al., 2023). LRRK2 variants have been the subject of intense research efforts, which have demonstrated an involvement in cellular functions that range from subcellular protein trafficking (Singh and Muqit, 2020), lysosomal repair and function (Bentley-DeSousa et al., 2025), cytoskeletal dynamics (Leschziner and Reck-Peterson, 2021), ciliogenesis and centrosome cohesion (Jaimon et al., 2025; Li et al., 2024; Lin et al., 2025; Madero-Perez et al., 2018), mitochondrial function (Buck and Sanders, 2025), synaptic transmission (Kuhlmann and Milnerwood, 2020), and immune responses (Peter and Strober, 2023). Despite much progress, the molecular basis of LRRK2 pathogenicity remains unclear.

All pathogenic LRRK2 variants, within and outside its kinase domain, increase its serine/threonine kinase activity, including the most common pathogenic substitution, p.G2019S (LRRK2_G2019S_) (Mata et al., 2023). In addition to autophosphorylation, LRRK2 substrates include numerous Rab GTPases that regulate vesicular transport; these are hyperphosphorylated in cells from patients with LRRK2-linked PD and LRRK2 PD models (Alessi and Pfeffer, 2024; Steger et al., 2016). The downstream impact of each phosphorylated Rab on membrane transport and signaling pathways appears to be highly dependent on the cell type and context, and the field has not reached consensus on which of the many implicated LRRK2 variant phenotypes are the driver of disease. However, Rab hyperphosphorylation is observed in other genetic forms of PD [e.g. VPS35-linked PD (Kadgien et al., 2021; Mir et al., 2018)] and idiopathic PD brains (Di Maio et al., 2018). Thus, there is a breadth of evidence associating the gain of function of LRRK2 kinase with PD pathogenesis.

In the context of iron handling, previous work reported context-specific changes in iron homeostasis in cells carrying the LRRK2_G2019S_ variant in HEK293T cells and induced pluripotent stem cell (iPSC)-derived microglia (Mamais et al., 2021, 2025 preprint). It was initially shown that the phosphorylation of Rab8 (RAB8A), a key LRRK2 substrate, reduced its interaction with its GTP exchange factor Rabin8 (also known as RAB3IP), which in turn led to the lysosomal degradation of the transferrin receptor. The authors observed the accumulation of ferritin and free iron in microglia of LRRK2_G2019S_ mice following injections with lipopolysaccharide, although the mechanisms underlying these changes *in vivo* were not established (Mamais et al., 2021). Although iron phenotypes have not been observed consistently within LRRK2_G2019S_ mutant cells, single-cell RNA sequencing experiments did report a cell-specific loss in ferritin heavy chain (*Fth1*) mRNA within LRRK2_G2019S_ mice, specifically in astrocytes, microglia and oligodendrocytes (Khan et al., 2024).

Given the evidence supporting a contributing role of dysregulated iron homeostasis in PD, we explored the impact of the LRRK2_G2019S_ variant on iron transport and handling in a cultured macrophage model. The data provide evidence that mouse RAW264.7 macrophages carrying the LRRK2_G2019S_ variant have altered iron homeostasis in steady-state conditions, which lead to an inability to handle iron overload, resulting in oxidative stress and ferroptotic cell death. We observed a block in the normal iron-induced expansion of lysosomes and in the microautophagy-driven degradation of the ferritin autophagy receptor NCOA4. Interestingly, iron overload conditions in LRRK2_G2019S_ cells led to high levels of phosphorylated Rab8 accumulating on the plasma membrane. This phosphorylation was resistant to the type I LRRK2 kinase inhibitor MLi-2 but remained sensitive to both the pan-specific type II kinase inhibitor rebastinib and the newly developed LRRK-selective type II kinase inhibitors RN277 and RN341 (Raig et al., 2025). Iron overload conditions are not common in healthy physiology but are a significant factor in PD brains. Should this aspect of LRRK2 variant function be relevant to the death of dopaminergic neurons, our results would suggest that the use of MLi-2 might not be effective in this context, where LRRK2-selective type II kinase inhibitors would be active in iron-related pathologies linked to LRRK2.

## RESULTS

### LRRK2_G2019S_ RAW264.7 macrophages have altered iron homeostasis

**Fig. 1.**
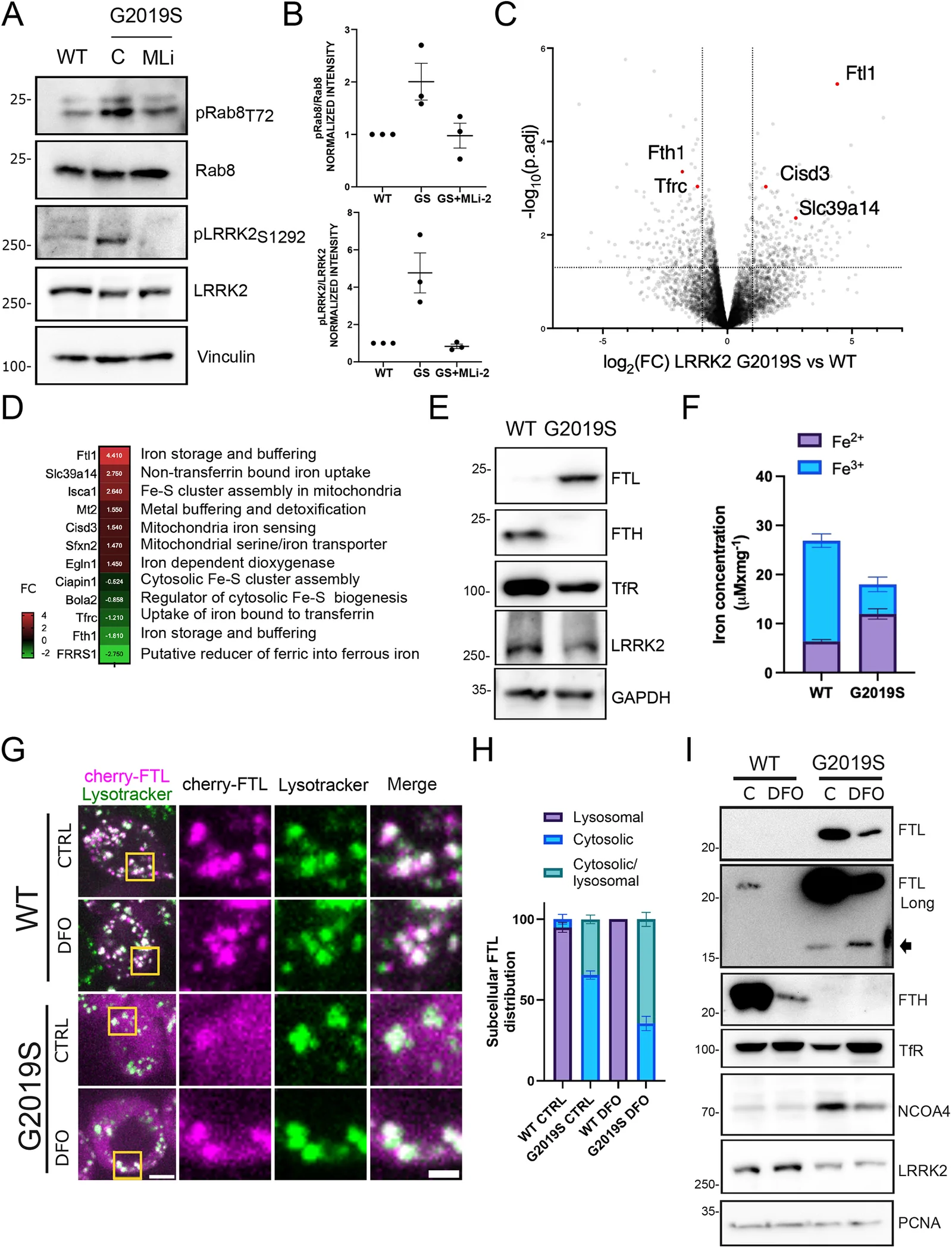
**LRRK2_G2019S_ RAW264.7 macrophages show changes in iron homeostasis.** (A) Western blot analysis on whole-cell lysates of pRab8_T72_ and pLRRK2_S1292_ in wild-type (WT) macrophages, LRRK2_G2019S_ macrophages (control, ‘C’) and LRRK2_G2019S_ macrophages treated with 1 μM MLi-2 for 16 h. Vinculin serves as the loading control. (B) Densitometry analysis of pRab8:Rab8 and pLRRK2:LRRK2 ratios from WT or LRRK2_G2019S_ (‘GS’) macrophages, with and without 1 μM MLi-2 treatment. The mean±s.d. of normalized band intensities from three biological replicates are shown. (C) Volcano plot showing differentially expressed proteins in LRRK2_G2019S_ compared to WT macrophages. On the *x*-axis, fold change (FC), and, on the *y*-axis, −log_10_[adjusted statistical significance (*P*_adj_)] are plotted. Dashed lines represent the cut-offs for analysis; proteins with |log_2_FC|>1 and *P*_adj_>1.3 (*P*<0.05) were considered as differentially expressed. Red dots depict some of the differentially expressed proteins important for iron metabolism. (D) Heat map and table depicting the fold change and the role of the main iron-related proteins differentially expressed in LRRK2_G2019S_ macrophages. Source data are found in Table S1. (E) Western blot analysis on whole-cell lysates of iron markers in WT and LRRK2_G2019S_ macrophages. GAPDH serves as the loading control. Blots represent one of three independent experiments. (F) Levels of ferric (Fe^3+^) and ferrous (Fe^2+^) iron in WT and LRRK2_G2019S_ macrophages. Iron is quantified as micromoles of iron per mg of protein. Prior to iron quantification, cells were treated for 16 h with 100 μM ferrous ammonium sulfate (FAS). The mean±s.d. of three biological replicates are shown. (G) Representative images of mCherry–FTL distribution in WT and LRRK2_G2019S_ macrophages, treated without (control, CTRL) or with 250 μM deferoxamine (DFO) for 24 h. Lysosomes were stained with 100 nM LysoTracker (green). Scale bars: 4 μm (left); 1 μm (magnified views). (H) Qualitative classification of mCherry–FTL distribution shown in G. Cells analyzed per biological replicate: WT control, *n*=80, 83, 74; LRRK2_G2019S_ control, *n*=109, 94, 97; WT DFO, *n*=69, 63, 56; LRRK2_G2019S_ DFO, *n*=84, 78, 79. The mean±s.d. of three separate biological replicates are shown. (I) Western blot analysis of NCOA4, FTL (short and long exposures), FTH and TfR levels on whole-cell lysates of WT and LRRK2_G2019S_ macrophages, treated without (control, ‘C’) or with 250 μM DFO for 24 h. PCNA serves as the loading control. The black arrow indicates the proteolytic cleavage product of FTL. Blots represent one of three independent experiments.

Macrophages ingest aged red blood cells and have a unique capacity to handle high levels of iron (Winn et al., 2020). Therefore, we considered macrophages, given the high levels of LRRK2 expression (Gardet et al., 2010), as a good model system to interrogate the impact of pathogenic homozygous LRRK2 variants on iron handling. To this end, we generated a CRISPR knock-in RAW264.7 macrophage cell line carrying the LRRK2_G2019S_ mutation (Fig. S1A). To confirm the increase in LRRK2 kinase activity in this line, we probed cell extracts with two phospho-specific antibodies (Fig. 1A). LRRK2 autophosphorylation was increased, as seen with an antibody recognizing LRRK phosphorylation at S1292 (pLRRK2_S1292_). A second antibody recognizing phosphorylated Rab8_T72_ (with lower specificity for pRab10 and pRab35) (Lis et al., 2018) further confirmed increased kinase activity in the CRISPR-edited LRRK2_G2019S_ macrophages, with no change in total Rab8 levels across the genotypes (Fig. 1A). Both phosphorylation events were reversed upon treatment with the type I LRRK2 kinase inhibitor MLi-2 (Fig. 1A, quantification in Fig. 1B). These data confirm the successful generation of the gain-of-function variant. We then performed unbiased whole-cell proteomics and found significant changes in many proteins important for iron homeostasis (Fig. 1C,D; Table S1). Protein quantification by western blotting validated downregulation of the transferrin receptor (TfR, also known as TFRC) responsible for iron uptake, and downregulation of ferritin heavy chain (FTH, also known as FTH1) and upregulation of ferritin light chain (FTL, also known as FTL1), both critical for iron storage (Fig. 1E; quantified in Fig. S1B). Iron is found inside the cell in its reactive ferrous (Fe^2+^) or mineralized ferric (Fe^3+^) state. Fe^2+^ is the form required by the cell to forge iron sulfur clusters and heme groups, whereas excess free ferrous iron can catalyze the generation of free radical species through the Fenton reaction (Galy et al., 2024). This makes Fe^2+^ essential, yet potentially toxic, for the cell. Excess Fe^2+^ is rapidly captured by cytosolic ferritin, where iron is harmlessly stored in the mineralized ferric Fe^3+^ state (Chasteen and Harrison, 1999; Melman and Bou-Abdallah, 2020). FTH and FTL together form cage-like complexes that can hold up to ∼4500 atoms of Fe^3+^. FTH has enzymatic ferroxidase activity required to oxidize Fe^2+^ into Fe^3+^ for safe storage (Melman and Bou-Abdallah, 2020). We quantified the iron content and composition and found that LRRK2_G2019S_ macrophages have reduced total iron content, marked by a selective loss of mineralized iron Fe^3+^, and a 2-fold increase in Fe^2+^ content compared to those in the wild-type (WT) control (Fig. 1F). This result was validated using the fluorescent FerroOrange probe, which reported a similar 2-fold increase of labile Fe^2+^ in LRRK2_G2019S_ macrophages (Niwa et al., 2014) (Fig. S1C,D).

Iron quantification revealed a cellular reduction in total iron, yet we observed excess FTL and reduced TfR, which canonically suggests a translational response to iron overload through the iron-response elements (IREs) within their mRNAs (Galy et al., 2024; Rouault, 2006). We therefore tested a series of additional iron-related proteins regulated by either the iron–sulfur cluster-binding protein aconitase (ACO1, also known as iron regulatory protein 1 or IRP1) or the Fe^2+^-regulated iron regulatory protein 2 (IRP2, also known as IREB2). LRRK2_G2019S_ expression did not elicit any changes in the levels of the HERC2–FBXL5–IRP2 axis (Fig. S1E), indicating that IRP2 is not activated. In addition, we found that the levels of other proteins harboring IREs such as DMT1 (also known as SLC11A2) or ferroportin (SLC40A1) were unaffected in LRRK2_G2019S_ macrophages (Fig. S1E), implying that the IRP1–IRP2 axis is not involved in the upregulation of FTL or downregulation of TfR. This indicates that the changes in FTL and FTH levels are not through canonical IRE mechanisms.

We further tested the enzymatic activity of the iron–sulfur cluster-containing protein aconitase, which is often used as a proxy to monitor any deficits in iron–sulfur cluster assemblies (Beinert and Kennedy, 1993). As expected, given the evidence that the translational regulation by aconitase was unaltered, LRRK2_G2019S_ macrophages did not show any change in aconitase activity (Fig. S1F). This further indicates that the delivery of ferrous Fe^2+^ iron to mitochondria for the biogenesis of iron–sulfur complexes remained intact. Therefore, LRRK2_G2019S_ leads to a significant reduction in total cellular iron (Fig. 1F), primarily due to the loss of stored ferric Fe^3+^ and an increase in ferrous Fe^2+^, without an impairment of functional delivery of Fe^2+^ to mitochondria or the modulation of iron-related protein synthesis pathways (Fig. S1E,F).

FTH is critical for the lysosomal degradation of ferritin complexes through an autophagic process termed ferritinophagy (Dowdle et al., 2014; Kuno et al., 2022; Mancias et al., 2015, 2014; Ohshima et al., 2022; Zhao et al., 2024). Given the absence of FTH expression, we considered that this could cause a block in complex turnover, resulting in the observed increases in FTL levels. To monitor the turnover of FTL, we generated WT and LRRK2_G2019S_ macrophages stably expressing N-terminal mCherry-tagged FTL to visualize its cellular distribution (Ohshima et al., 2022). In untreated WT cells, the mCherry–FTL signal was primarily distributed within LysoTracker Green-positive lysosomes, with some cytosolic signal (Fig. 1G, quantified in Fig. 1H). In contrast, LRRK2_G2019S_ macrophages showed a highly diffuse, cytosolic mCherry–FTL signal and faint lysosomal localization (Fig. 1G, third row). This observation is consistent with impaired or inefficient lysosomal targeting of FTL in steady-state conditions.

To determine whether LRRK2_G2019S_ macrophages were capable of induced ferritin degradation, we activated ferritinophagy using the iron chelator deferoxamine (DFO) to remove iron from the cytoplasm (Fig. 1G–I). Ferritinophagy is regulated by the action of nuclear receptor coactivator 4 (NCOA4), the adaptor protein that binds and delivers ferritin cages into the autophagosome (Dowdle et al., 2014; Kuno et al., 2022; Mancias et al., 2014; Ohshima et al., 2022; Zhao et al., 2024). NCOA4 is an iron and iron sulfur cluster-binding protein (Kuno et al., 2022; Zhao et al., 2024). Therefore, iron levels modulate NCOA4–ferritin interactions, allowing the fine tuning of ferritin turnover, in which NCOA4 bound to ferritin promotes the formation of liquid–liquid phase-separated condensates that are selectively degraded through autophagy (Ohshima et al., 2022). Looking first at FTL, DFO treatment elicited an increase in the appearance of FTL in lysosomes in WT and LRRK2_G2019S_ macrophages, consistent with functional ferritinophagy (Fig. 1G, second and fourth rows, quantification in Fig. 1H). Using western blotting, we confirmed that iron chelation led to increased clearance of FTL in WT and LRRK2_G2019S_ macrophages (Fig. 1I). With longer exposure, the degradative products of FTL were visible in DFO-treated LRRK2_G2019S_ macrophages (Fig. 1I, black arrow). The ferritinophagy adaptor NCOA4 was also highly elevated in LRRK2 mutant cells at steady state (Fig. 1I), and we observed that iron chelation led to clearance of NCOA4 in LRRK2_G2019S_ macrophages, consistent with functional ferritinophagy.

### LRRK2_G2019S_ cells show defects in NCOA4 turnover upon iron overload

The inverse of iron chelation is the response to iron overload, which requires NCOA4 to segregate away from ferritin cages, resulting in a diffuse cytosolic distribution of ferritin that stably mineralizes and stores excess iron as Fe^3+^ (Galy et al., 2024). In high-iron conditions, NCOA4 binds iron–sulfur clusters, which promotes binding of the ubiquitin E3 ligase HERC2 to drive NCOA4 degradation through the proteasome (Zhao et al., 2024). In a second degradation pathway, ferric Fe^3+^-bound NCOA4 can drive assembly into phase-condensed foci that become internalized into lysosomes through a microautophagy pathway (Kuno et al., 2022). At later time points of iron overload, NCOA4 can also carry low levels of ferritin into the lysosomes through microautophagy to maintain pools of labile iron (Kuno et al., 2022). The precise regulation of these distinct mechanisms remains under intense investigation. In our experiments with ferrous ammonium sulfate (FAS) to induce iron overload, we observe the expected diffuse cytosolic staining of FTL in WT cells (Fig. S2A,B). In LRRK2_G2019S_ macrophages, FTL was already diffuse in untreated cells, which remained the case upon iron overload, with the addition of a few bright foci resembling phase-condensed foci (Fig. S2A,B).

**Fig. 2.**
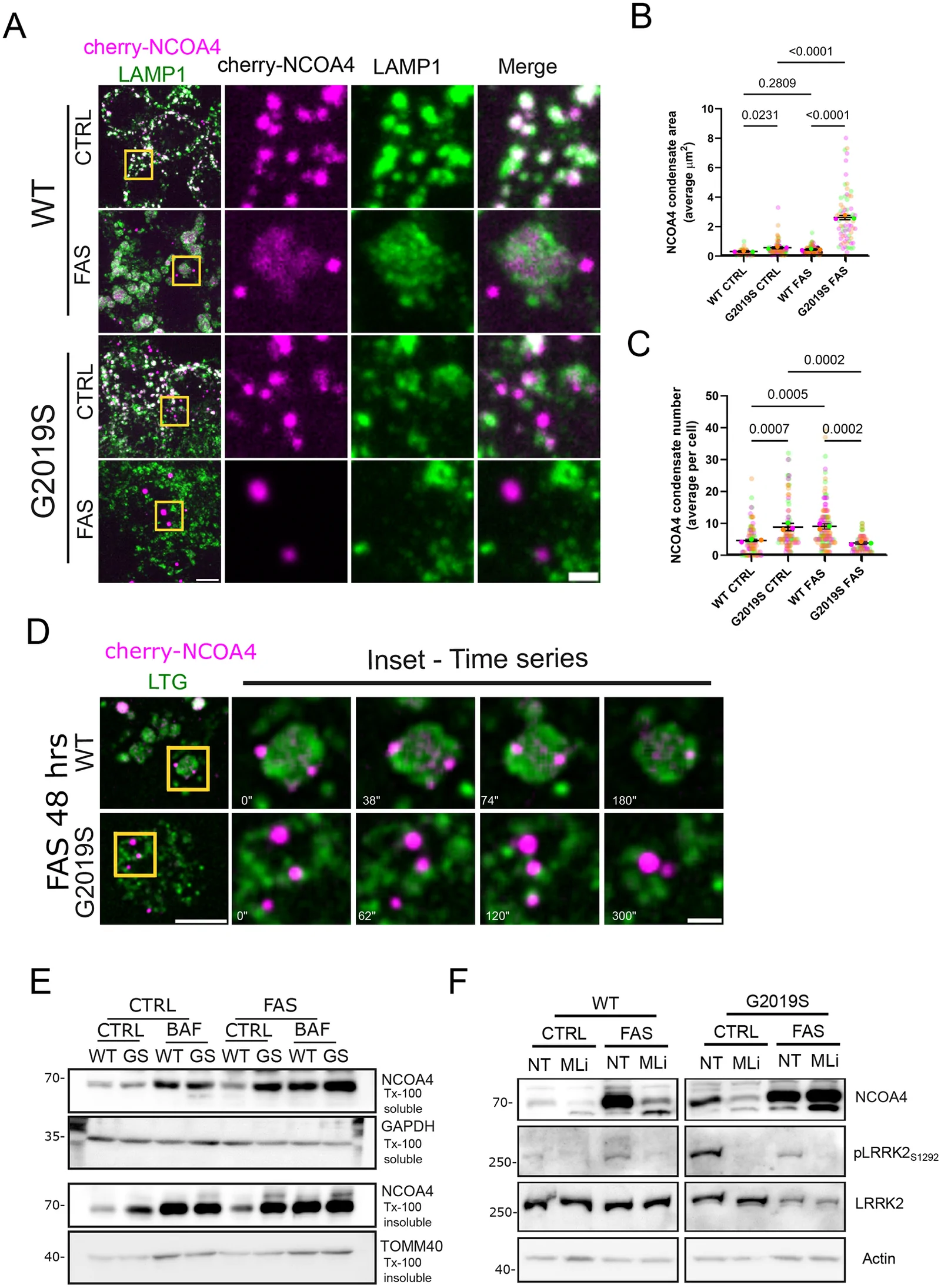
**LRRK2_G2019S_ macrophages show defects in NCOA4 turnover upon iron overload.** (A) Representative images of mCherry–NCOA4 distribution in WT and LRRK2_G2019S_ macrophages, treated without (control, CTRL) or with 100 μM FAS for 16 h. Lysosomes were stained with an anti-LAMP1 antibody (green). Scale bars: 4 μm (left); 1 μm (magnified views). (B) Quantification of the average size of cytoplasmic mCherry–NCOA4 condensates calculated as μm^2^ per cell of data shown in A. Cells analyzed per biological replicate: WT control, *n*=26, 20, 26; LRRK2_G2019S_ control, *n*=36, 33, 32; WT FAS, *n*=40, 44, 43; LRRK2_G2019S_ FAS, *n*=22, 27, 23. The mean±s.d., single values of three biological replicates and *P*-values calculated using one-way ANOVA with Tukey's post hoc test for multiple comparisons are shown. (C) Quantification of the average number of cytoplasmic mCherry–NCOA4 condensates per cell of data shown in A. For each condition, the number of analyzed cells is the same as that in B. The mean±s.d. and single values of three separate biological replicates and *P*-values calculated using one-way ANOVA with Tukey's post hoc test for multiple comparisons are shown. (D) Representative time-lapse sequences of mCherry–NCOA4 condensates in WT and LRRK2_G2019S_ macrophages treated for 48 h with 100 μM FAS. Lysosomes were stained with LysoTracker Green (LTG). Time-lapse experiments were acquired for 5 min, capturing both channels every 2 s. Scale bars: 4 μm (left); 1 μm (insets). The video sequence represents events observed consistently across three biological replicates from cells analyzed in B. (E) Western blot analysis of Triton X-100-soluble and -insoluble NCOA4 levels in WT and LRRK2_G2019S_ RAW264.7 macrophages first treated without or with 100 μM FAS for 16 h, and then with DMSO or 25 nM bafilomycin (BAF) for 3 h. GAPDH and TOMM40 serve as the loading controls. Blots represent one of three independent experiments. (F) Western blot analysis of NCOA4 on whole-cell extracts of WT and LRRK2_G2019S_ macrophages left untreated (CTRL) or treated with 100 μM FAS for 16 h, and then not treated (NT) or treated with 1 μM MLi-2 for 8 h in the absence of iron. Phosphorylation levels of LRRK2 on S1292 serve as the positive control, actin serves as the loading control. Blots represent one of three independent experiments.

A critical distinction emerged between the genotypes when imaging mCherry–NCOA4. In WT untreated cells, mCherry–NCOA4 was seen primarily within lysosomes (Fig. 2A), but in LRRK2_G2019S_ macrophages, mCherry–NCOA4 had limited lysosomal colocalization and appeared in bright cytosolic foci (Fig. 2A, number and size of foci quantified in Fig. 2B,C). In WT cells treated with FAS, NCOA4 appeared in bright cytosolic foci and within lysosomes, which became enlarged (seen upon FAS treatment in Fig. 2A and Fig. S2C, quantified in Fig. S2D,E). In contrast, LRRK2_G2019S_ macrophages showed NCOA4 accumulating in larger bright cytosolic (non-lysosomal) foci, suggesting very little internalization into lysosomes in high-iron conditions. Quantification of foci number and size showed that LRRK2_G2019S_ macrophages had fewer NCOA4 foci with a significant increase in their size relative to those in WT cells (Fig. 2B,C).

Time-lapse imaging of NCOA4 foci revealed their entry into enlarged lysosomes in FAS-treated WT cells (Fig. 2D, Movie 1), as previously described (Ohshima et al., 2022). Conversely, in LRRK2_G2019S_ cells, NCOA4 foci did not associate with lysosomes and were thus seemingly unable to undergo microautophagy (Fig. 2D, Movie 2). To biochemically confirm that the bright cytosolic foci reflected the formation of phase-separated condensates, we examined the amount of NCOA4 within Triton X-100-insoluble fractionations (Ohshima et al., 2022). Consistently, total insoluble NCOA4 levels were increased in LRRK2_G2019S_ macrophages (Fig. 2E). Addition of bafilomycin to block degradation in lysosomes led to accumulation of NCOA4 in WT cells in both untreated and iron-loaded (FAS-treated) conditions (Fig. 2E). NCOA4 also accumulated in bafilomycin-exposed LRRK2_G2019S_ cells; however, upon bafilomycin addition, the FAS-induced iron overload failed to increase NCOA4 levels further, indicating a block in lysosomal flux (Fig. 2E).

To examine the expected LRRK2 kinase dependency of the NCOA4 phenotype, we used the LRRK2 inhibitor MLi-2 (Fell et al., 2015). MLi-2 reversed the accumulation of NCOA4 in steady-state conditions in both genotypes, indicating that NCOA4 levels are coupled to LRRK2 kinase activity (Fig. 2F). Upon iron overload in WT cells, we observed an accumulation of NCOA4, which was again reversed upon MLi-2 treatment. Surprisingly, the increased levels of NCOA4 seen in LRRK2_G2019S_ cells were resistant to LRRK2 kinase inhibition, even though LRRK2 autophosphorylation was efficiently lost, demonstrating target engagement (Fig. 2F). Moreover, MLi-2 treatment did not rescue the alterations in ferritin proteins in untreated cells, nor did it rescue the lysosomal morphology linked to iron overload (Fig. S2F–H). These findings indicate that the block in NCOA4 degradation induced by iron overload within LRRK2_G2019S_ cells is resistant to MLi-2 treatment.

### The impact of LRRK_G2019S_ on iron metabolism reveals differential sensitivities to type I and type II kinase inhibitors

**Fig. 3.**
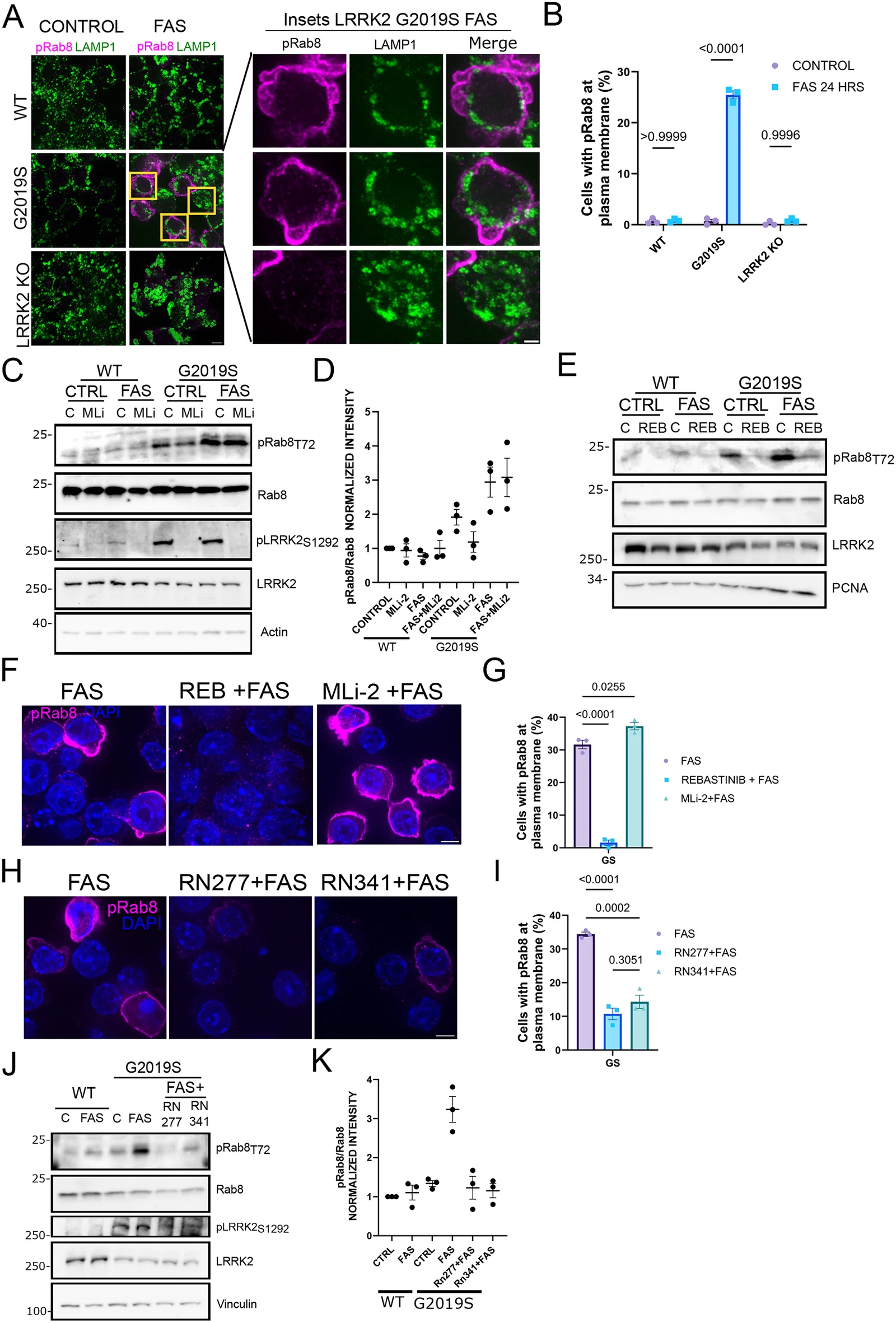
**The impact of LRRK2_G2019S_ on iron metabolism reveals differential sensitivity to type I and type II kinase inhibitors.** (A) Representative images of endogenous pRab8 distribution in WT, LRRK2_G2019S_ and LRRK2 knockout (KO) macrophages treated without (control) or with 250 μM FAS for 24 h. Lysosomes were stained with an anti-LAMP1 antibody. Scale bars: 10 μm; 5 μm (insets). (B) Quantification of the percentage of cells showing positive pRab8 signal at the plasma membrane. Cells analyzed per biological replicate: WT control, *n*=326, 241, 153; WT FAS, *n*=338, 69, 132; LRRK2_G2019S_ control, *n*=336, 160, 123; LRRK2_G2019S_ FAS, *n*=168, 247, 145; LRRK2 KO control, *n*=326, 330, 158; LRRK2 KO FAS, *n*=364, 100, 160. The mean±s.d. of three biological replicates and *P*-values calculated using one-way ANOVA with Tukey's post hoc test for multiple comparisons are shown. (C) Western blot analysis of pRab8 in WT and LRRK2_G2019S_ macrophages treated without (control, ‘C’) or with 1 μM MLi-2 for 2 h, and then left untreated (control, CTRL) or exposed to 250 μM FAS for 24 h in the presence of the inhibitor. Phosphorylation levels of LRRK2 on S1292 serve as the positive control and actin serves as the loading control. (D) Densitometry analysis of pRab8 changes shown in C. The mean±s.d. of normalized band intensities from three separate biological replicates are shown. (E) Western blot analysis of pRab8 in WT and LRRK2_G2019S_ macrophages treated without (control, ‘C’) or with 1 μM rebastinib (REB) for 2 h, and then left untreated (control, CTRL) or exposed to 250 μM FAS for 24 h in the presence of the inhibitor. PCNA serves as the loading control. Blots represent one of three independent experiments. (F) Representative images of endogenous pRab8 distribution in LRRK2_G2019S_ macrophages pre-treated for 2 h with 1 μM rebastinib or 1 μM MLi-2 and then treated for 24 h with 250 μM FAS in the presence of the inhibitors. DNA is stained with DAPI. Scale bar: 10 μm. (G) Quantification of the percentage of cells showing positive pRab8 signal at the plasma membrane from the experiment shown in F. Cells analyzed per biological replicate: LRRK2_G2019S_ FAS, *n*=148, 115, 73; LRRK2_G2019S_ rebastinib+FAS, *n*=150, 45, 76; LRRK2_G2019S_ MLi-2+FAS, *n*=120, 85, 107. The mean±s.d. of three biological replicates and *P*-values calculated using one-way ANOVA with Tukey's post hoc test for multiple comparisons are shown. (H) Representative images of endogenous pRab8 distribution in LRRK2_G2019S_ macrophages pre-treated for 2 h with 5 μM RN277 or 5 μM RN341 and treated for 24 h with 250 μM FAS in the presence of the inhibitors. DNA is stained with DAPI. Scale bar: 10 μm. (I) Quantification of the percentage of cells showing positive pRab8 signal at the plasma membrane from the experiment shown in H. Cells analyzed per biological replicate: LRRK2_G2019S_ FAS, *n*=147, 90, 38; LRRK2_G2019S_ RN277+FAS, *n*=95, 170, 101; LRRK2_G2019S_ RN341+FAS, *n*=89, 65, 82. The mean±s.d. of three biological replicates and *P*-values calculated using one-way ANOVA with Tukey's post hoc test for multiple comparisons are shown. (J) Western blot analysis of pRab8 in WT and LRRK2_G2019S_ macrophages treated without (control, ‘C’) or with 250 μM FAS for 24 h. LRRK2_G2019S_ macrophages were also treated without or with 5 μM RN277 or 5 μM RN341 for 2 h, and then left untreated or exposed to 250 μM FAS for 24 h in the presence of the inhibitors. Vinculin serves as the loading control. (K) Densitometry quantification of data shown in J. The mean±s.d. of normalized band intensities from three biological replicates are shown.

Given the unexpected resistance of the NCOA4 phenotypes to MLi-2 in LRRK2_G2019S_ cells upon iron overload conditions (Fig. 2F), we explored the cellular localization of phosphorylated Rab8 signal in these conditions. In both WT and LRRK2_G2019S_ cells, pRab8 was barely detectable with immunofluorescence at steady state (Fig. 3A). Iron overload induced by FAS did not change this pattern in WT or CRISPR-generated LRRK2 knockout (KO) cells. However, in ∼30% of LRRK2_G2019S_ cells, FAS induced a pronounced upregulation and accumulation of pRab8 signal on plasma membrane ruffles and coincided with cells showing significant blebbing (Fig. 3A, insets; quantification in Fig. 3B). Western blotting confirmed that the pRab8 signal was specific to LRRK2_G2019S_ cells and was enhanced upon iron overload (Fig. S3A,B). To confirm the specificity of the pRab8 immunofluorescence signal, we used an siRNA approach for validation. Silencing Rab8 led to a near-complete loss of the pRab8 plasma membrane signal, with a partial effect seen upon silencing Rab10 (Fig. S3C–E). We further observed a pRab8 signal consistent with the previously described recruitment of Rab8 to the centrioles in mitotic cells (Madero-Perez et al., 2018) (Fig. S3F). Lastly, a strong lysosomal pRab8 signal was seen in cells treated with the lysosomal destabilizing agent Leu-Leu methyl ester (LLOME) (Fig. S3G); under this condition, it has been established that LRRK2-mediated Rab8 phosphorylation leads to Rab8 recruitment to damaged lysosomes (Herbst et al., 2020; Mamais et al., 2021). These data confirm that the anti-pRab8 antibody shows specificity to Rab8 in immunofluorescence experiments, showing recruitment to the plasma membrane in iron-loaded LRRK2_G2019S_ cells.

A similar recruitment of Rab8 to plasma membrane ruffles was previously observed upon activation of Toll-like receptors (TLRs) (Luo et al., 2018; Tong et al., 2021; Wall et al., 2017). It was established that TLR-mediated activation of Rab8 recruits its effector PI3Kγ (also known as PIK3CG), to generate a signaling platform required to activate the AKT–mTOR pathway. We therefore examined AKT phosphorylation at S473 as a functional readout of pRab8 recruitment to the cell surface over a time course of FAS treatment. Treating cells for 24 h with increasing levels of FAS led to pronouced AKT phosphorylation in LRRK2_G2019S_ macrophages, with no effect on WT and LRRK2 KO macrophages (Fig. S3H, quantified in Fig. S3I). This supports the idea that plasma membrane-associated pRab8 is activating established Rab8 function.

We next tested whether the pRab8 signal seen upon iron overload in LRRK2_G2019S_ cells was sensitive to MLi-2. As with NCOA4, the pRab8 signal was also resistant to MLi-2 treatment in LRRK2_G2019S_ cells exposed to high iron, even when the autophosphorylation of LRRK2 was blocked by the drug (Fig. 3C,D). WT cells showed very little pRab8 upon iron loading, which also appeared to be insensitive to MLi2, while autophosphorylation of LRRK2 was inhibited (Fig. 3C). The insensitivity to MLi-2 was inconsistent with the overwhelming evidence that the phenotypes of the LRRK2 variant are due to hyperactivity of the kinase domain. MLi-2 is a type I kinase inhibitor, a class that binds to the active site and stabilizes the active-like or closed conformation of the kinase. Type II inhibitors, in contrast, stabilize the inactive, open conformation of the kinase. Recent cryo-electron microscopy structures of LRRK2 bound to type I and type II inhibitors have highlighted the conformations adopted by LRRK2 when bound to either type of inhibitor (Sanz Murillo et al., 2023; Tasegian et al., 2021; Zhu et al., 2024). We therefore considered whether iron overload might somehow impact the conformation of LRRK2 and tested a broad-spectrum type II kinase inhibitor called rebastinib, shown previously to efficiently block LRRK2 kinase activity (Tasegian et al., 2021). Unlike MLi-2, treatment of both WT and LRRK2_G2019S_ cells with rebastinib efficiently blocked the phosphorylation of Rab8 in both the presence or absence of iron overload, as seen by western blotting (Fig. 3E, quantified in Fig. S3J) and by immunofluorescence (Fig. 3F, quantified in Fig. 3G). As rebastinib has broad specificity, with effects on ∼30% of the kinome, we turned to the newly developed LRRK-selective type II kinase inhibitors RN277 and RN341 (Raig et al., 2025). As with rebastinib, pre-treatment with either of the two new drugs efficiently reversed the FAS-induced plasma membrane pRab8 signal, as seen by immunofluorescence (Fig. 3H, quantified in Fig. 3I) and western blotting (Fig. 3J,K). These data confirm the kinase dependence of Rab8 phosphorylation at the plasma membrane in LRRK2_G2019S_ cells upon treatment with FAS and highlights a cellular condition in which we observe differential efficacy between type I and type II LRRK2 kinase inhibitors.

### LRRK2_G2019S_ cells are hypersensitive to iron-induced oxidation and ferroptotic cell death

**Fig. 4.**
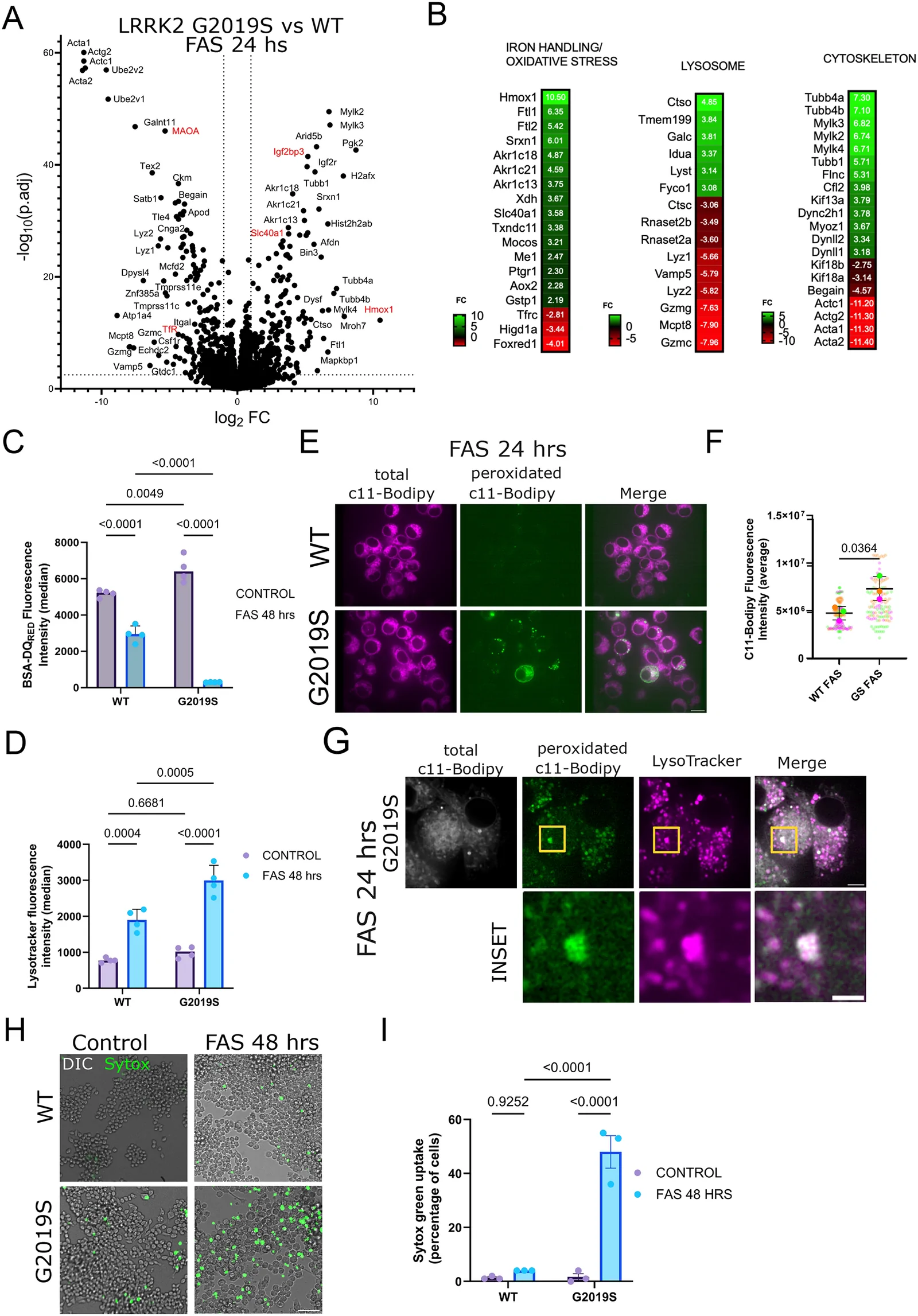
**LRRK2_G2019S_ cells are hypersensitive to iron-induced oxidation and ferroptotic cell death.** (A) Volcano plot showing differentially expressed proteins in LRRK2_G2019S_ compared to WT macrophages treated for 24 h with 100 μM FAS. On the *x*-axis, fold change (FC) and, on the *y*-axis, −log_10_[adjusted statistical significance (*P*_adj_)] are plotted. Dashed lines represent the cut-offs for analysis; proteins with |FC|>2.5 and *P*_adj_>1.3 (*P*<0.05) were considered as differentially expressed. Some of the confirmed hits shown in Fig. S4A are highlighted in red. (B) Heat maps depicting the fold change of proteins associated with iron handling and/or oxidative stress, lysosomal function, and cytoskeleton that are differentially expressed in iron-treated LRRK2_G2019S_ macrophages. Raw data are shown in Table S2. (C) Flow cytometric measurements of DQ Red BSA fluorescence intensity in WT and LRRK2_G2019S_ macrophages treated without (control) or with 100 μM FAS for 48 h. The median±s.d. of three biological replicates and *P*-values calculated using one-way ANOVA with Tukey's post hoc test for multiple comparisons are shown. (D) Flow cytometric measurements of LysoTracker Green fluorescence intensity in WT and LRRK2_G2019S_ macrophages treated without (control) or with 100 μM FAS for 48 h. The median±s.d. of three biological replicates and *P*-values calculated using one-way ANOVA with Tukey's post hoc test for multiple comparisons are shown. (E) Representative images of WT and LRRK2_G2019S_ macrophages treated for 24 h with 250 μM FAS and incubated with 1 μM Bodipy C11 for the detection of peroxidated lipids. Scale bar: 10 μm. (F) Quantification of integrated fluorescence intensity of peroxidated Bodipy C11. Cells analyzed per biological replicate: WT+FAS, *n*=44, 54, 49; LRRK2_G2019S_+ FAS, *n*=42, 51, 24. The mean±s.d. and single values of three separate experiments are shown. *P*-values from two-tailed unpaired *t*-test are shown. (G) Representative images of LRRK2_G2019S_ macrophages treated for 24 h with 250 μM FAS and incubated with 1 μM Bodipy C11 for the detection of peroxidated lipids. Lysosomes were stained with LysoTracker Deep Red (100 nM). Scale bars: 10 μm; 1 μm (insets). Images represent one of three independent experiments. (H) Representative images of WT and LRRK2_G2019S_ macrophages treated for 48 h with 500 μM FAS and incubated with SYTOX Green to identify dead cells. Differential interference contrast (DIC) images were used for total cell quantification. Scale bar: 100 μm. (I) Quantification of data shown in H. Data are shown as the percentage of cells positive for SYTOX Green. For each condition, a minimum of 30 cells were analyzed in each biological replicate. The mean±s.d. of three biological replicates and *P*-values calculated using one-way ANOVA with Tukey's post hoc test for multiple comparisons are shown.

In order to interrogate the global cellular consequences of LRRK2_G2019S_ mutation in conditions of iron overload, we returned to our initial proteomics approach. We obtained the total proteome from three biological replicates of WT and LRRK2_G2019S_ cells in untreated conditions, and following 24 h of FAS treatment (Table S2). Although FAS treatment induced many expected changes in WT cells, including reduced levels of TfR and increased levels of ferritins, there were many more robust changes in FAS-treated LRRK2_G2019S_ cells relative to their WT counterparts (Table S2; volcano plot in Fig. 4A). Gene Ontology analysis highlighted three functional groups that included changes in oxidative stress-related genes, lysosomal proteins and cytoskeletal signatures (Fig. 4B). For example, proteomics analyses identified a 10-fold increase in heme oxygenase 1 (HMOX1) in LRRK2_G2019S_ cells treated with FAS compared to WT cells, which we validated by western blotting, comparing to both WT and LRRK2 KO cells (Fig. S4A). HMOX1 is a well-studied transcriptional target of NRF2 (also known as NFE2L2) and part of the canonical oxidative stress response (Pham et al., 2025). Consistent with this, we also observed the nuclear translocation of NRF2 specifically in LRRK2_G2019S_ FAS-treated cells (Fig. S4B,C) and a block in cellular proliferation (Fig. S4D). These data indicate a significant increase in oxidative stress within mutant cells in the context of iron overload.

We were surprised to identify a reduction in monoamine oxidase A (MAOA) in LRRK2_G2019S_ cells, which we validated by western blotting (Fig. S4A). However, although specific to LRRK2_G2019S_ cells, the loss of MAOA was seen in both untreated and iron-overloaded LRRK2_G2019S_ macrophages, indicating that, although it is a very interesting observation, the loss of MAOA is unrelated to the iron phenotypes under investigation here. MAOA is an outer membrane enzyme primarily studied in neurons where it acts to degrade monoamine neurotransmitters such as serotonin, dopamine and epinephrine (Naoi et al., 2025), and its induced expression in tumor-associated macrophages has recently been shown to promote immunosuppression that promotes tumor progression (Ma et al., 2024; Wang et al., 2021). We also checked the specificity of another candidate, insulin-like growth factor 2 mRNA-binding protein 3 (IGF2BP3), which has been shown to regulate the mRNA stability of both iron- and stress-related targets including HMOX1 (Lv et al., 2025), STEAP3 (Liang et al., 2026), SLC7A11 (Yang et al., 2026) and FTH (Xu et al., 2022). In this case, IGF2BP3 levels were highly upregulated in both LRRK2_G2019S_ and LRRK2 KO lines, irrespective of iron overload (Fig. S4A).

The second global signature showed a specific downregulation of a series of lysosomal hydrolases including lysozymes and granzymes, with other lysosomal enzymes appearing to increase in LRRK2_G2019S_ iron-treated cells (Fig. 4B). To test the hydrolytic activity within lysosomes in these conditions, we used the proteolytic sensor DQ Red BSA, which is internalized into endolysosomes and becomes fluorescent upon cleavage by lysosomal hydrolyases (Marwaha and Sharma, 2017). In the steady state, there was little difference in lysosomal hydrolytic activity in LRRK2_G2019S_ macrophages relative to WT (Fig. 4C). Upon iron overload, we observed an ∼50% decrease in activity in control cells, as observed previously (Fernandez et al., 2016). This decrease was exacerbated in LRRK2_G2019S_ macrophages, showing an ∼90% reduction in hydrolytic capacity reported by the probe (Fig. 4C). This was not due to a lack of acidification, as demonstrated by LysoTracker probes that showed increased signal upon iron overload in both genotypes (Fig. 4D).

Taken together with the observation that the lysosomes within LRRK2_G2019S_ cells do not expand like they do in WT cells upon iron overload, and that there is a block in NCOA4 microautophagy (Fig. 2), the data indicate that the lysosomes within LRRK2_G2019S_ iron-loaded cells are dysfunctional. Very recent work has suggested that lysosomes in LRRK2_G2019S_ cells can accumulate ferrous iron, which could promote lipid oxidation (Mamais et al., 2025 preprint). We therefore used the Bodipy C11 lipid probe, oxidation of which shifts the wavelength to green, and observed an increased peroxidation of the probe within both internal membranes and at the plasma membrane (Fig. 4E, quantified in Fig. 4F). Upon co-staining with LysoTracker Deep Red, we further observed the oxidized lipids localizing to lysosomal membranes (Fig. 4G). Iron-induced peroxidation of lipids is known to be a driver of ferroptotic cell death. This can be detected upon uptake of SYTOX Green, a dye added to the medium that enters cells upon rupture of the cell surface. Indeed, we observed that ∼50% of LRRK2_G2019S_ cells were positive for SYTOX Green following 48 h of FAS treatment, whereas WT cells remained viable (Fig. 4H,I).

## DISCUSSION

The literature describing pathogenic LRRK2 variants in PD overwhelmingly supports a model in which a host of different variants across the protein all result in a gain-of-function kinase activity, which has focused clinical trials on LRRK2 kinase inhibitors. Previous studies have identified iron mishandling in LRRK2 mutant cells, and MRI approaches identify iron accumulation as a biomarker in patient carriers of the variant (Fernandez et al., 2022; Jia et al., 2023; Khan et al., 2024; Mamais et al., 2021, 2025 preprint; Martinez et al., 2023; Nazish et al., 2023; Oun et al., 2022). This work supports previous studies and further highlights dysregulated iron metabolism as a potential pathway of interest in LRRK2-related PD.

Our data indicate a primary dysfunction in lysosomal dynamics and function in iron-loaded LRRK2_G2019S_ cells, which do not undergo lysosomal enlargement and begin to lose hydrolytic activity. These organelles cannot promote the degradation of NCOA4, which then accumulates in the cytosol within detergent-insoluble foci. The accumulation of peroxidated Bodipy C11 within lysosomes is consistent with a recent preprint reporting that LRRK2_G2019S_ cells accumulate Fe^2+^ within lysosomes, which can result in lipid oxidation (Mamais et al., 2025 preprint). Our data do not resolve the earliest mechanisms to explain why the kinase-active LRRK2 prevents the dynamic changes within lysosomes that are triggered by iron overload. However, our experiments revealed that the increased LRRK2_G2019S_-dependent phosphorylation of Rab8 seen upon iron overload was reversed only with a type II inhibitor and was resistant to the type I inhibitor MLi-2 even though the inhibitor engaged LRRK2_G2019S_, as indicated by the loss of the pS1292 autophosphorylation mark. These results suggest that iron treatment alters the conformational landscape of LRRK2_G2019S_ differently, which could reflect LRRK2 as an iron-binding protein, or that a new protein interaction occurs in high-iron conditions that alters the conformation of the kinase domain. One study identified the NCOA4 ubiquitin ligase HERC2 as an LRRK2-binding protein (Imai et al., 2015), although no follow-up studies have confirmed this. One possibility is that LRRK2 could interfere with the capacity of HERC2 to degrade NCOA4.

In addition to this unusual feature of LRRK2 in iron-loaded cells, the appearance of pRab8 at the plasma membrane was unexpected given that the localization of LRRK2 has been documented primarily at the Golgi and lysosomes, particularly upon lysosomal damage. It is possible that this reflects LRRK2 recruitment to sites of plasma membrane damage following the increase in oxidized lipids, perhaps to repair the membrane, given this established function of LRRK2 (Bentley-DeSousa et al., 2025). This would suggest that pRab8 at the cell surface reflects a downstream consequence of altered lysosomal response to iron overload. Future experiments will focus on the potential function of LRRK2 at the cell surface and whether this localization could explain the differential sensitivity to the type I and type II kinase inhibitors.

### Limitations of the study

Lastly, our experiments are limited to the cultured macrophage RAW264.7 cell line. It is important to note that the impact of the LRRK2_G2019S_ variant on steady-state iron homeostasis appears to be highly cell and context specific. It was our proteomics analysis that initially identified the loss of FTH and increased FTL within LRRK2_G2019S_ RAW264.7 macrophages. However, single-cell RNA sequencing experiments from LRRK2_G2019S_ mice showed that most cell types had no alteration in iron-related genes, with the noted exception of microglia, some astrocyte cell types and oligodendrocytes, with neurons showing no change (Khan et al., 2024). Functional experiments examining iron homeostasis in LRRK2 heterozygous mutant iPSC-derived lines have also seen changes in ferrous iron accumulation in lysosomes (Mamais et al., 2025 preprint), and in mice showing an induction of iron dysregulation in microglia upon lipopolysaccharide treatment (Mamais et al., 2021). In this context, our work adds some critical new insight into the consequence of the kinase-active LRRK2 on the processes of ferritinophagy and degradation of NCOA4, highlighting an unexpected localization of pRab8 to the plasma membrane. The current study does not resolve the underlying mechanisms and context-specific actions of LRRK2 in iron metabolism. For now, we expand the experimental foundation for further exploration of the impact of LRRK2 in conditions of iron overload. Given the historical focus on iron toxicity within the SNpc and *locus coeruleus* of patients with PD, this might be an important physiological niche where LRRK2 variants play pathological roles. With the ongoing clinical trials focused on LRRK2 kinase inhibitors, it will be important to monitor their efficacy with biomarkers linked to iron metabolism, perhaps including quantitative susceptibility mapping MRI (Guan et al., 2024; Martinez et al., 2023).

## MATERIALS AND METHODS

### Cell culture and reagents

RAW264.7 WT (TIB-71, American Type Culture Collection, RRID:CVCL_0493), RAW264.7 LRRK2_G2019S_ (RRID:CVCL_F1ZX), RAW264.7 mCherry–FTL (RRID:CVCL_F2A0), RAW264.7 mCherry–NCOA4 (RRID:CVCL_F2A2), RAW264.7 TMEM192–HA (RRID:CVCL_F2A4) and RAW264.7 LRRK2 KO (RRID:CVCL_F4C6) cells were cultured in Dulbecco's modified Eagle medium (DMEM) containing high glucose, GLUTA-PLUS and sodium pyruvate (Wisent, 319-027-CL) supplemented with 10% heat-inactivated fetal bovine serum (Wisent, 098159) and antibiotics (Gibco, 15-140-122). Cells were cultured at 37°C and 5% CO_2_ in a humidified incubator and regularly tested for mycoplasma contamination. Biohazard permits, training and licensing were obtained through the Environmental Health and Safety Offices of McGill University.

Bafilomycin A1 (B1793), ammonium iron (II) sulfate hexahydrate (FAS) (09719), deferoxamine mesylate salt (DFO) (D9533) and LLOME (L7393) were purchased from Millipore-Sigma. MLi-2 was purchased from Abcam (ab254528). Rebastinib was purchased from MedChemExpress (HY-13024). RN277 and RN341 were kindly provided by Samara Reck-Peterson and Andres Leschziner (Weill Cornell Medicine, NY, USA).

### LRRK2_G2019S_ proteomics analysis

Samples were reconstituted in 50 mM ammonium bicarbonate with 10 mM tris(2-carboxyethyl) phosphine hydrochloride (TCEP; T2556, Thermo Fisher Scientific) and vortexed for 1 h at 37°C. Chloroacetamide (C0267, Sigma-Aldrich) was added for alkylation to a final concentration of 55 mM. Samples were vortexed for another hour at 37°C. 1 μg of trypsin was added, and digestion was performed for 8 h at 37°C. Samples were dried down and solubilized in 5% acetonitrile with 4% formic acid. The samples were loaded on a 1.5 μl pre-column (Optimize Technologies, Oregon City, OR, USA). For each run, 1 μg of peptides was separated on a home-made reversed-phase column (150 μm internal diameter, 200 mm height) with a 116-min gradient from 10 to 30% acetonitrile with 0.2% formic acid and a 600 nl/min flow rate on an Easy nLC-1200 connected to a Exploris 480 mass spectrometer (Thermo Fisher Scientific, RRID:SCR_022215) with the high-field asymmetric-waveform ion mobility spectrometry (FAIMS) interface. Each proteome was analyzed with four separate runs of liquid chromatography (LC) followed by tandem mass spectrometry (MS/MS) acquired with a different set of sequential compensation voltages (CVs) and a different range of mass-to-charge ratios (*m/z*): run 1 was performed with the CV set −44 V, −54 V, −64 V and −74 V from *m/z* 350 to 453; run 2 was performed with the CV set −40 V, −48 V, −56 V, −64 V, −72 V and −80 V from *m/z* 451 to 542; run 3 was performed with the CV set −40 V, −48 V, −56 V, −64 V, −72 V and −80 V from *m/z* 540 to 661; and run 4 was performed with the CV set −35 V, −46 V and −57 V from *m/z* 659 to 890. Each full mass spectrum acquired at a resolution of 120,000 was followed by MS/MS spectra acquisition on the most abundant multiply charged precursor ions for 3 s. MS/MS experiments were performed using higher-energy collision dissociation (HCD) at a collision energy of 34% with an automatic gain control of 50%, a resolution of 15,000 and an injection time of 28 ms. The data were processed using PEAKS X Pro (Bioinformatics Solutions, https://www.bioinfor.com/peaks-xpro/, RRID:SCR_022841) and a UniProt mouse database (16977 entries, https://www.uniprot.org/taxonomy/10090, accessed 17 May 2024) with trypsin as the digestion mode. Mass tolerances on precursor and fragment ions were 10 ppm and 0.01 Da, respectively. The fixed modification was carbamidomethyl. Variable selected post-translational modifications were acetylation, oxidation, deamidation and phosphorylation.

### Cell lysates, SDS-PAGE and immunoblotting

Cells were collected by scraping, and they were washed twice with ice-cold PBS buffer and resuspended in 1× PBS containing protease inhibitors (Roche, 04693132001) and phosphatase inhibitors (Roche, 04906837001). Total protein content was quantified by Bradford assay (Bio-Rad, 5000006). Equal quantities of samples were lysed in Laemmli buffer [10% (v/v) glycerol, 2% (w/v) SDS, 0.005% (w/v) Bromophenol Blue, 60 mM Tris-HCl pH 6.8 and 10% (v/v) β-mercaptoethanol] and heated for 5 min at 95°C prior to loading on gels for SDS-PAGE. Proteins were size separated and transferred to nitrocellulose membranes (Bio-Rad, 1620097), blocked for 1 h in blocking solution [PBS containing 0.5% (v/v) Tween 20 (PBS-T) and 5% (w/v) skim milk]. After blocking, the membranes were incubated overnight with primary antibody solution (1:1000 dilution of the antibodies in PBS-T unless otherwise specified). After primary antibody incubation, membranes were washed five times for 10 min in PBS-T and incubated for 1 h with HRP-conjugated secondary antibodies, sheep anti-mouse (Cytiva, NA931, RRID:AB_772210) and donkey anti-rabbit (Cytiva, NA934, RRID:AB_772206) in blocking solution (1:5000). Finally, membranes were washed and developed using INTAS ChemoCam (INTAS Science Imaging) using Western-Lightning PLUS-ECL (Revvity, NEL105001EA). Uncropped blots are included in the blot transparency section (Fig. S5).

#### Antibodies

The following rabbit antibodies were used for western blotting: anti-FTL (Proteintech, 10727-1-AP, RRID:AB_2278673), anti-FTH (Cell Signaling Technology, 4393, RRID:AB_11217441), anti-LRRK2 (Abcam, ab133474, RRID:AB_2713963; or Abcam, ab133518, RRID:AB_2938806), anti-GAPDH (Santa Cruz Biotechnology, sc-25778, RRID:AB_10167668), anti-IRP2 (Proteintech, 29976-1-AP, RRID:AB_3086201), anti-pLRRK2_S1292_ (Abcam, ab203181, RRID:AB_2921223), anti-NCOA4 (Bethyl Laboratories, A302-271A, RRID:AB_1850158), anti-TOMM40 (Proteintech, 18409-1-AP, RRID:AB_2303725), anti-ferroportin (Alomone Labs, AIT-001, RRID:AB_2340981), anti-pRab8A (1:250) (Abcam, ab230260, RRID:AB_2814988), anti-Rab8A (1:250) (Cell Signaling Technology, cs-6975, RRID:AB_10827742), anti-AKT (Cell Signaling Technology, cs-9272, RRID:AB_329827), anti-pAKT_S473_ (Cell Signaling Technology, cs-4060, RRID:AB_2315049), anti-HMOX1 (Proteintech, 10701-1-AP, RRID:AB_2122782), anti-MAOA (Proteintech, 10539-1-AP, RRID:AB_2137251) and anti-IGF2BP3 (Proteintech, 10539-1-AP, RRID:AB_2137251).

The following mouse antibodies were used for western blotting: anti-TfR (Invitrogen, 136800, RRID:AB_2533029), anti-HERC2 (BD Biosciences, 612366, RRID:AB_399728), anti-FBXL5 (Santa Cruz Biotechnology, sc-390102), anti-DMT1 (Santa Cruz Biotechnology, sc-166884, RRID:AB_10610255), anti-β-actin (Santa Cruz Biotechnology, sc-8432, RRID:AB_626630), anti-PCNA (Thermo Fisher Scientific, 13-3900, RRID:AB_2533016), anti-vinculin (Sigma-Aldrich, V4505, RRID:AB_477617) and anti-α-tubulin (Santa Cruz Biotechnology, sc-23948, RRID:AB_628410).

### Iron quantification

Fe^2+^, Fe^3+^ and total iron content were quantified using the colorimetric iron assay kit from Abcam (ab83366) according to the manufacturer's instructions. Briefly, 2.5×10^6^ cells were seeded in six-well plates and treated with 100 μM FAS for 16 h. Each sample was lysed in 120 μl of assay buffer, and 50 μl was separated into two wells for quantification of Fe^2+^ and Fe^3+^ content. The rest of the sample was kept for protein concentration estimation. Iron content was obtained by measuring the absorbance at 593 nm and the values were interpolated into the standard curve and then normalized to protein concentration.

### Aconitase activity assay

Aconitase activity was quantified using the colorimetric aconitase activity assay kit (Abcam, ab83459) according to the manufacturer's instructions. Briefly, 2.5×10^6^ cells were harvested, washed with PBS and resuspended in assay buffer. Insoluble materials were cleared by centrifuging samples at 800 ***g***, the resulting fraction was further centrifuged at 20,000 ***g*** for 15 min and the supernatant was used to quantify cytoplasmic aconitase activity. Aconitase activity was measured colorimetrically by measuring absorbance at 450 nm, and the enzymatic activity was calculated based on the standard curve and normalized to protein concentration.

### Generation of CRIPSR knock-in/KO and stable cell lines

#### LRRK2_G2019S_ cell lines

The gRNA for generation of homozygous LRRK2_G2019S_ was designed using TrueDesign Genome editor and synthesized by Thermo Fisher Scientific (sequence 5ʹ-U*U*G*CGAAGAUUGCGGACUAC-3ʹ+modified scaffold; asterisks represent phosphorothioate linkages). The LRRK2_G2019S_ homology-directed repair (HDR) template used was 5′-G*T*A*AACTTGTTCTCCTACCTGGGGTGCCCTCTGATGTCTTTATTCCCATCCTGCAGCAGTACTGTGCGATCGAGTAGTCCGCAATCTTCGCAATGATGGCAGCATTGGGATACAGGGTAAAAAGCAGCACATTGTGGGGCTTCAGGTCACGGTAAATAATCATGGCTGAGTGG*A*G*A-3′. Raw cells were scraped, washed with PBS and electroporated with Cas9 protein and donor HDR templates using the Neon transfection system (MPK5000, Thermo Fisher Scientific) according to the manufacturer's instructions. After electroporation, cells were plated and cultured for 4 days. Afterwards, single cells were seeded in 96-well plates, and genomic DNA was isolated from single clones. The gRNA target region was amplified by PCR using AmpliTaq Gold 360 master mix (4398881, Thermo Fisher Scientific). PCR amplicons were sequenced using standard Sanger sequencing (Fig. S1A).

#### CRISPR KO of *Lrrk2*

To disrupt the *Lrrk2* loci, gRNAs were designed using the webtool CHOPCHOP (https://chopchop.cbu.uib.no; Labun et al., 2019), and exons 1 and 7 of mouse *Lrrk2* gene were targeted. sgRNA exon1 5ʹ-GTGTTCACCTACTCGGACCGCGG-3ʹ and sgRNA exon 7 5ʹ-TGTCCGGTGCTACAATCTTGTGG-3ʹ were purchased from Synthego. Ribonucleoparticles (RNPs) were assembled by incubating gRNAs with Cas9 enzyme, followed by reverse transfection using Lipofectamine RNAiMax transfection reagent (Invitrogen, 13778150) diluted in Opti-MEM (Gibco, 31985070), according to the manufacturers’ instructions. Approximately 75,000 cells were seeded in 24-well plates together with RNP transfection solution. 24 h post transfection, single cells were seeded in 96-well plates and several colonies were monitored for the expression of LRRK2. One clone was isolated with a 2 bp deletion in exon 1 detected by Sanger sequencing.

### Stable cell lines

Stable cells lines expressing mCherry–FTL and mCherry–NCOA4 were generated using the retroviral transduction system and stably integrated into the genome. Using the Gateway system (11791019, Thermo Fisher Scientific), cDNAs of human *FTL* (subcloned from Addgene #100153) and human *NCOA4* (DNASU plasmid repository, HsCD00043681; https://dnasu.org/DNASU/Home.do) were cloned into the plasmid pBABE-puro-mCherrry-GTW, which was engineered by cloning mCherry2 (Addgene #54563) into pBABEpuro-gateway (Addgene #51070). For the generation of the retroviral particles, HEK293 phoenix cells (American Type Culture Collection, CRL-3213, RRID:CVCL_H716) were transfected with the different constructs using jetPrime transfection reagent (Polyplus, 101000027). The medium of the cells was collected 48 h post transfection, passed through a 0.45 μm filter and transduced into the macrophages. After 24 h of viral transduction, the medium was changed and cells selected with 10 μg/ml puromycin until the appearance of resistant colonies. Stable cell lines were used as polyclones.

Macrophage cells stably expressing TMEM192–HA (Addgene #102930) were generated using lentivirus. Lentiviral particles were generated in HEK293T cells using the Lenti-X packaging system (Takara, 631275) according to the manufacturer’s instructions. After 36 h, the medium was collected, passed through a 0.45 μm filter and added to the RAW264.7 macrophages. 24 h post transduction, cells were selected in 10 μg/ml puromycin until the appearance of resistant colonies. TMEM192–HA expression was confirmed by western blotting, and polyclonal cell lines were used in this study.

### Immunofluorescence and live-imaging conditions

Cells were seeded in 13 mm sterile glass coverslips (Thermo Fisher Scientific, 174950), fixed with 6% paraformaldehyde for 20 min at 37°C, washed three times with PBS, and permeabilized with 0.1% Triton X-100 in PBS for 20 min. After permeabilization, cells were blocked for 30 min at room temperature in PBS containing 5% BSA and probed overnight at 4°C with primary antibodies. After incubation with the secondary antibodies, slides were washed three additional times with PBS, stained with DAPI (Invitrogen, D1306) and washed with double-distilled water. Finally, coverslips were mounted on slides using fluorescent mounting medium (Dako, S302380-2) prior to imaging.

The following antibodies were used for immunofluorescence: rabbit anti-LAMP1 (1:1000, Abcam, ab208943, RRID:AB_2923327), rabbit anti-pRab8 (1:250, Abcam, ab230260, RRID:AB_2814988), rabbit anti-NRF2 (1:1000, Cell Signaling Technology, 12721, RRID:AB_2715528), mouse anti-HA (1:1000, Sigma-Aldrich, H9658, RRID:AB_260092) and rat anti-LAMP1 (1:1000, Developmental Studies Hybridoma Bank, 1D4B, RRID:AB_528127).

For pRab8 staining, after paraformaldehyde fixation, cells were permeabilized with ice-cold methanol for 20 min at −20°C before blocking. After washing and incubation with primary antibodies, cells were incubated with the appropriate secondary antibodies (1:3000) – donkey anti-rabbit coupled to Alexa Fluor 488 (Invitrogen, A11006, RRID:AB_2534114) or Alexa Fluor 647 (Invitrogen, 21244, RRID:AB_2535812), donkey anti-rat coupled to Alexa Fluor 488 (Invitrogen, A11070, RRID:AB_2534074) or Alexa Fluor 647 (Invitrogen, 21247, RRID:AB_141778), or goat anti-mouse coupled to Alexa Fluor 488 (Invitrogen, A11029, RRID:AB_2534088).

For live imaging, approximately 100,000 cells were seeded in glass-bottomed imaging plates (Cellvis, D35C4-20-1.5-N) and stained using normal growing medium supplemented with 20 mM HEPES for pH stabilization. For FerroOrange (Dojindo, F374) staining, cells were incubated for 1 h in 1 μM FerroOrange in complete medium prior to imaging. For labeling late endosomes and lysosomes, cells were incubated in 100 nM LysoTracker Green (Thermo Fisher Scientific, L7526), LysoTrackerRed (Thermo Fisher Scientific, L7528) or LysoTracker Deep Red (Thermo Fisher Scientific, L12492) for 30 min prior to imaging. To quantify proteolytic activity, control or treated cells were incubated for 1 h with 10 μg/ml DQ Red BSA (Thermo Fisher Scientific, D12051) combined with 100 nM LysoTracker Green. For the assessment of lipid peroxidation, cells were incubated for 1 h with 1 μM Bodipy 581/591 C11 (Thermo Fisher Scientific, D3861). For time-lapse microscopy, a rate of one frame every 2 s was acquired. After staining with different dyes, cells were then imaged under controlled temperature and humidity conditions. Cell death was assessed by staining the cells with 1 μM SYTOX Green (Thermo Fisher Scientific, S34860). Immunofluorescence and live images were acquired using an Olympus IX83 inverted microscope (RRID:SCR_020344) with Yokogawa spinning disk confocal microscope (RRID:SCR_020907) using an UPLANS APO 100×/1.40 NA oil objective an Andor iXon Ultra EMCCD camera (RRID:SCR_023166). All the samples were measured under same acquisition and laser intensity conditions.

### Quantification of FTL subcellular distribution

Distribution of mCherry–FTL was classified into four categories: (1) cytosolic – most of the FTL signal was homogenously distributed across the cellular cytoplasm; (2) lysosomal – the brightest signal inside the cell colocalized with LAMP1 or LysoTracker-positive structures; (3) cytosolic and lysosomal – the FTL signal was largely diffuse and dim structures positive for LAMP1 or LysoTracker could be detected; and (4) cytosolic with condensates – the FTL distribution was largely diffuse and cytoplasmic but accumulation of bright LAMP1 or LysoTracker-negative structures was detected.

### Biochemical fractionation of Triton X-100-soluble and -insoluble fractions

To fractionate Triton X-100-soluble fractions from insoluble fraction biochemically, cells were first resuspended in PBS, protein concentration was estimated, equal amounts of different samples were centrifuged at 600 ***g*** for 5 min, and cell pellets were resuspended in Triton X-100 lysis buffer (50 mM HEPES pH 7.4, 150 mM NaCl, 1%, Triton X-100, 1 mM EDTA) supplemented with protease and phosphatase inhibitors (referenced in the ‘Cell lysates, SDS-PAGE and immunoblotting’ section). Cell lysates were vortexed thoroughly and incubated for 10 min on ice, followed by centrifugation at 20,000 ***g*** at 4°C for 20 min. The supernatant was transferred to a fresh tube and diluted in Laemmli buffer to generate 1× Triton X-100-soluble fraction, whereas the remaining Triton X-100-insoluble pellet was resuspended in 1× Laemmli sample buffer to the same final volume of the soluble fraction. Samples were then analyzed by SDS-PAGE.

### Quantification of condensate number and size with image analysis

We used FIJI (http://fiji.sc, RRID:SCR_002285) to quantify the number and area of extra-lysosomal condensates. Images of fixed cells expressing either mCherry–NCOA4 or mCherry–FTL and the lysosomal marker LAMP1 were acquired using the same laser and exposure conditions and analyzed with the following workflow. This is a cell-based analysis, so first, single cells were selected with a region of interest (ROI), while the rest of the field was excluded from the analysis. Next, the LAMP1 channel was selected, and the background was subtracted and binarized by automatic thresholding using the Otsu method (Otsu, 1979) to obtain a lysosomal mask. Using this lysosomal mask, particle analysis was performed and the ROIs obtained were overlayed with the channel of interest (mCherry–NCOA4 or mCherry–FTL), and all the pixels inside these ROIs (lysosomes) were deleted. Finally, the resulting image of the channel of interest was again background subtracted and binarized by automatic thresholding to obtain a mask, for which particle analysis was performed to quantify the number and size of extra-lysosomal structures.

### Quantification of the integrated fluorescence intensity of FerroOrange and Bodipy C11

FerroOrange or Bodipy C11 were imaged in live cells using confocal microscopy and the fluorescence intensities were quantified by image analysis using FIJI. Several cells per field were selected and their integrated fluorescence intensity was measured. The experiment was repeated four times.

### Quantification of lysosomal size and number with image analysis

We used FIJI to analyze and quantify the number and size of lysosomes in single planes. It is important to note that RAW264.7 macrophages are not flat cells; thus, the number of lysosomes only represents single-plane information and not the total cellular volume. For the analysis of lysosomes in LRRK2_G2019S_ macrophages, we used cells expressing TMEM192–HA due to its high levels of expression and clear distribution in the lysosomal membrane, allowing better edge detection. Briefly, images were acquired under the same laser power and time exposure conditions and, afterwards, analyzed with the following workflow. First, single cells were selected with a ROI, while the rest of the field was excluded from the analysis. Next, the HA or LysoTracker Green channel was selected, the background was subtracted and binarized by automatic Otsu thresholding to obtain a lysosomal mask. Using this lysosomal mask, particle analysis was performed, and the average number and size of lysosomes were recorded per studied cell.

### Quantification of lysosomal proteolytic activity by flow cytometry

Cells were seeded and treated with 100 μM FAS for 48 h or left untreated as the control. To quantify lysosomal proteolytic activity, cells were stained with 10 μg/ml of DQ Red BSA and 100 nM LysoTracker Green for 1 h, under the same conditions described in the ‘Immunofluorescence and live-imaging conditions’ section. After staining, cells were collected and transferred to a fresh tube in a suspension of 1×10^6^ cells/ml. For acquisition, single-cell suspensions were acquired on an Attune NxT flow cytometer (RRID:SCR_019590, Thermo Fisher Scientific). The ‘Cytometer Setup & Tracking’ (CS&T) performance-tracking protocol was run prior to cell acquisition as per the manufacturer's recommendation. Photomultiplier tube voltages were determined by daily CS&T performance tracking. Compensation controls were also acquired. 50,000 events were acquired per sample. Finally, the median DQ Red BSA fluorescence and median LysoTracker Green fluorescence from three different repetitions were calculated and are shown in the figures.

### Quantification of pRab8 and NRF2 subcellular distribution

Quantification of pRab8 and NRF2 distribution was performed qualitatively. Briefly, control or FAS-treated cells were stained with endogenous antibodies, and signals from the channel corresponding to the studied protein were equally thresholded among all the cell lines and treatments. The number of cells displaying bright detectable pRab8 signal at the plasma membrane or NRF2 signal at the nucleus were counted as positive, and the total number of cells assessed by DAPI nuclear staining was used to estimate the percentage of positively-stained cells. Experiments were repeated at least three times with a minimum of 30 cells per condition.

### Growth curves

To assess growth, on day 0, 150,000 cells were seeded in 12-well plates and, on day 1, cells were either left untreated for control or incubated with 100 μM FAS. Quadruplicate measurements were performed in each condition, and cell number was followed for a period of 72 h.

### Statistical analysis

All experiments were performed independently at least three times and quantifications and/or representative images are provided. For each condition, a minimum of 20 cells were analyzed in all biological replicates; the precise number of cell number per replicate is included in raw data files (https://doi.org/10.5281/zenodo.19374070). One-way ANOVA followed by Tukey's honestly significant difference (HSD) post hoc test was performed using GraphPad Prism software (http://www.graphpad.com/, version 8.4.3, RRID:SCR_002798). Experiments with more than two samples included multiple comparison analysis. The arithmetic means with standard deviation of at least three independent experiments are depicted. In addition, the values of all individual cells and the means of each experiment are shown for the quantifications of size and number of condensates as well as lysosomes. No statistical method was used to predetermine the sample size. The experiments were not randomized and the investigators were not masked to allocation during experiments and outcome assessment.

## Supplementary Material



10.1242/joces.264977_sup1 Supplementary information

Table S1. Proteomics dataset from LRRK2G2019S and wild type RAW macrophages.

Table S2. Proteomics dataset from LRRK2G2019S and wild type RAW macrophages FAS treated macrophages.
