# Supplementary material for: An LRRK2 variant blocks NCOA4 trafficking upon iron overload, leading to ferroptotic death
Source: J Cell Sci. 2026 Jul 27;139(14):jcs264977. doi: 10.1242/jcs.264977 (PMC13490738; doi:10.1242/jcs.264977)
Supplement: Supplementary file 1 [file joces-139-264977-s1.pdf]

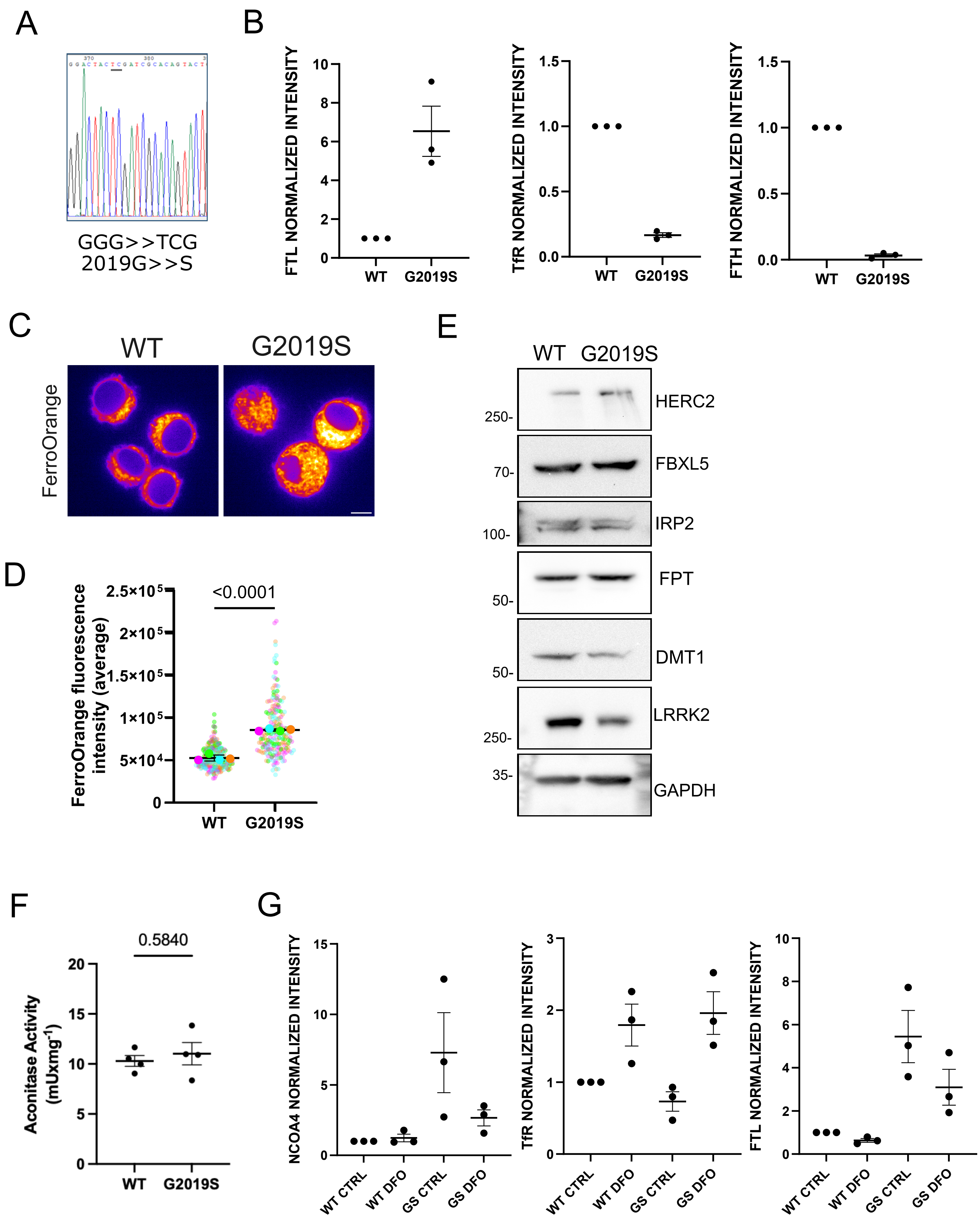

Journal of Cell Science • Supplementary information

Fig. S1. LRRK2$_{G2019S}$ RAW macrophages show changes in iron homeostasis

A- Confirmation by Sanger sequencing of LRRK2$_{G2019S}$ knock-in generation in RAW macrophages. **B**- Densitometry analysis of FTH, FTL and TfR levels in WT and LRRK2$_{G2019S}$ macrophages. Normalized band intensities ± SD of three separate biological replicates are shown. **C**- Representative images of WT and LRRK2 $_{G2019S}$ macrophages stained with Fe^{2+} sensor FerroOrange. Fluorescence intensity of the images was color coded using FIJIs thermal LUT. Scale bar 5 µm. **D**- Quantification of FerroOrange fluorescence intensity of data shown in C. Cells analyzed per biological replicate: WT control N=80, 58, 59, 78, LRRK2$_{G2019S}$ control N= 56, 56, 51 and 42 . The means ± SD and single values of three biological replicates are shown. T-test statistical analysis is shown. **E** - Western blot analysis on whole cell lysates of IRP2, FBXL5, HERC2, ferroportin (FPT) and DMT1 in WT and LRRK2$_{G2019S}$ macrophages. GAPDH serves as loading control. **F**- Quantification of Aconitase activity in WT and LRRK2$_{G2019S}$ RAW macrophages, expressed as activity units per mg of protein. The means ± SD of three biological replicates and T-test statistical analysis are shown. **G -** Densitometry analysis of FTL, TfR and NCOA4 levels in WT and LRRK2$_{G2019S}$ macrophages control or treated for 24 hours with 250 µM DFO. Normalized band intensities ± SD of three separate biological replicates are shown.

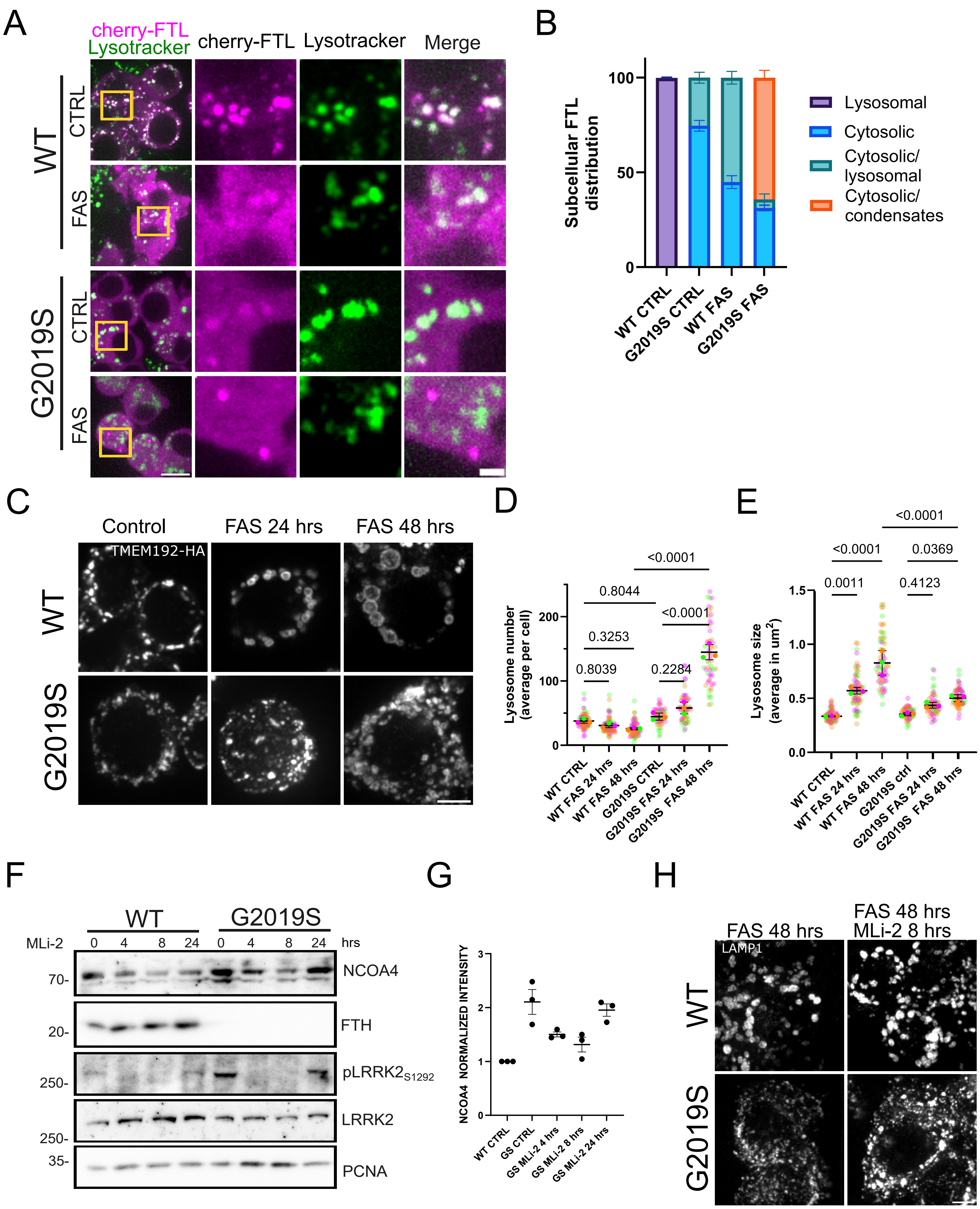

Journal of Cell Science • Supplementary information

Fig. S2. $LRRK2_{G2019S}$ show defects in NCOA4 turnover upon iron overload

A- Representative images of cherry-FTL distribution in WT and $LRRK2_{G2019S}$ macrophages control or treated for 16 hours with FAS 100 µM. Lysosomes were stained with Lysotracker green. Scale bar 4 µm, inset 1 µm. **B**- Qualitative classification of cherry-FTL distribution of data shown in A. Cells analyzed per biological replicate: WT control N=107, 97, 134, $LRRK2_{G2019S}$ control N= 89, 59, 122, WT FAS N=131, 149, 165, $LRRK2_{G2019S}$ FAS N= 81, 77, 92. The means ± SD of three biological replicates are shown. **C**- Representative images of lysosomal morphology of WT and $LRRK2_{G2019S}$ macrophages control or treated for 24 or 48 hours with FAS 100 µM. Cells expressing TMEM192-HA were stained with anti-HA antibody. Scale bar 4 µm. **D**- Quantification of average number of lysosomes per cell of the experiment shown in C. Cells analyzed per biological replicate: WT control N=24, 26, 27, $LRRK2_{G2019S}$ control N= 21, 20, 20, WT FAS N=27, 28, 20, $LRRK2_{G2019S}$ FAS N= 19, 25, 18. The means ± SD and single values of three biological replicates are shown. Statistical analysis by one-way ANOVA multiple comparison. **E**- Quantification of the average lysosomal size per cell of data shown in C. For each biological replicate the number of cells is the same as stated in part D. The means ± SD and single values of three biological replicates are shown. Statistical analysis by one-way ANOVA multiple comparison. **F**- Western blot analysis of FTH and NCOA4 levels on whole cell extracts of WT and $LRRK2_{G2019S}$ macrophages control or treated for 4, 8 or 24 hours with 1 µM MLi-2. Phosphorylation levels of LRRK2 on S1292 serves as positive control, PCNA serves as loading control. **G-** Densitometry analysis of NCOA4 levels in $LRRK2_{G2019S}$ cells treated with1 µM MLi-2 for 4, 8 or 24 hours. Normalized band intensities ± SD of three biological replicates are shown. **H**- Representative images of lysosome morphology of WT and $LRRK2_{G2019S}$ macrophages treated for 48 hours with FAS 100 µM and then incubated for 8 hours with 1 µM MLi-2 in the presence of iron. Lysosomes were stained with anti LAMP1 antibody. Scale bar 5 µm.

Journal of Cell Science • Supplementary information

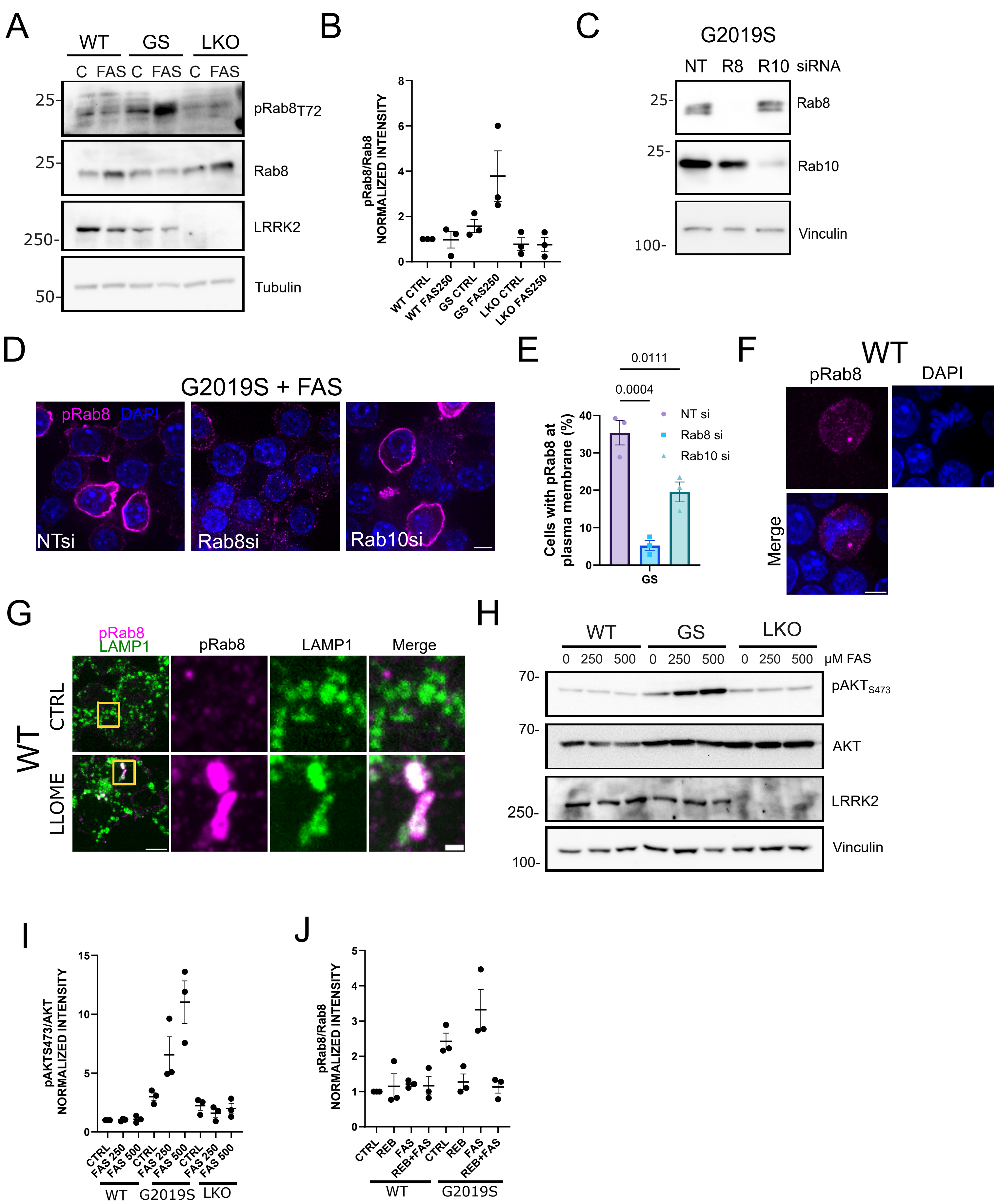
A
WT
GS
LKO
C
FAS
C
FAS
C
FAS
25-
pRab8T72
25-
Rab8
250-
LRRK2
50-
Tubulin
B
pRab8/Rab8 NORMALIZED INTENSITY
WT CTRL
WT FAS250
GS CTRL
GS FAS250
LKO CTRL
LKO FAS250
C
G2019S
NT
R8
R10
siRNA
25-
Rab8
25-
Rab10
100-
Vinculin
D
G2019S + FAS
pRab8
DAPI
NTsi
Rab8si
Rab10si
E
0.0111
0.0004
NT si
Rab8 si
Rab10 si
Cells with pRab8 at plasma membrane (%)
GS
F
WT
pRab8
DAPI
Merge
G
pRab8
LAMP1
pRab8
LAMP1
Merge
WT
CTRL
LLOME
H
WT
GS
LKO
0
250
500
0
250
500
0
250
500
µM FAS
70-
pAKTS473
70-
AKT
250-
LRRK2
100-
Vinculin
I
pAKTS473/AKT NORMALIZED INTENSITY
CTRL
FAS 250
FAS 500
WT
G2019S
LKO
J
pRab8/Rab8 NORMALIZED INTENSITY
CTRL
REB
FAS
REB+FAS
WT
G2019S

Fig. S3. LRRK2$_{G2019S}$ impact on iron metabolism reveals differential sensitivity to type I and type II kinase inhibitors.

A-Western blot analysis of pRab8 in WT, LRRK2$_{G2019S}$ and LRRK2 KO macrophages control or treated with FAS 250 µM for 16 hours. Tubulin serves as loading control. **B**-Densitometry quantification of data shown in A. Band intensities ± SD of three biological replicates are shown. **C**- Western blot analysis of Rab8 or Rab10 levels in LRRK2$_{G2019S}$ treated with non-targeting (NT), Rab8 (R8) or Rab10 (R10) siRNA. Vinculin serves as loading control. **D**-Representative images of endogenous pRab8A distribution in LRRK2$_{G2019S}$ macrophages treated for 72 hours with NT, Rab8 or Rab10 siRNA and treated for 24 hours with FAS 250 µM. DNA is stained with DAPI. Scale bar 10 µm. **E**-Quantification of the percentage of cells showing positive pRab8 signal at plasma membrane of data shown in D. Cells analyzed per biological replicate: LRRK2$_{G2019S}$ FAS +NTsi N=125, 149, 105, LRRK2$_{G2019S}$ FAS +Rab8si N=192, 141, 119, LRRK2$_{G2019S}$ FAS +Rab10si N=135, 131, 145. The means ± SD of three biological replicates and one-way ANOVA multiple comparison statistical analysis are shown. **F**- Representative images of endogenous pRab8A distribution in mitotic WT macrophages. DNA is stained with DAPI. Scale bar 5 µm. **G**- Representative images of endogenous pRab8A distribution in WT macrophages control of treated for 3 hours with LLOME 1 mM. Lysosomes were stained with anti LAMP1 antibody. Scale bar 5 µm. **H**-Western blot analysis of pAKT S473 in WT, LRRK2$_{G2019S}$ and LRRK2 KO macrophages control or treated with 250 or 500 µM FAS for 24 hours. Vinculin serves as loading control. **I**- Densitometry quantification of data shown in H. Normalized band intensities ± SD of three biological replicates are shown. **J**- Densitometry quantification of pRab8 levels by western blot analysis of WT and LRRK2$_{G2019S}$ macrophages control or treated with 1 µM rebastinib for two hours and then left untreated or exposed to FAS 250 µM for 16 hours in the presence of the inhibitor. Normalized band intensities ± SD of three biological replicates are shown.

Journal of Cell Science • Supplementary information

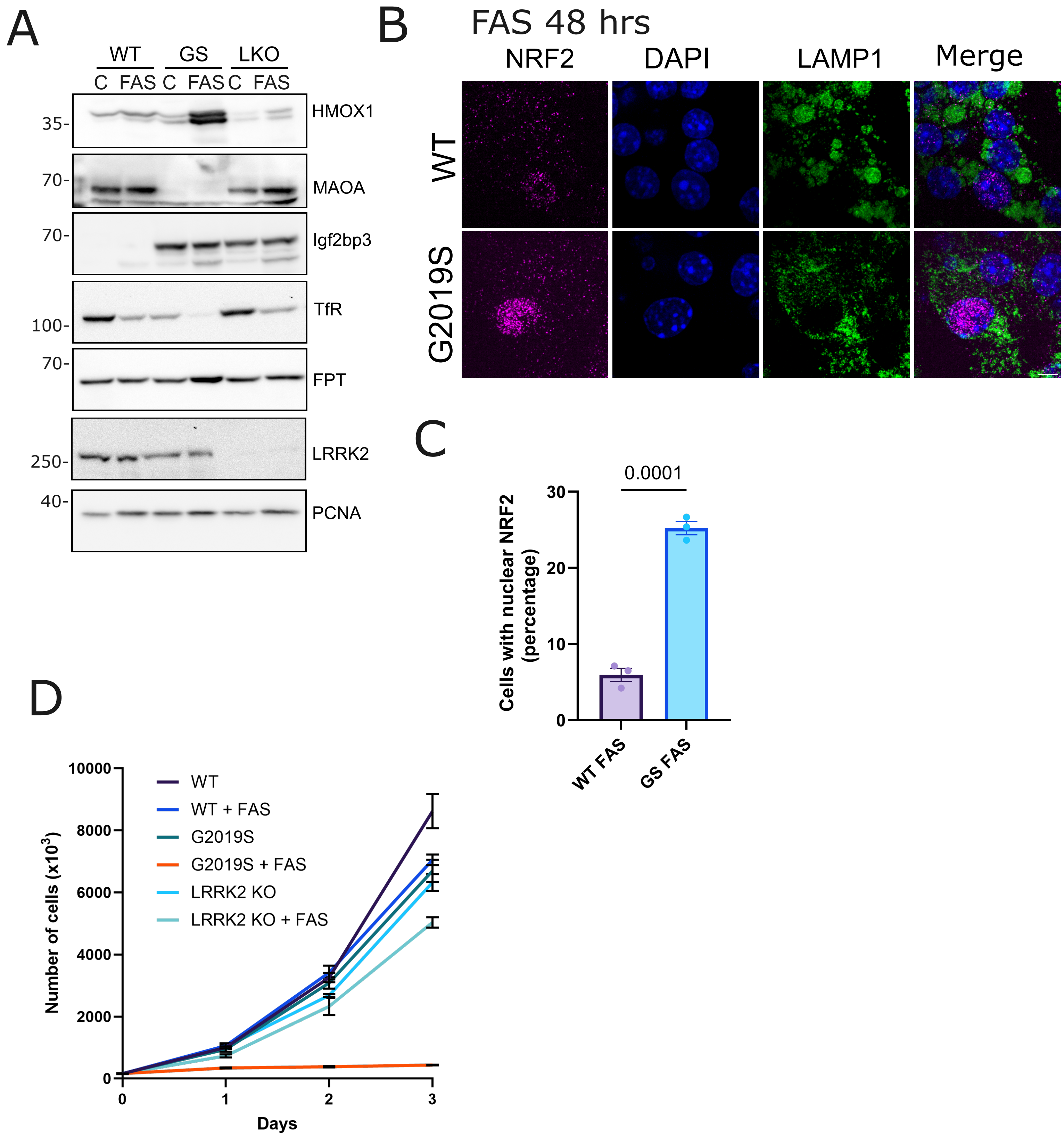

Journal of Cell Science • Supplementary information

Fig. S4. LRRK2$_{G2019S}$ cells are hypersensitive to iron induced oxidation and ferroptotic cell death.

A- Confirmative western blot analysis of selected differentially expressed proteins in iron treated LRRK2$_{G2019S}$ macrophages compared to WT cells exposed to the same conditions. HMOX-1 (hemeoxidase-1). MAOA (monoamine oxidase A), ferroportin (FPT). **B**- Representative images of endogenous NRF2 distribution in WT, LRRK2$_{G2019S}$ and LRRK2 KO macrophages treated for 48 hours with FAS 100 μM. Cell nuclei were stained with DAPI, lysosomes were stained with anti LAMP1 antibody. Scale bar 10 μm. **C**- Quantification of the percentage of cells showing nuclear NRF2 translocation. Cells analyzed per biological replicate: WT+FAS N=185, 214, 183, LRRK2$_{G2019S}$+ FAS N=165, 182, 189. The means ± SD and single values of three biological replicates are shown. T-test statistical analysis is shown. **D**- Growth curve of WT, LRRK2$_{G2019S}$ and LRRK2 KO macrophages. Cell count was followed for 3 days in the presence or absence of 100 μM FAS. Each point shows the average cell number ± SD of 4 technical replicates.

Figure 1A

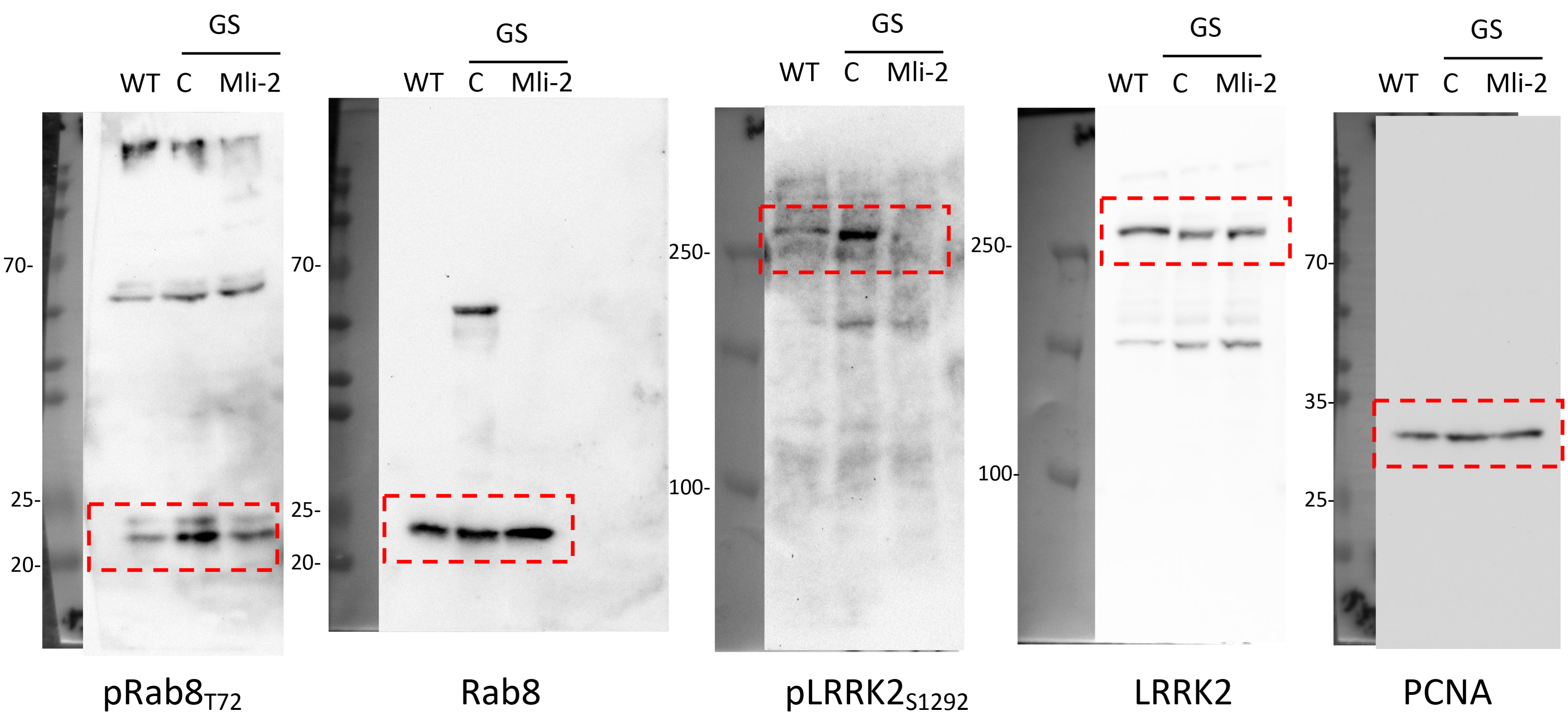

Figure 1B

Densitometry triplicates

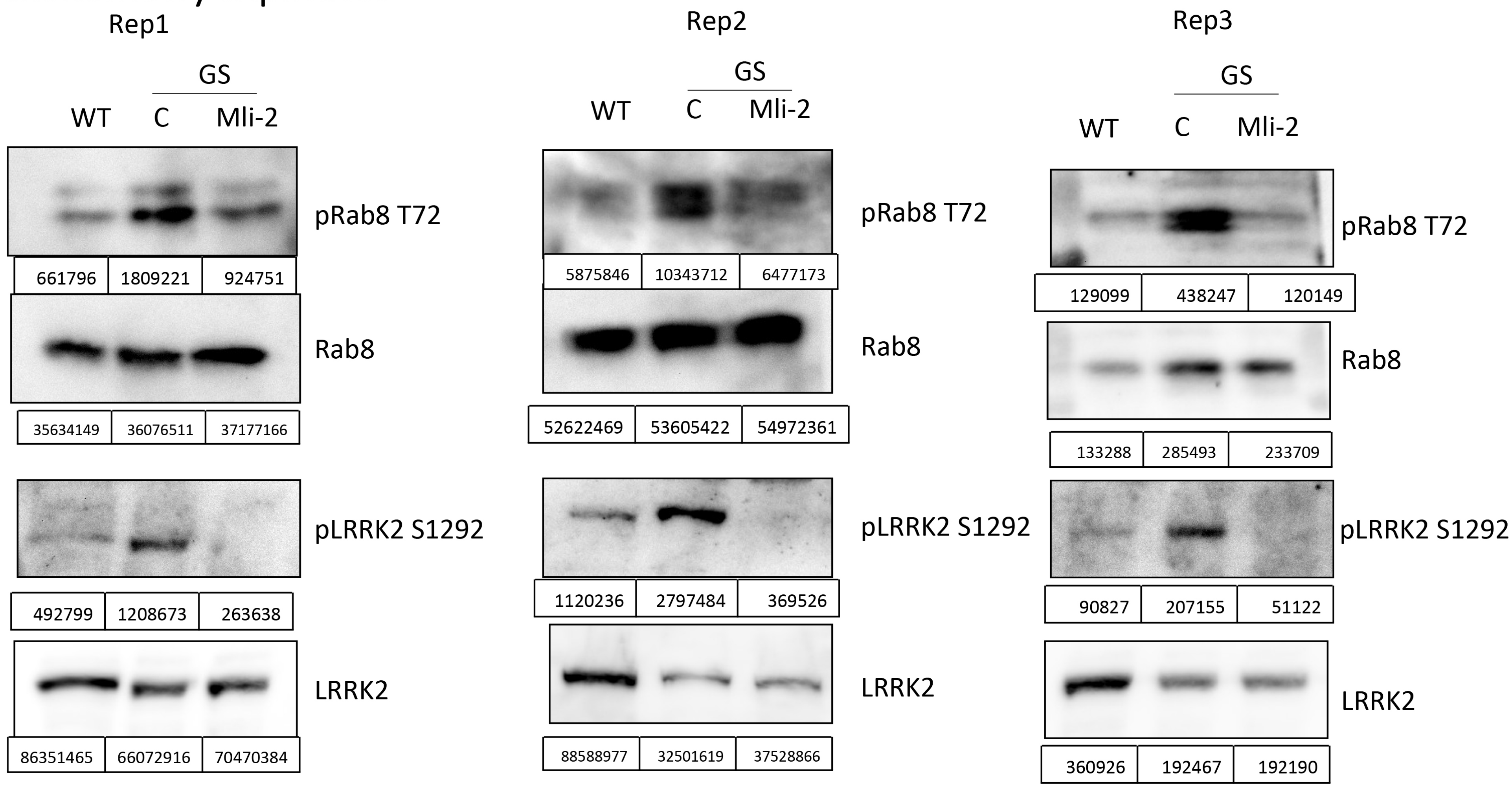

Journal of Cell Science • Supplementary information

Figure 1E

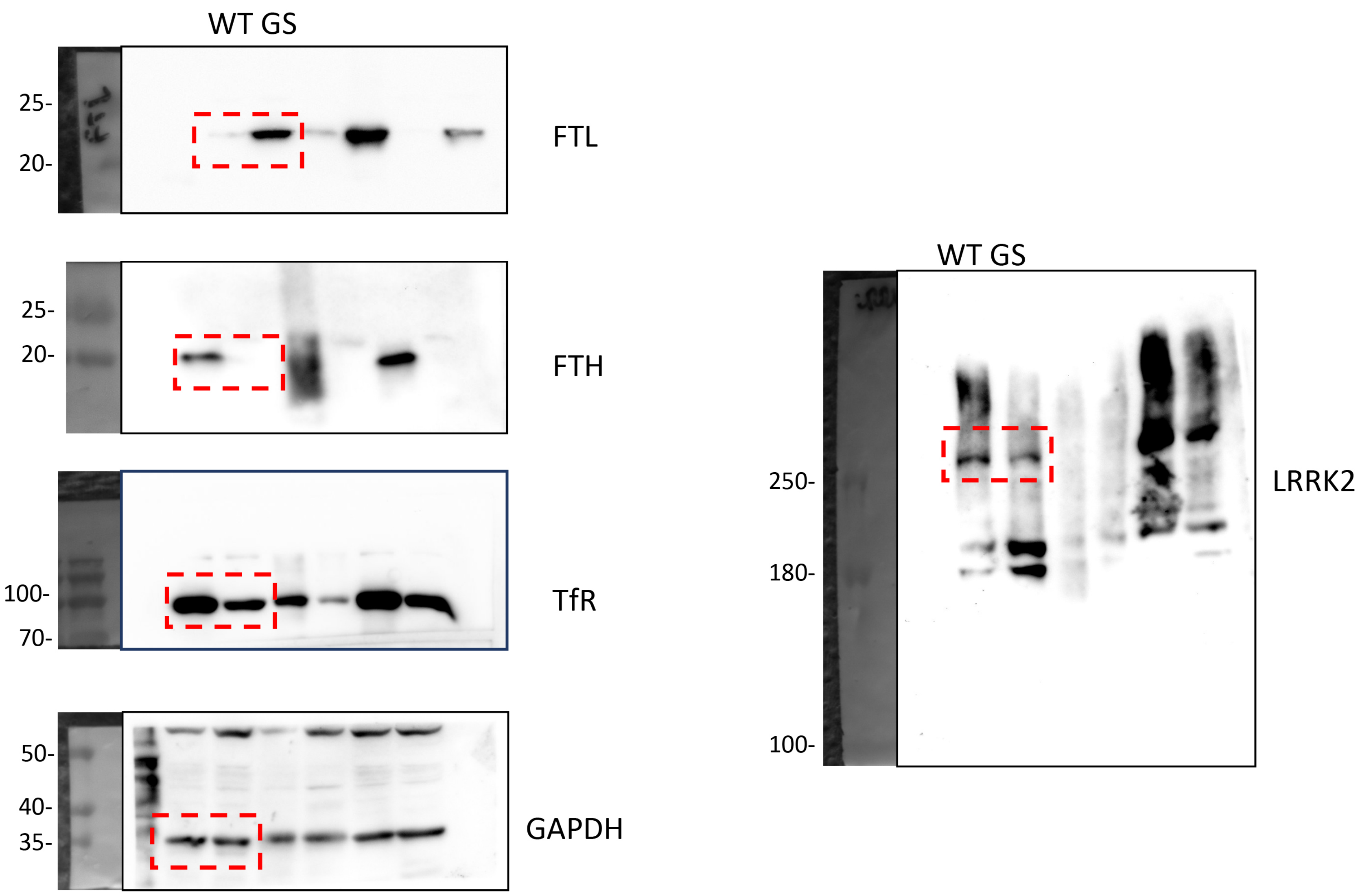

Figure 1I

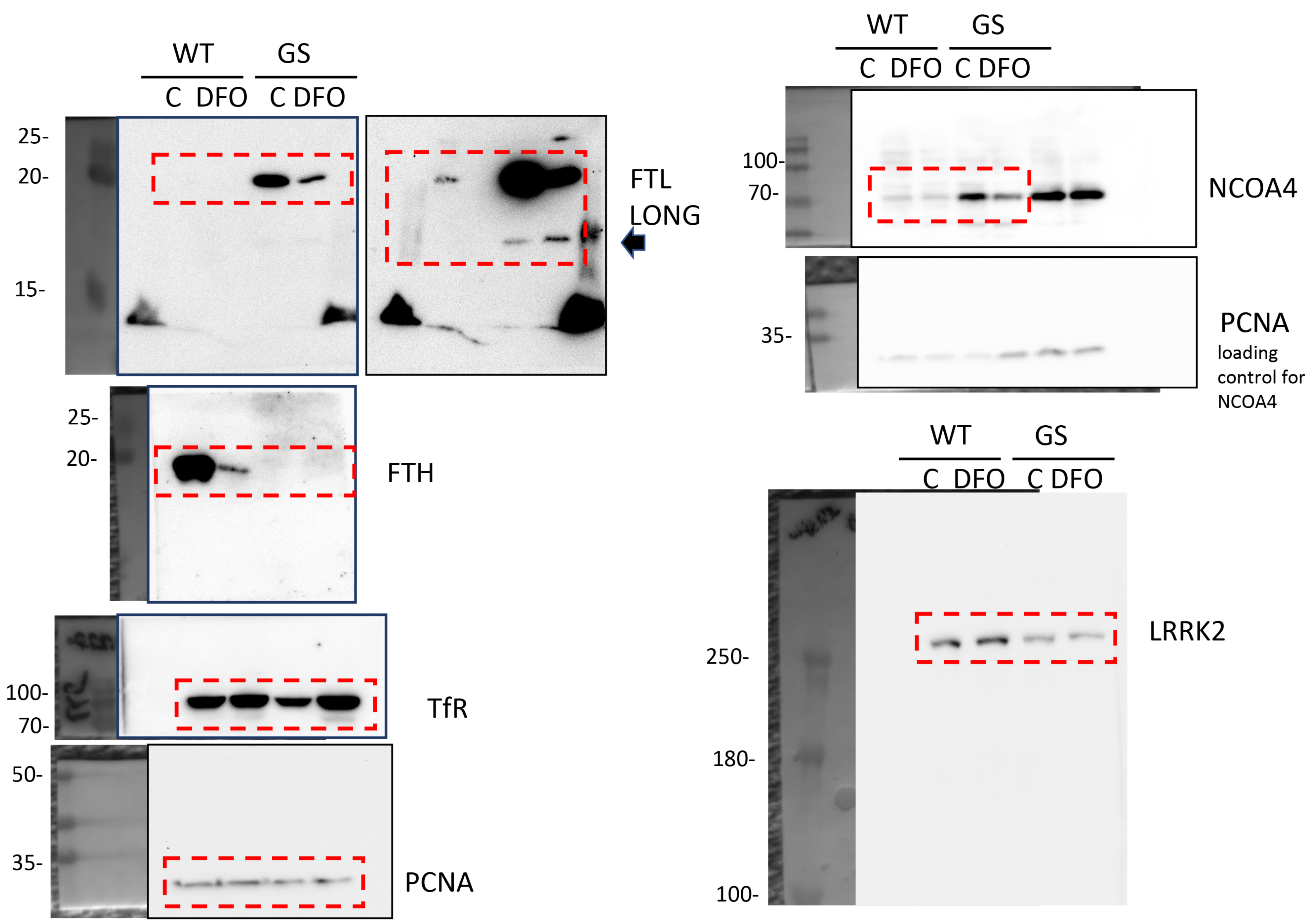

Journal of Cell Science • Supplementary information

Supplmentary Figure 1B

Densitometry triplicates

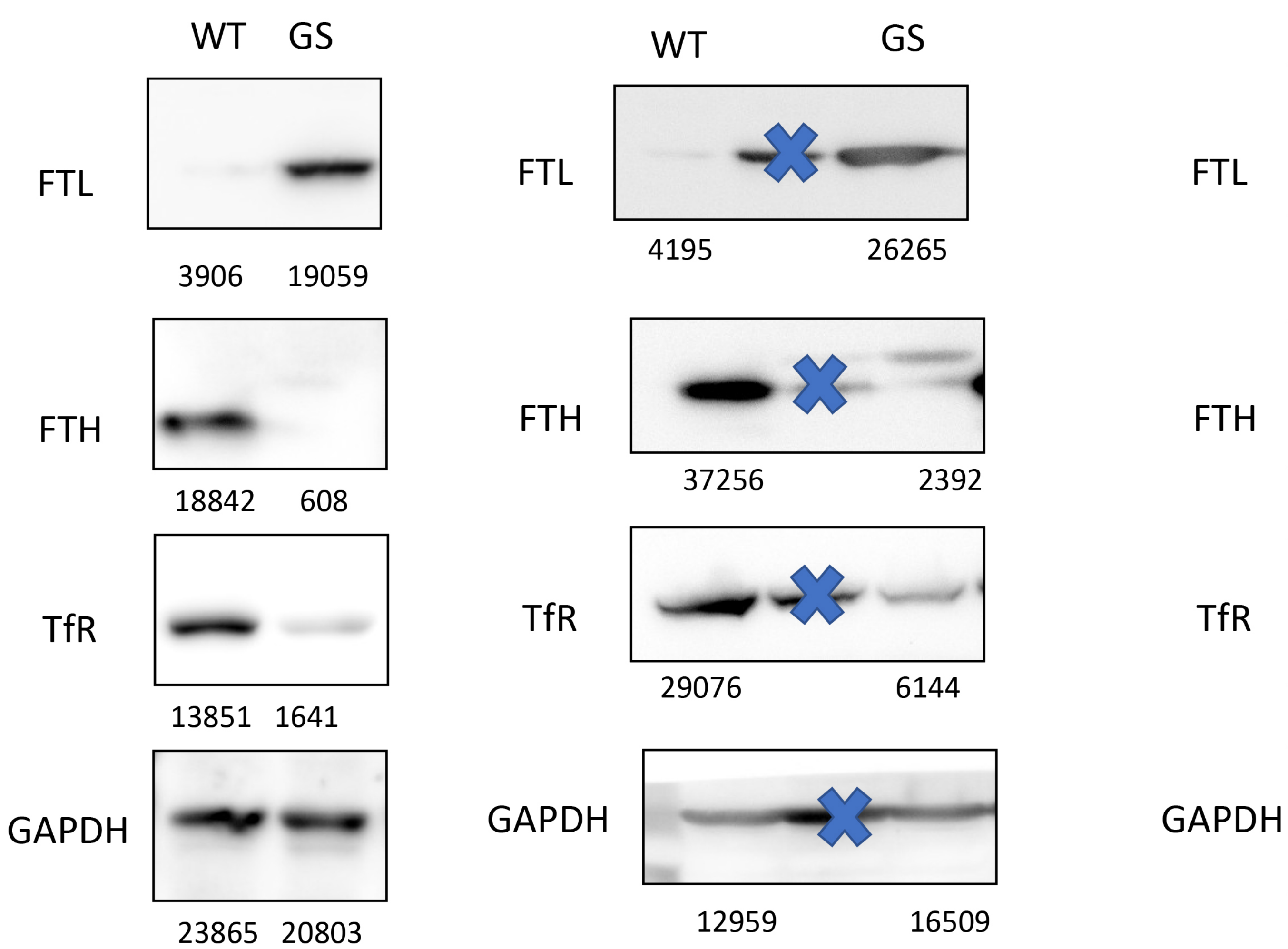

Supplementary Figure 1D

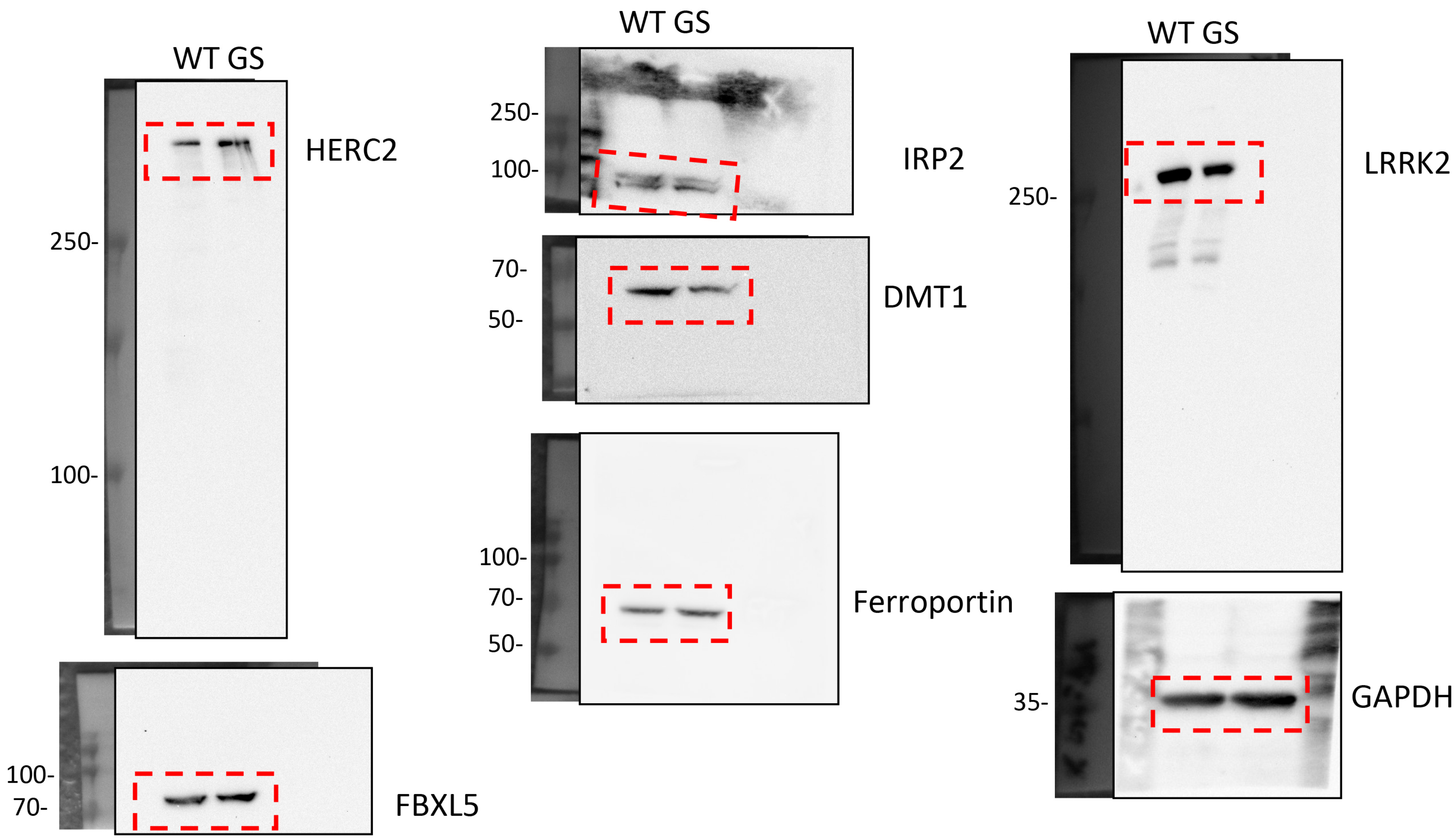

Supplementary Figure 1G

Densitometry triplicates

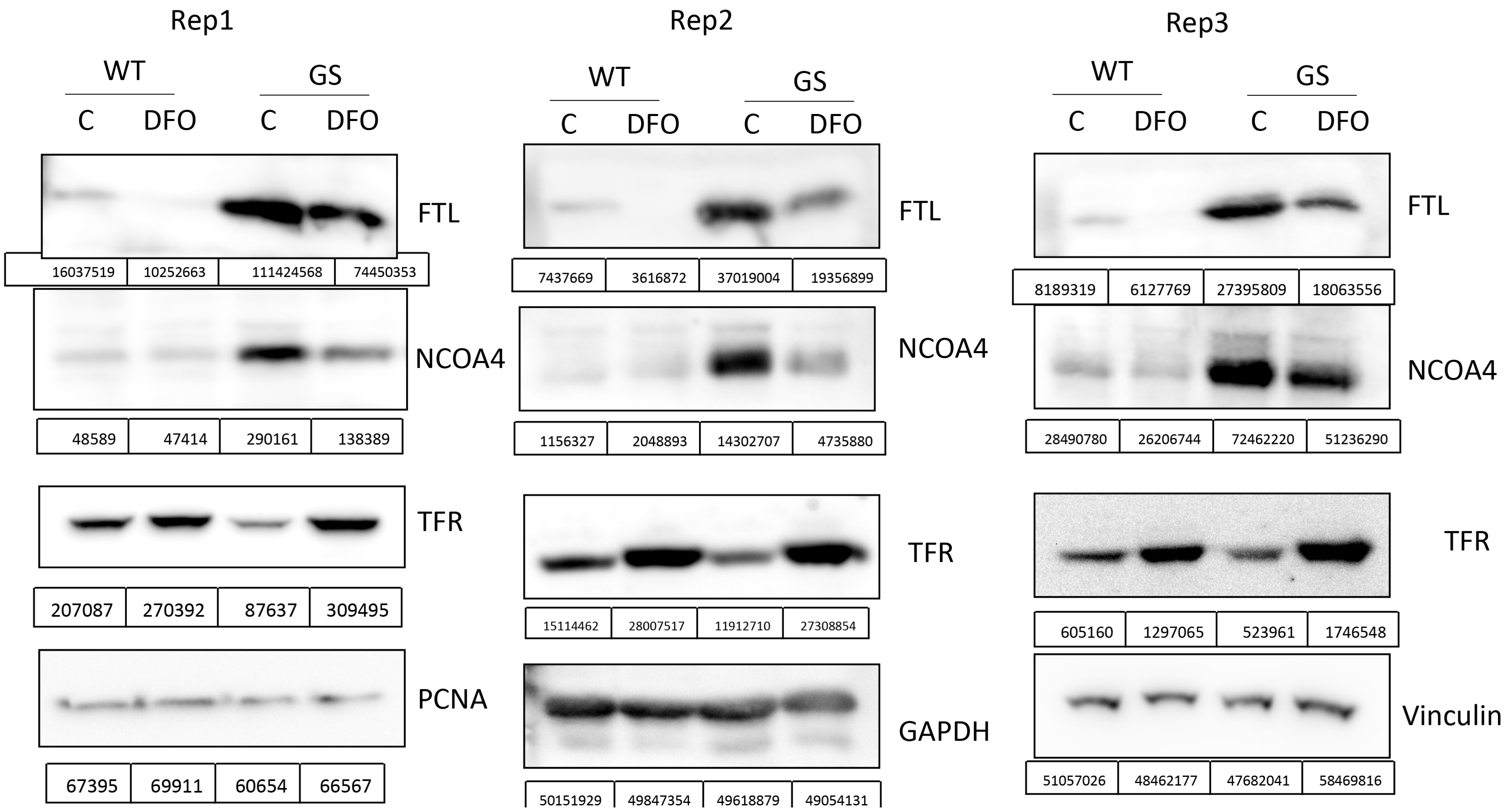

Figure 2E

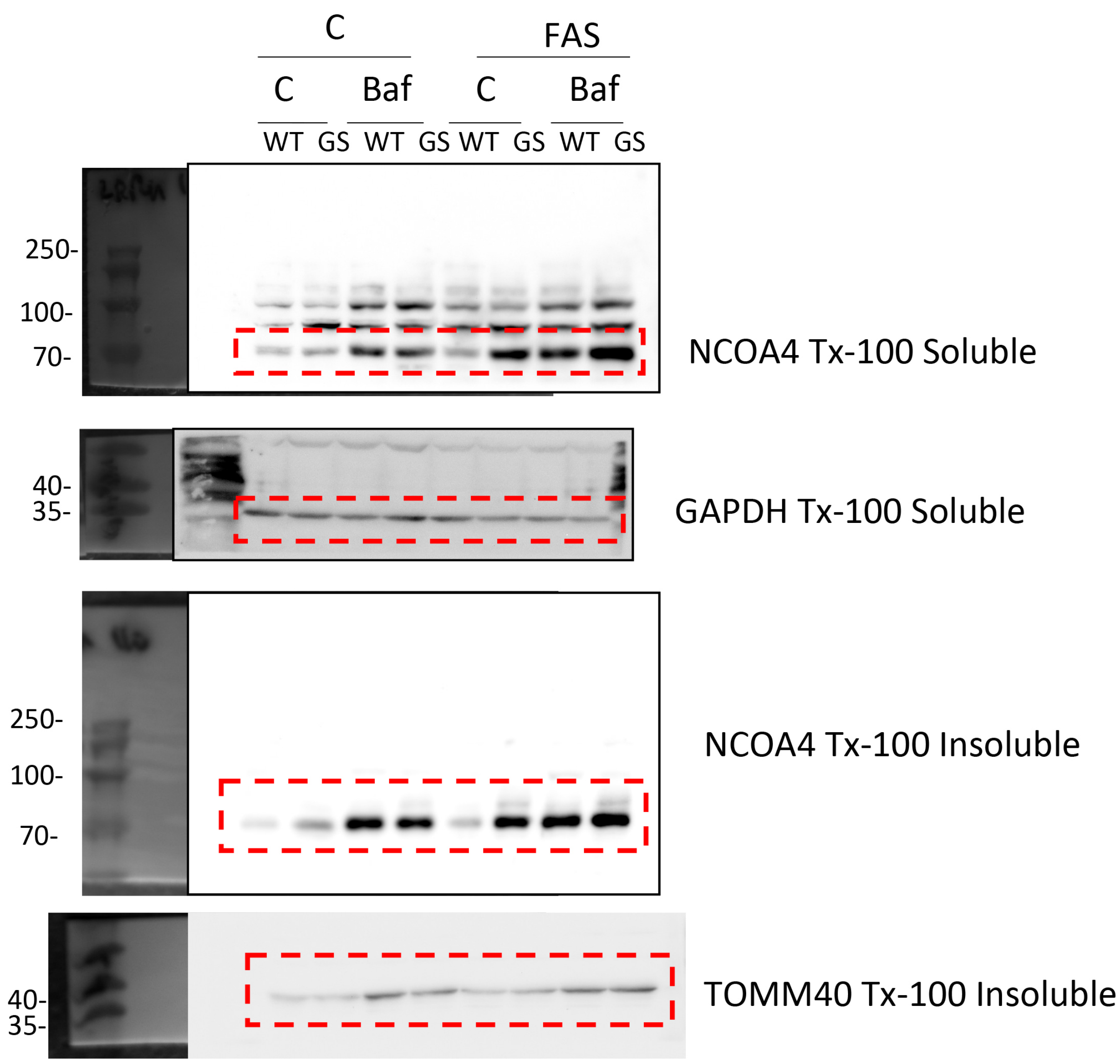

Figure 2F

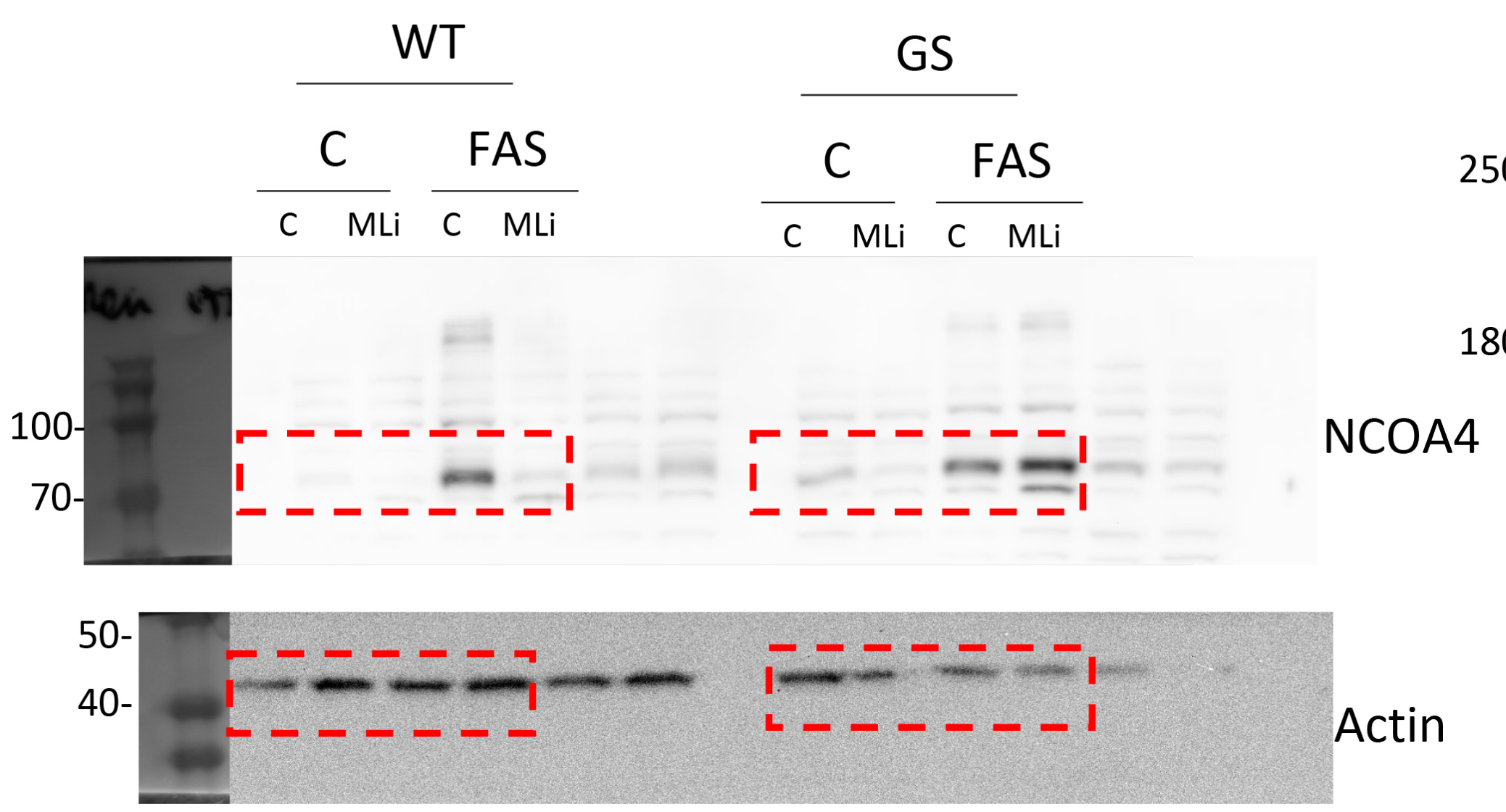

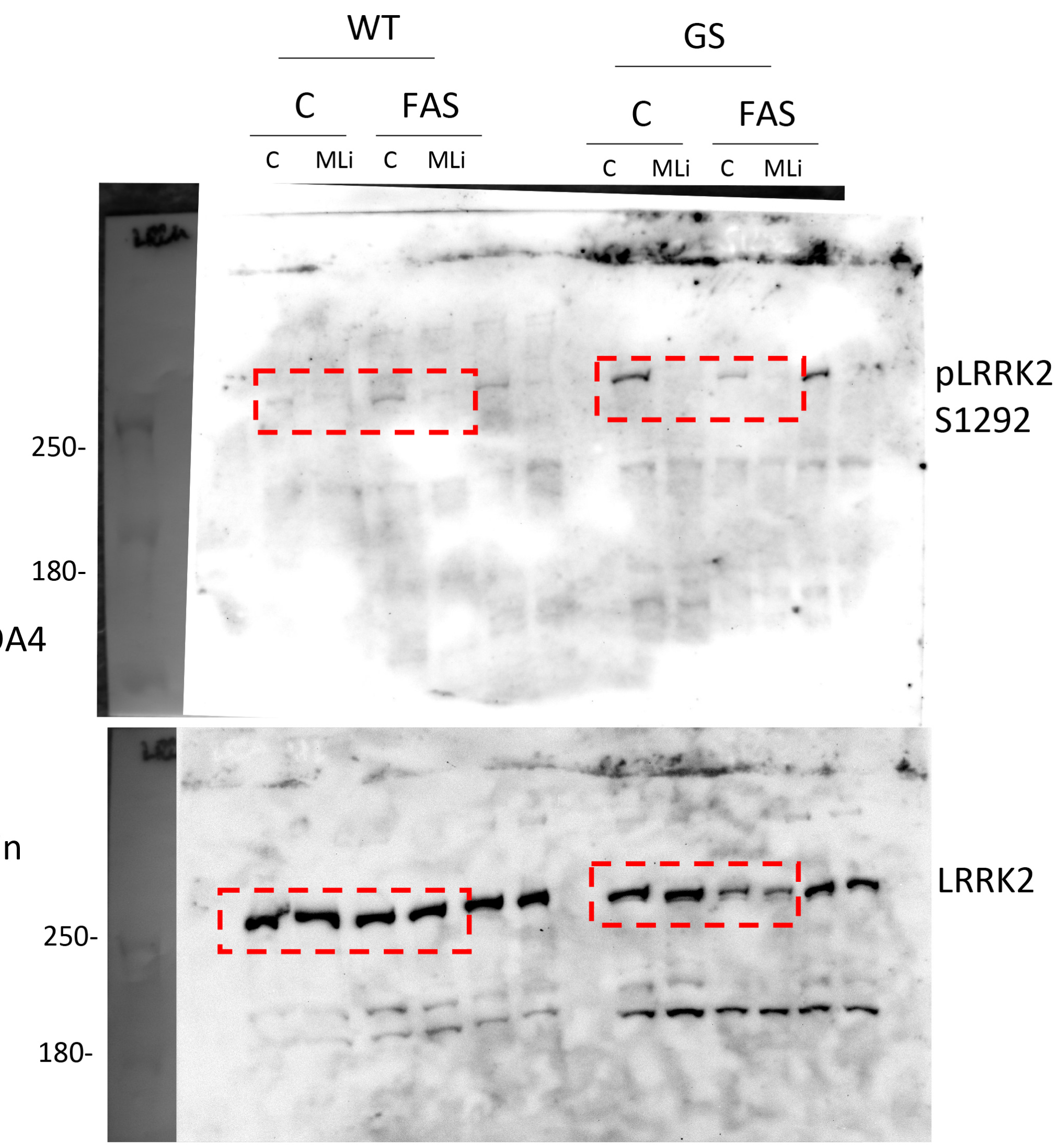

Supplementary figure 2F

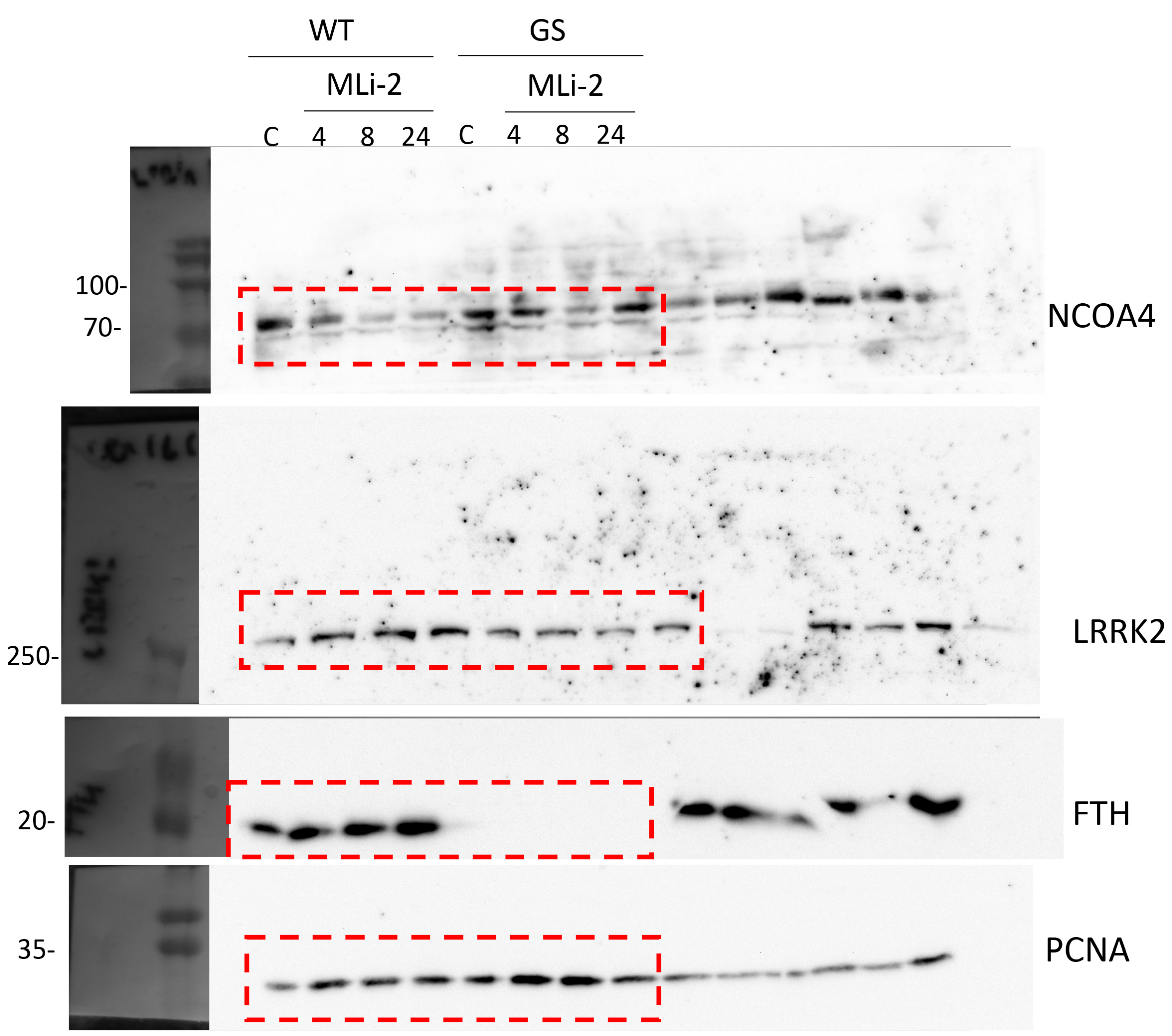

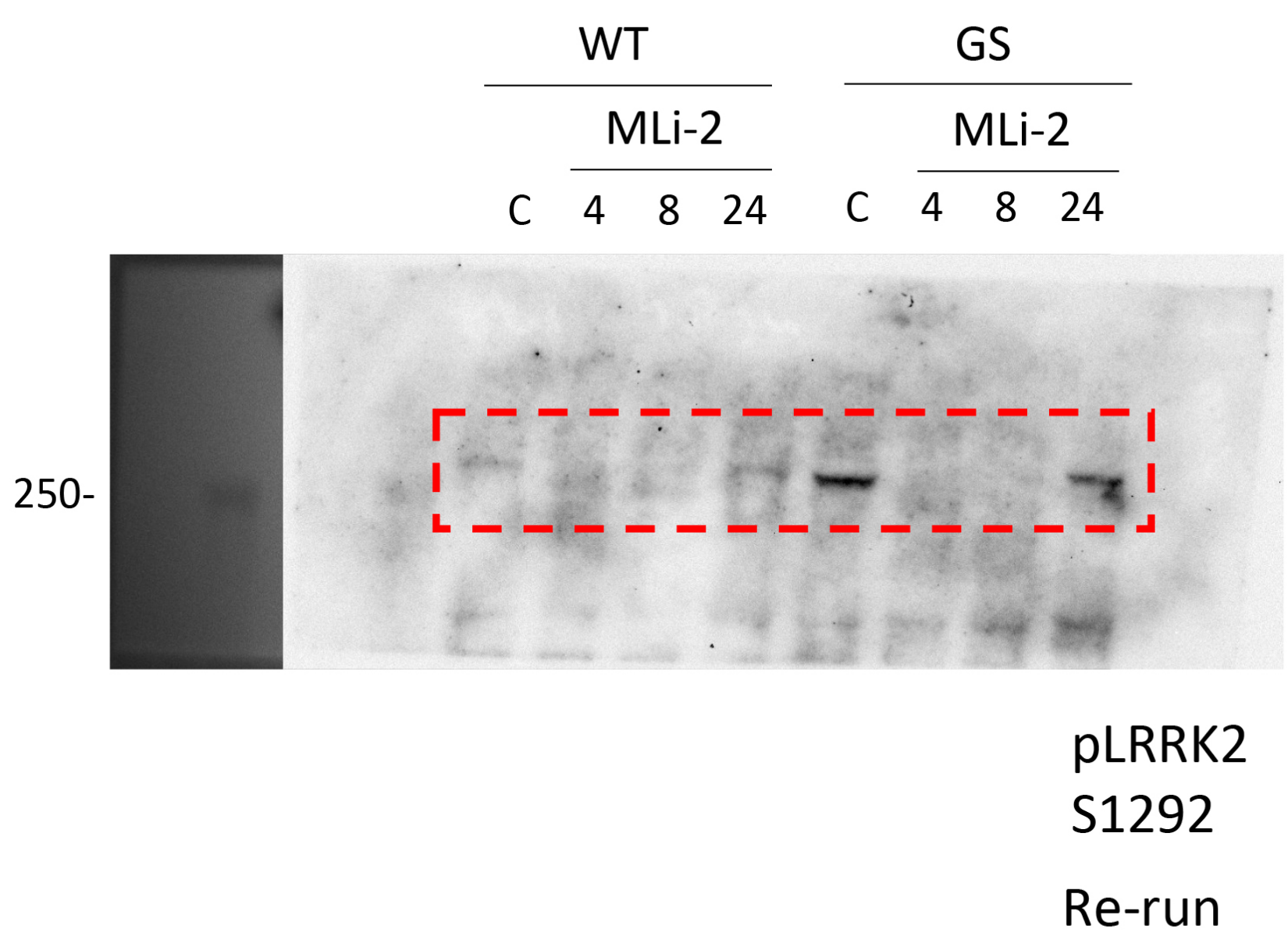

Journal of Cell Science • Supplementary information

Supplementary Figure 2G

Densitometry triplicates

Rep1

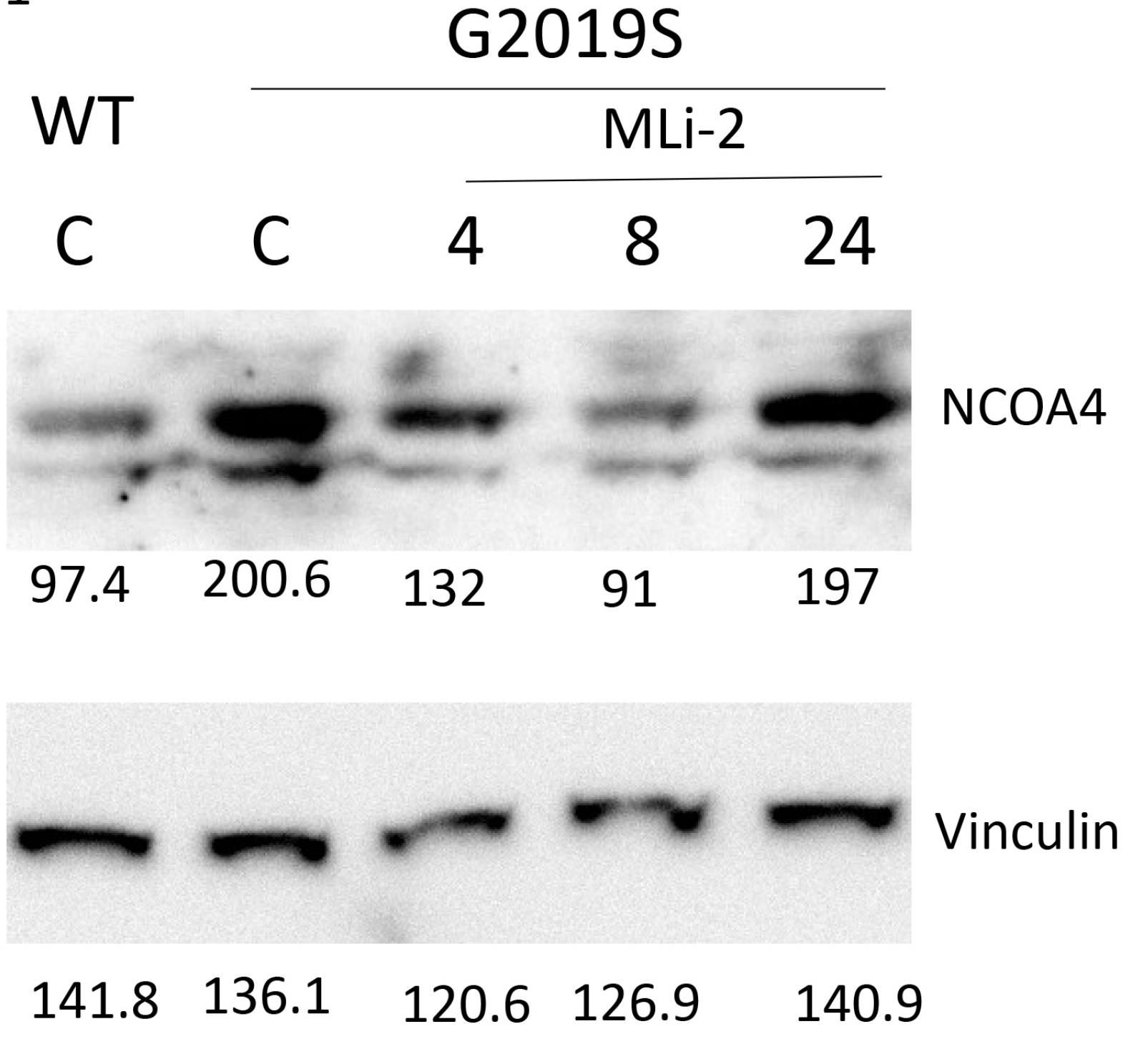

Rep2

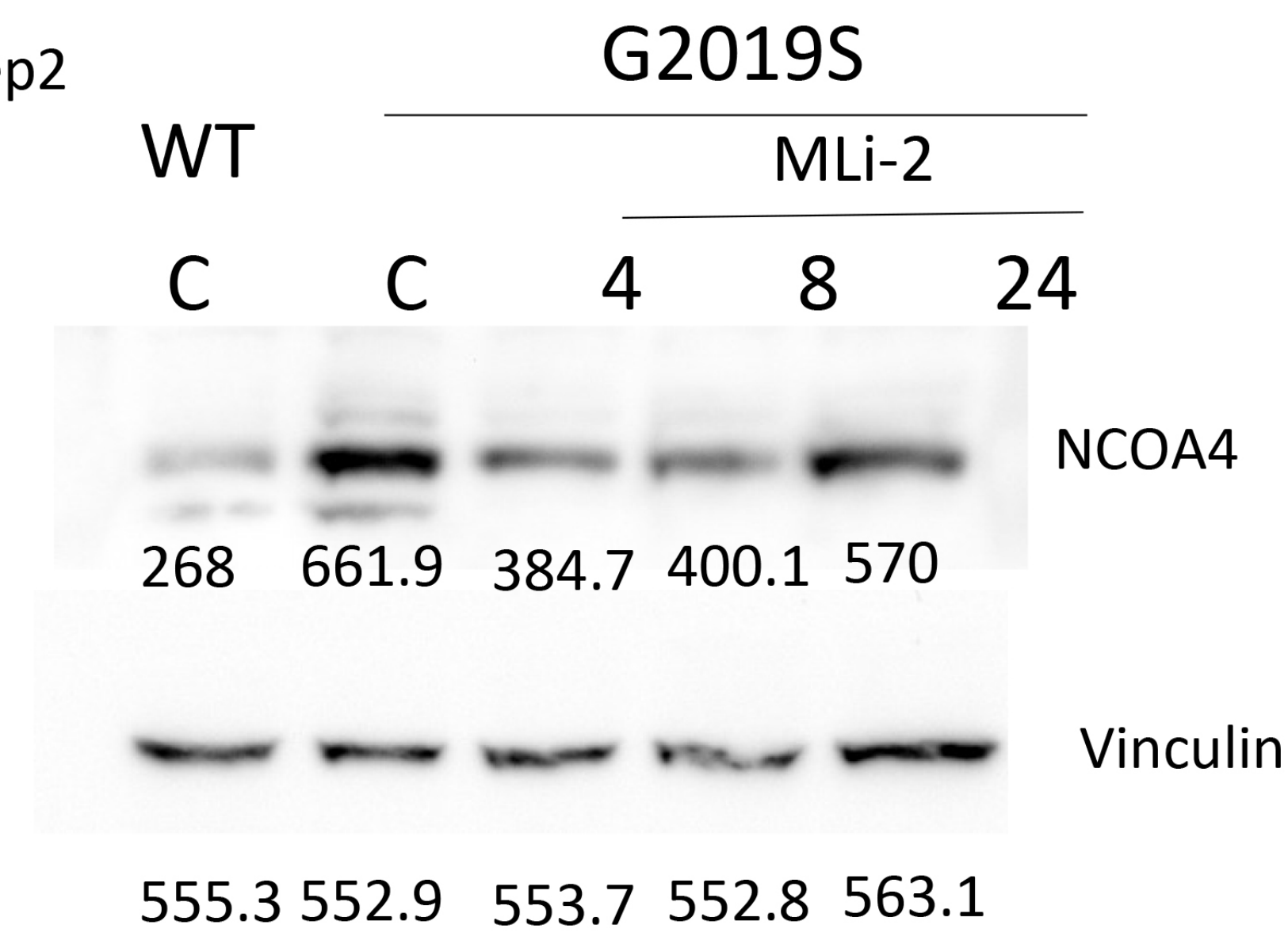

Rep3

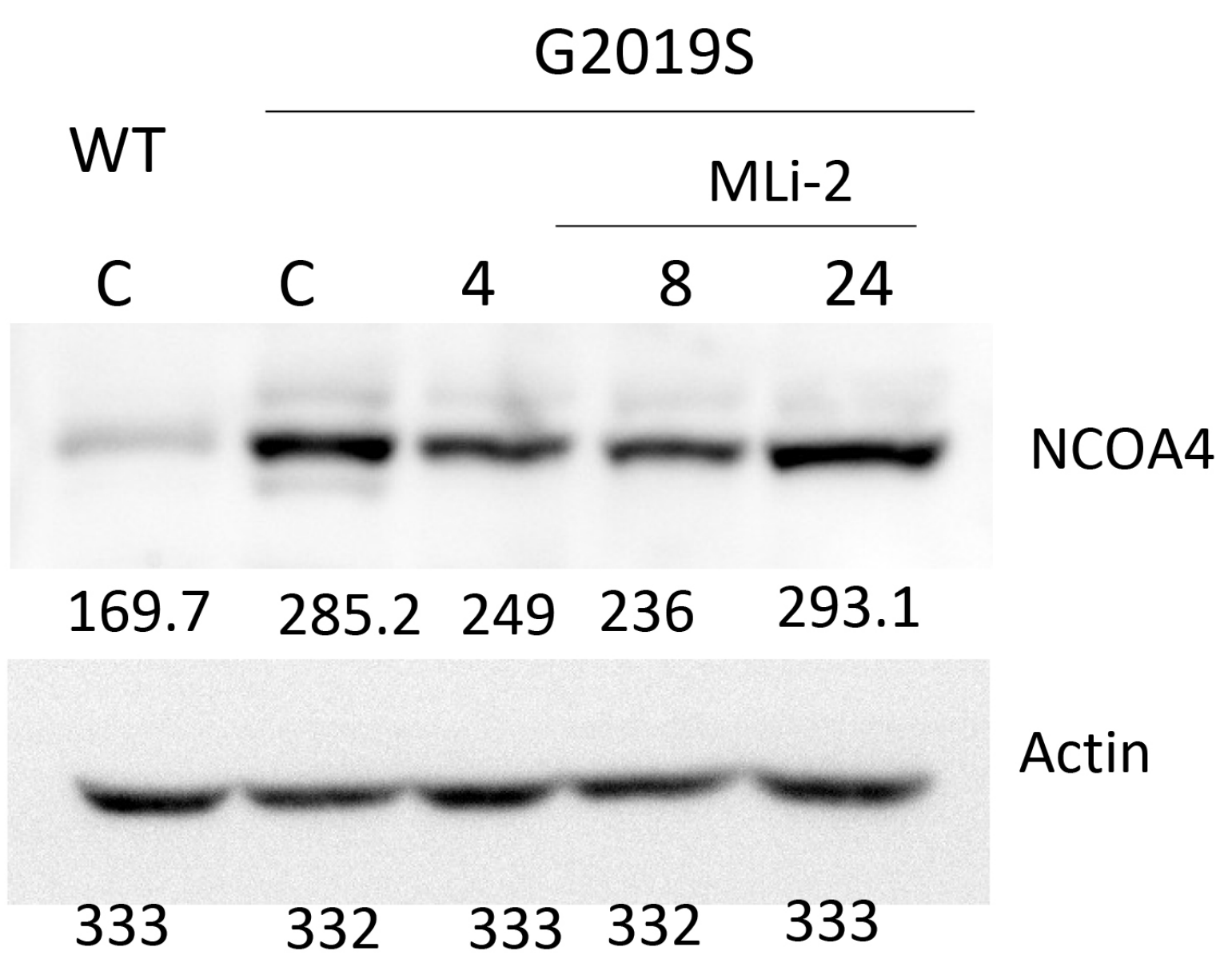

Figure 3C

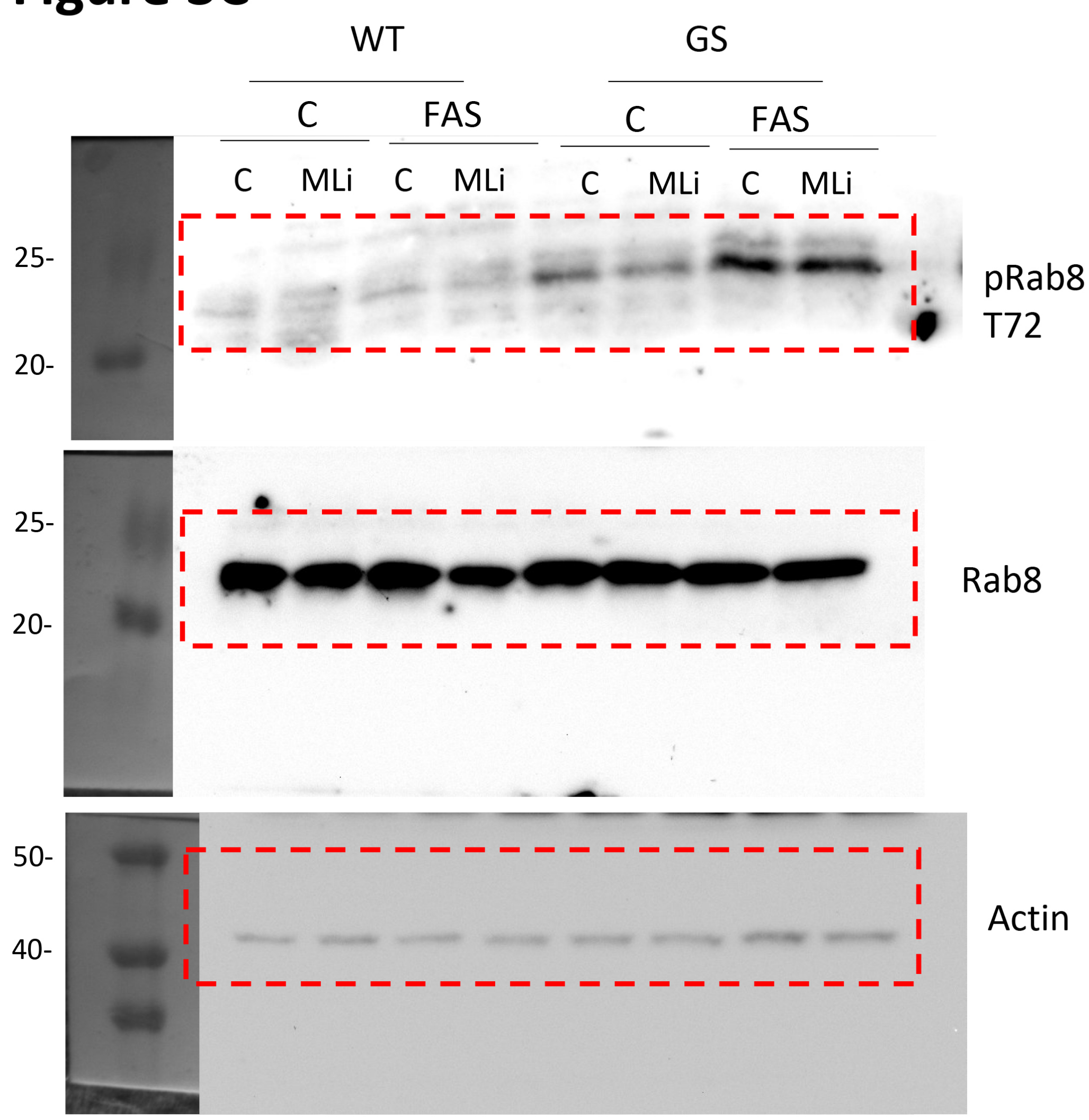

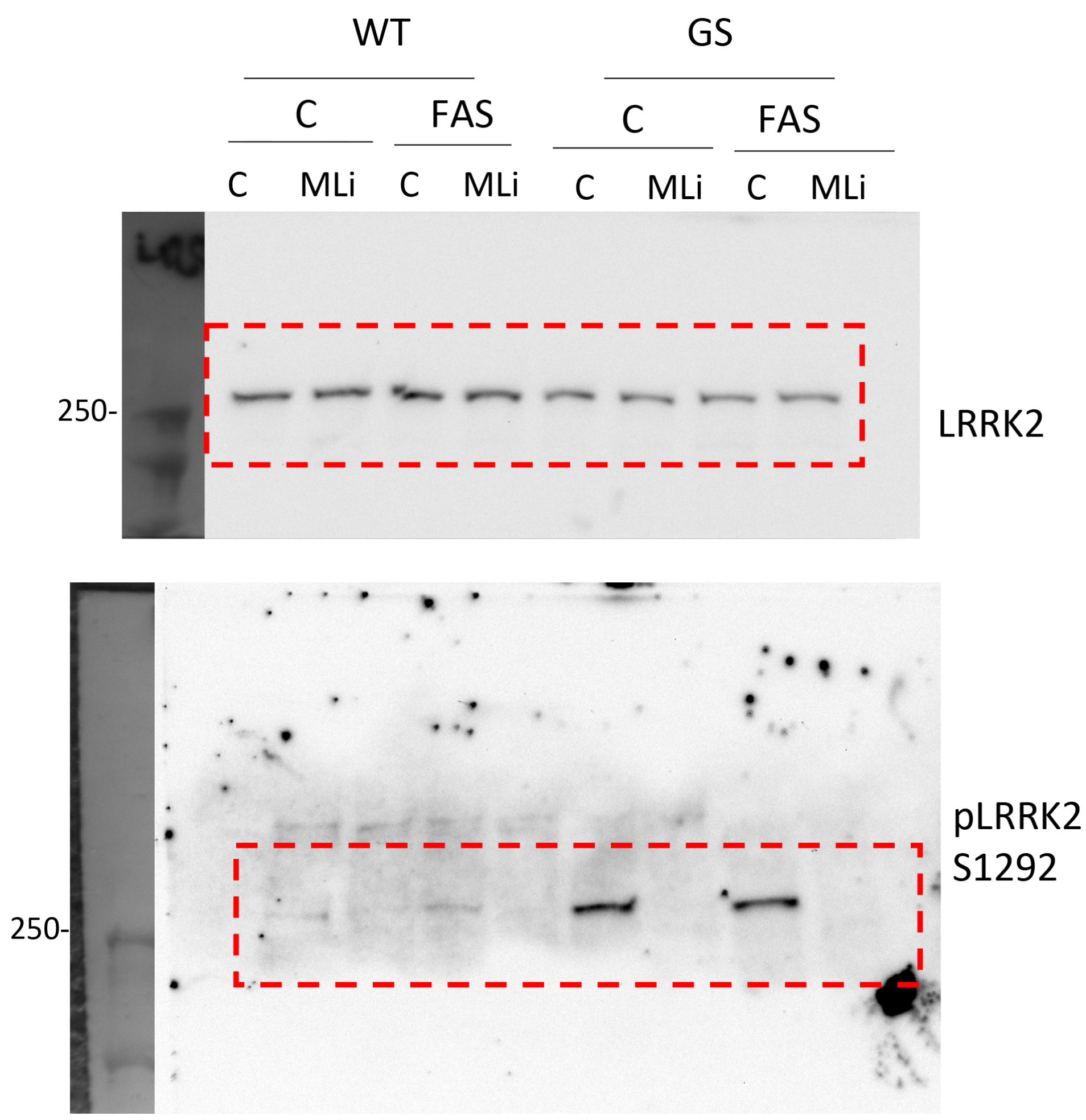

Journal of Cell Science • Supplementary information

Figure 3D

Densitometry triplicates

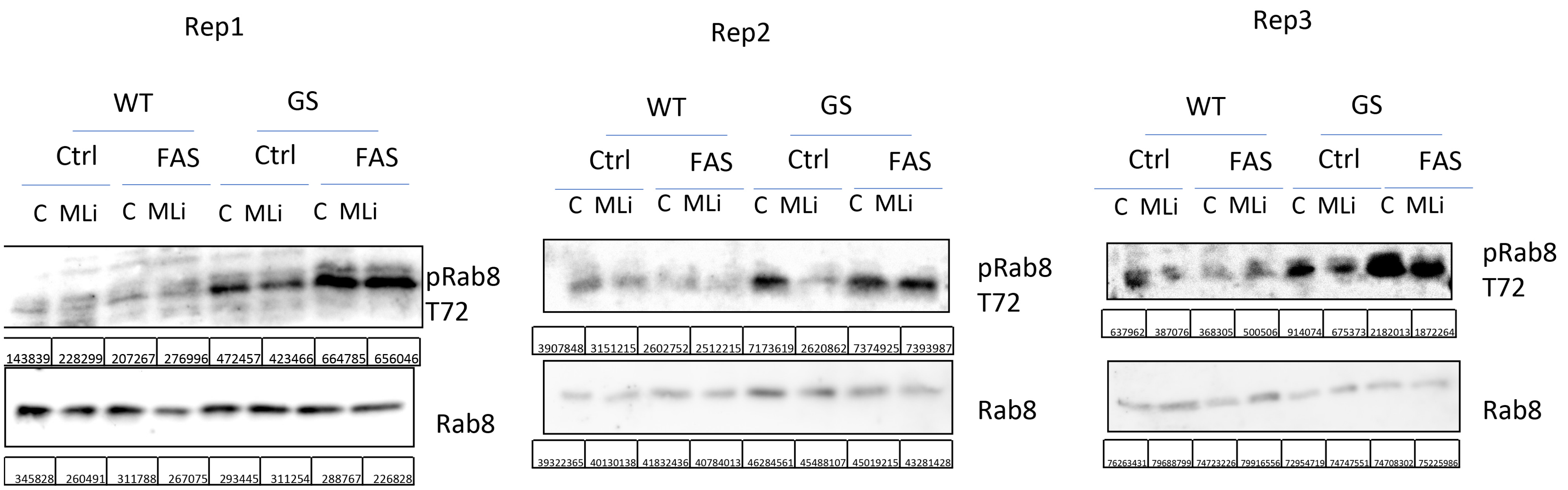

Figure 3E

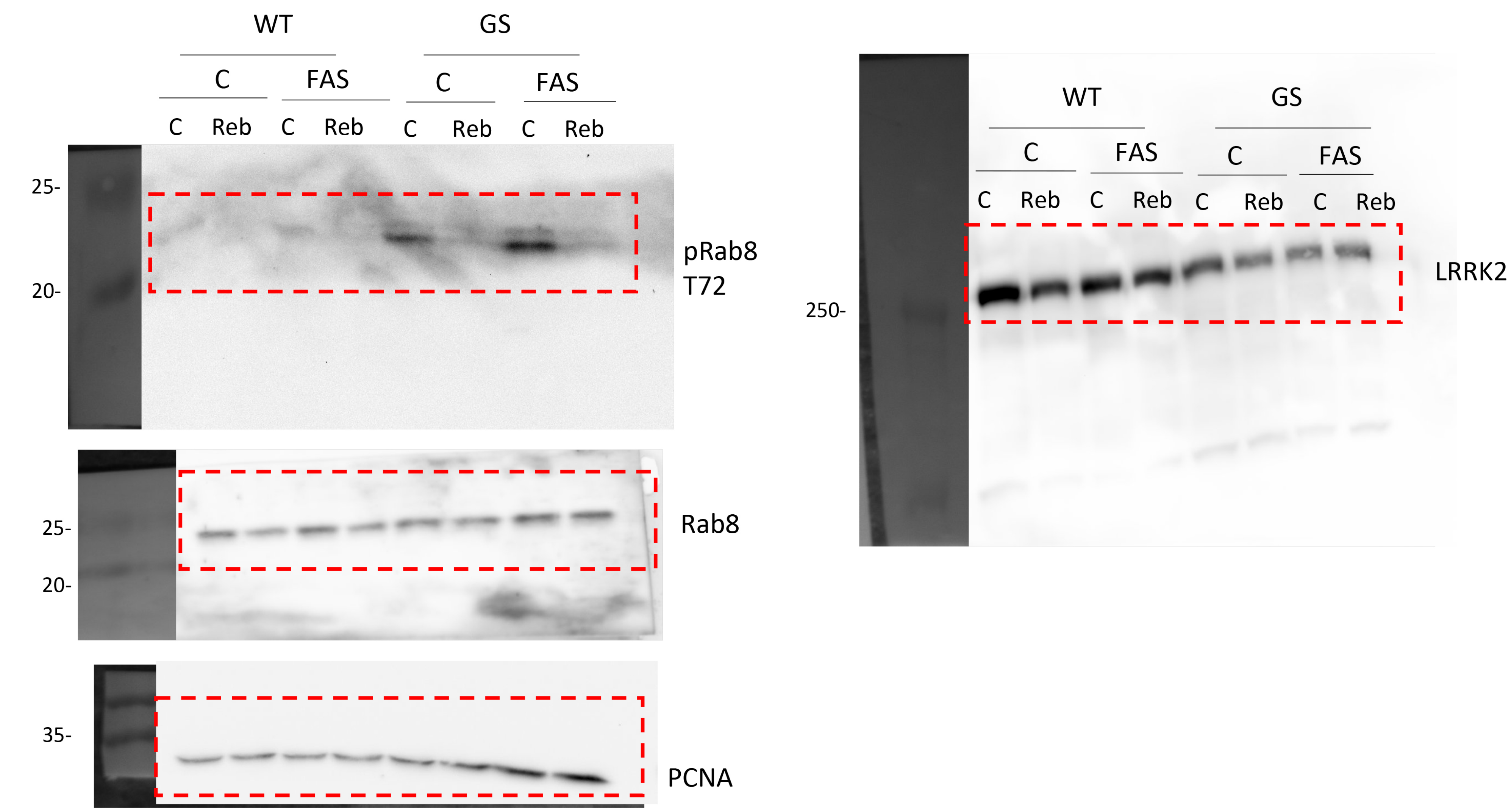

Journal of Cell Science • Supplementary information

Figure 3J

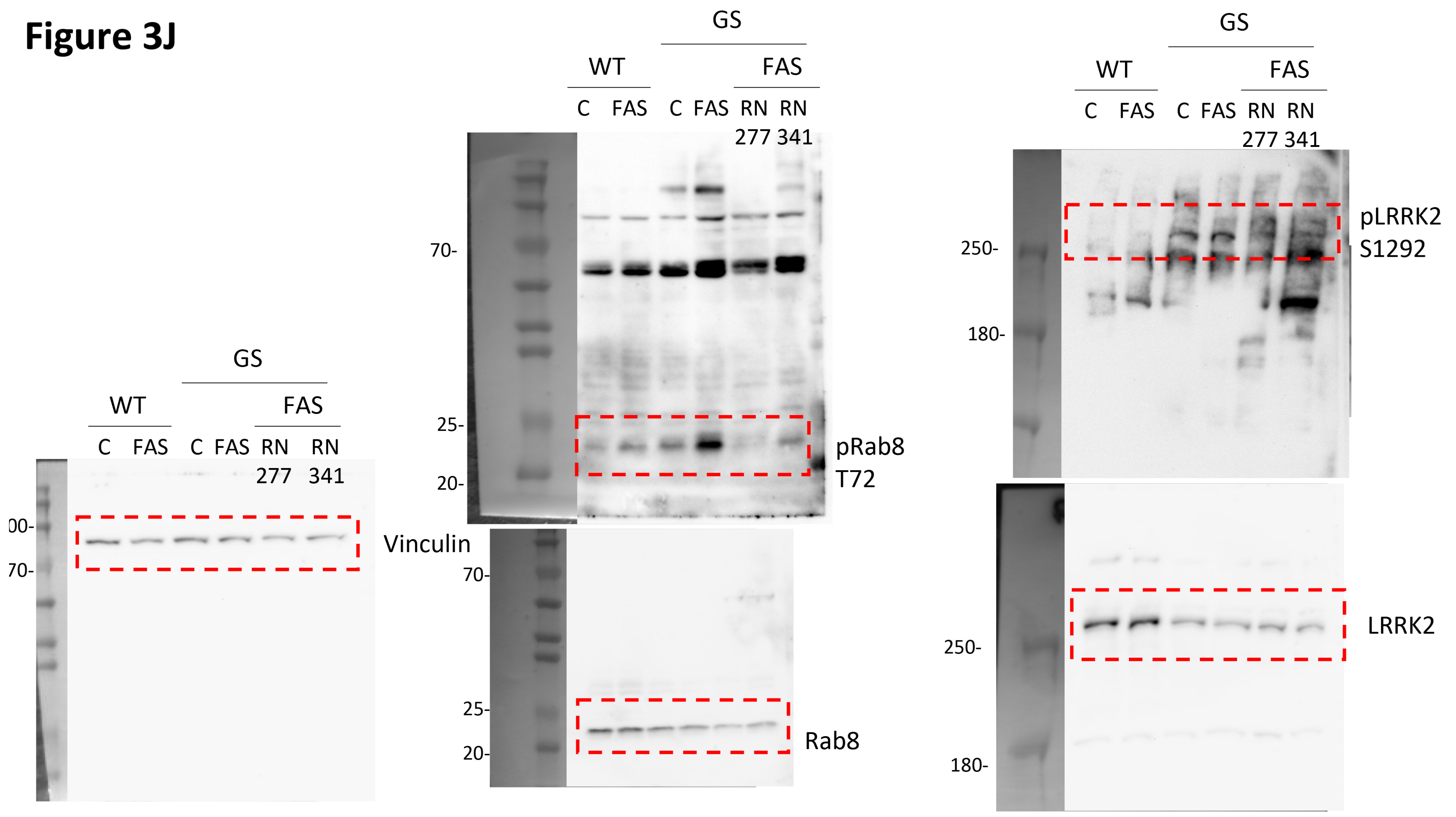

Figure 3K

Densitometry triplicates

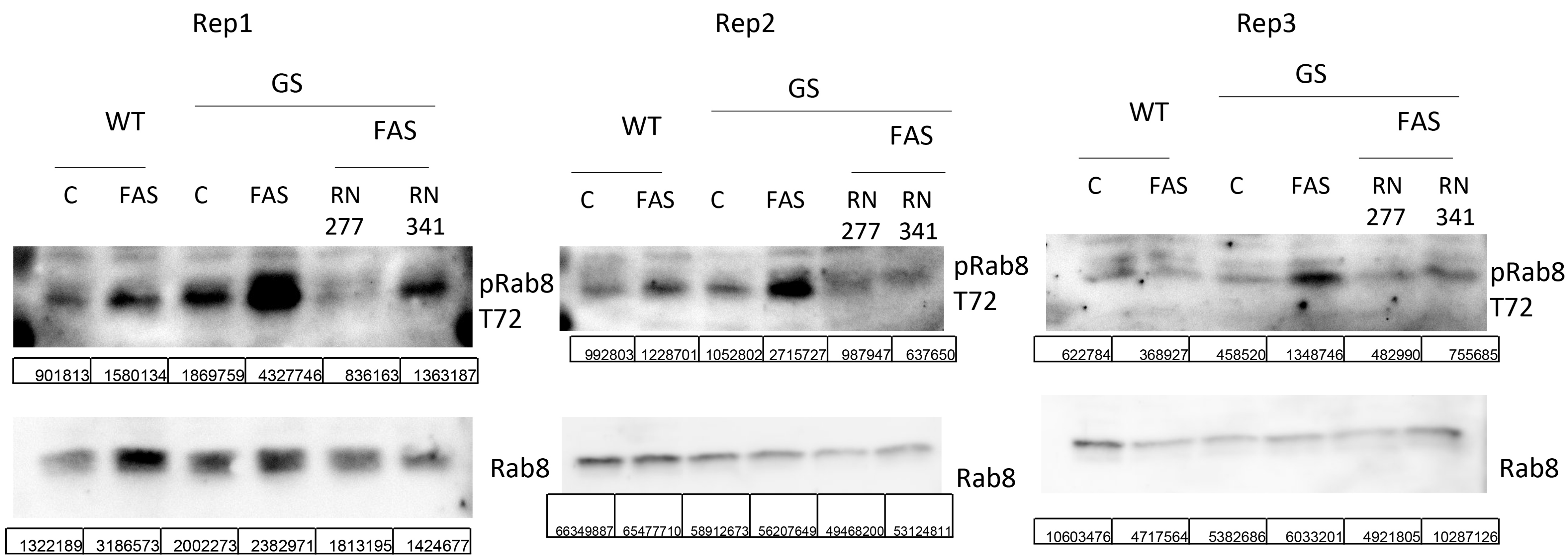

Journal of Cell Science • Supplementary information

Supplementary Figure 3A

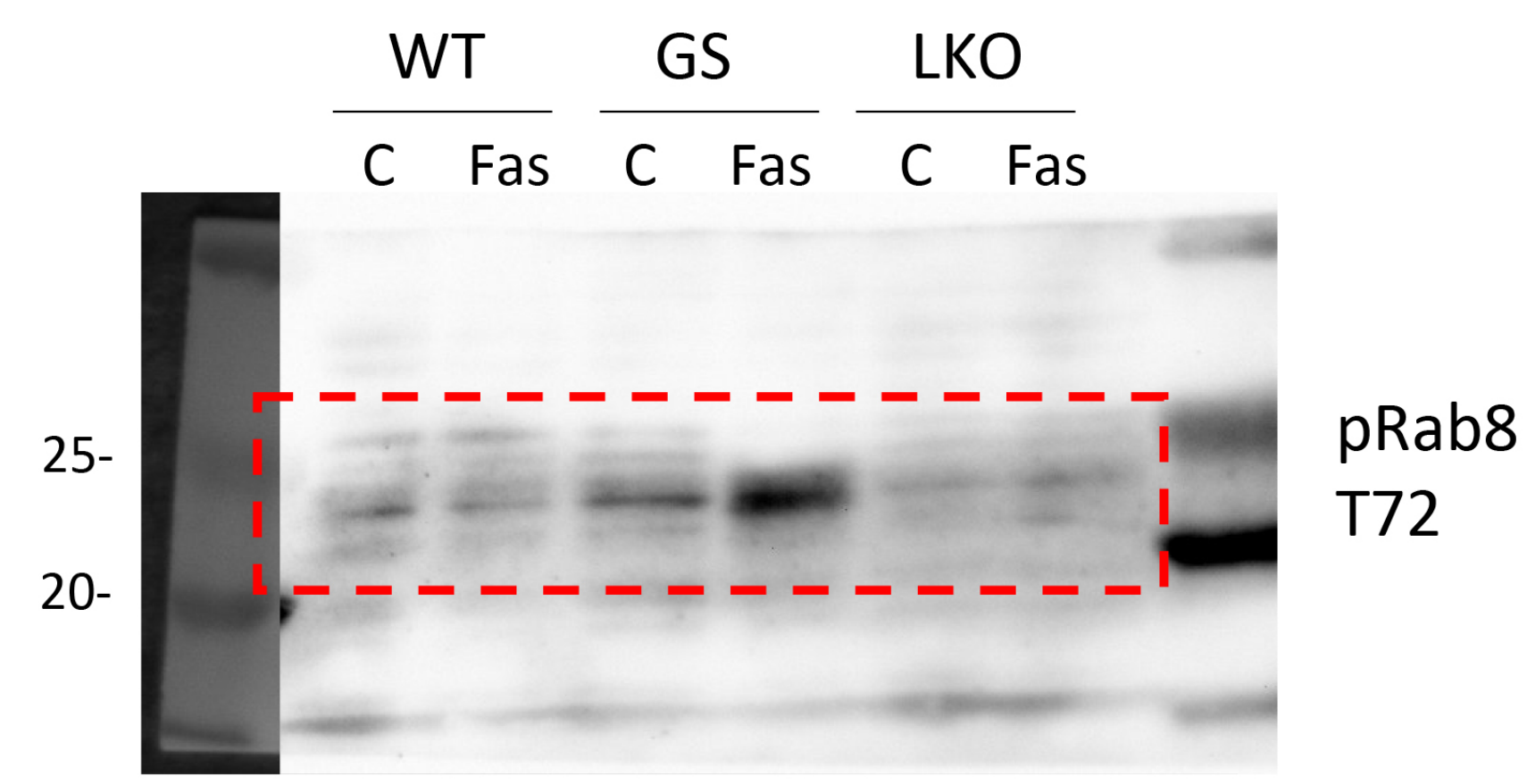

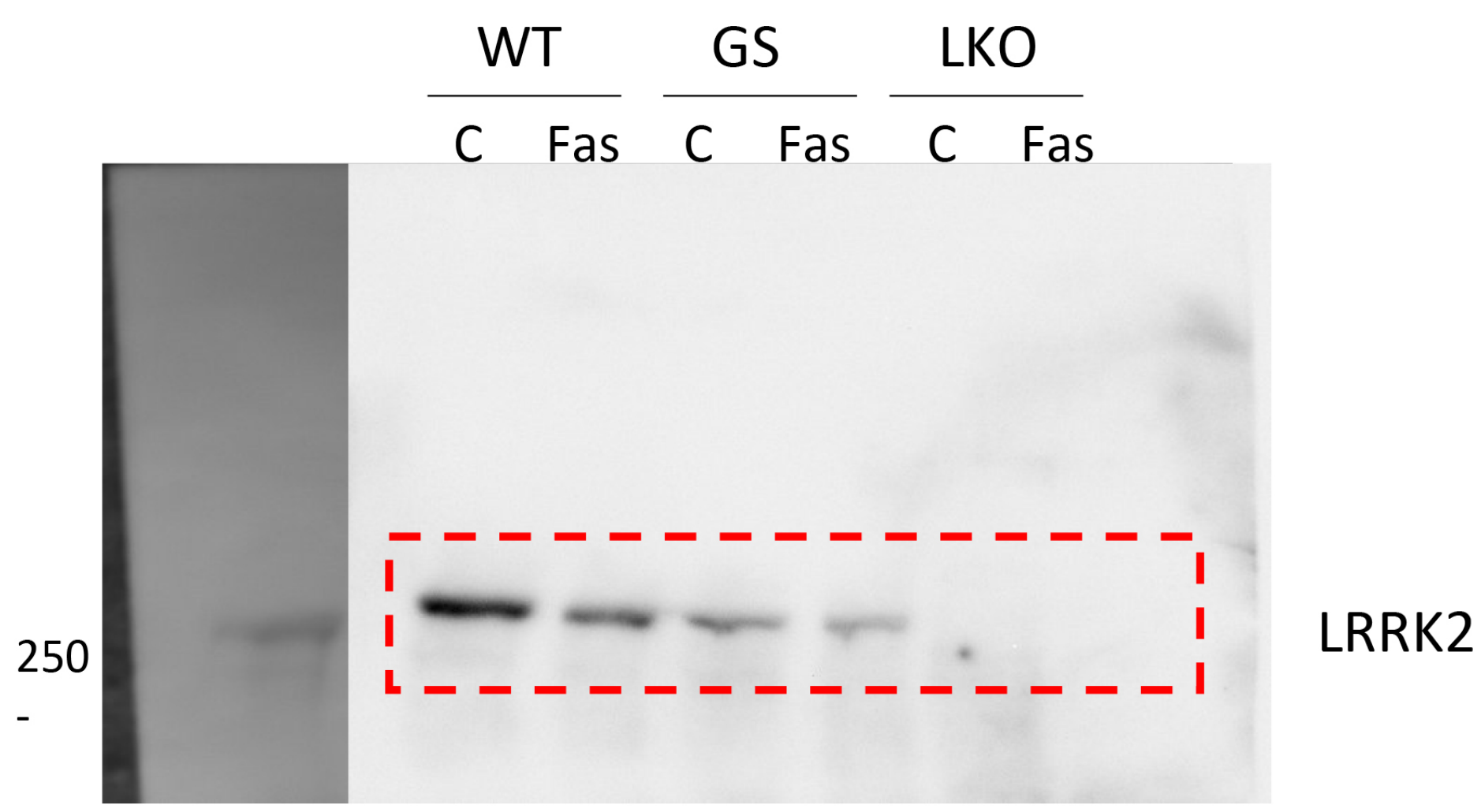

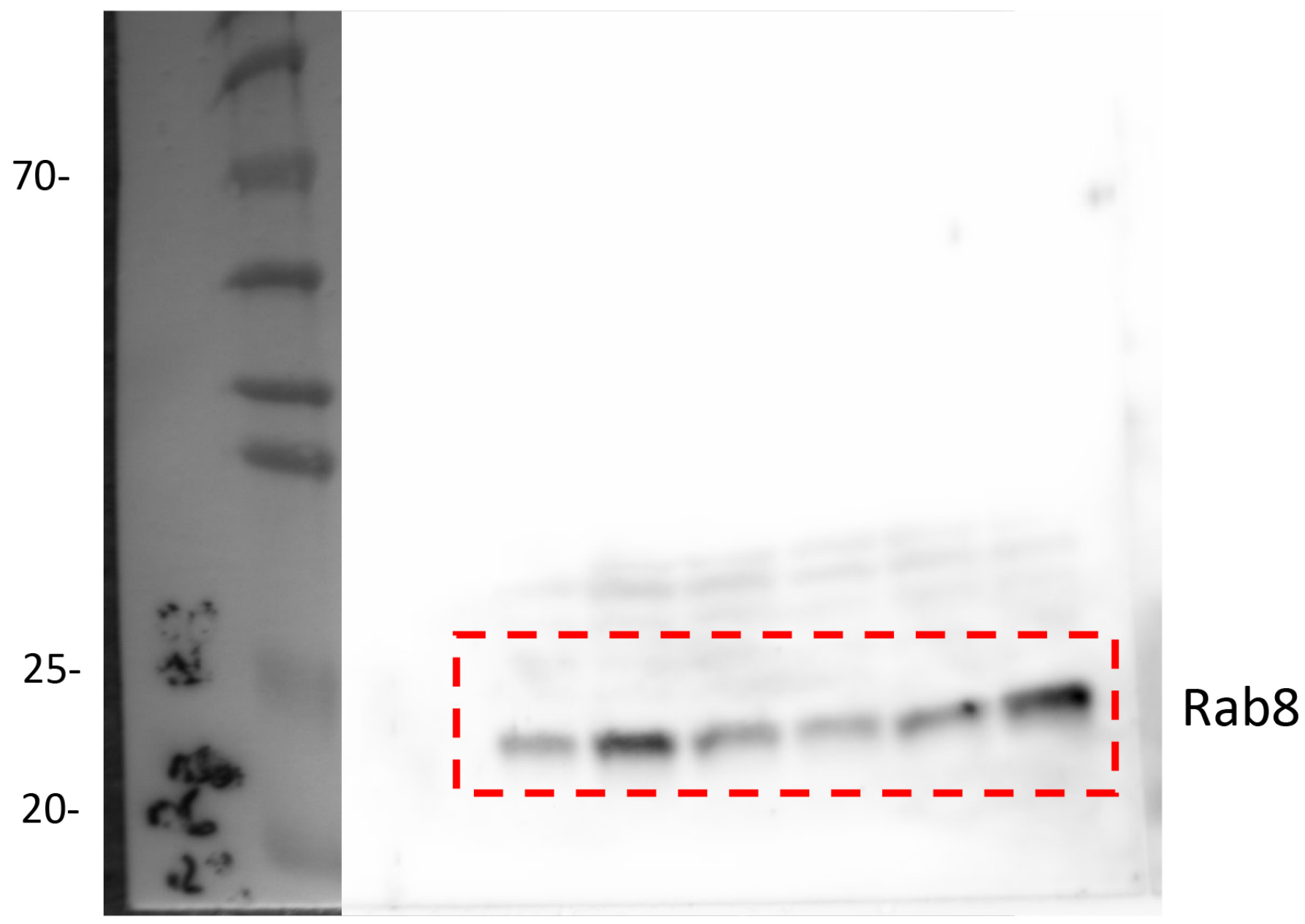

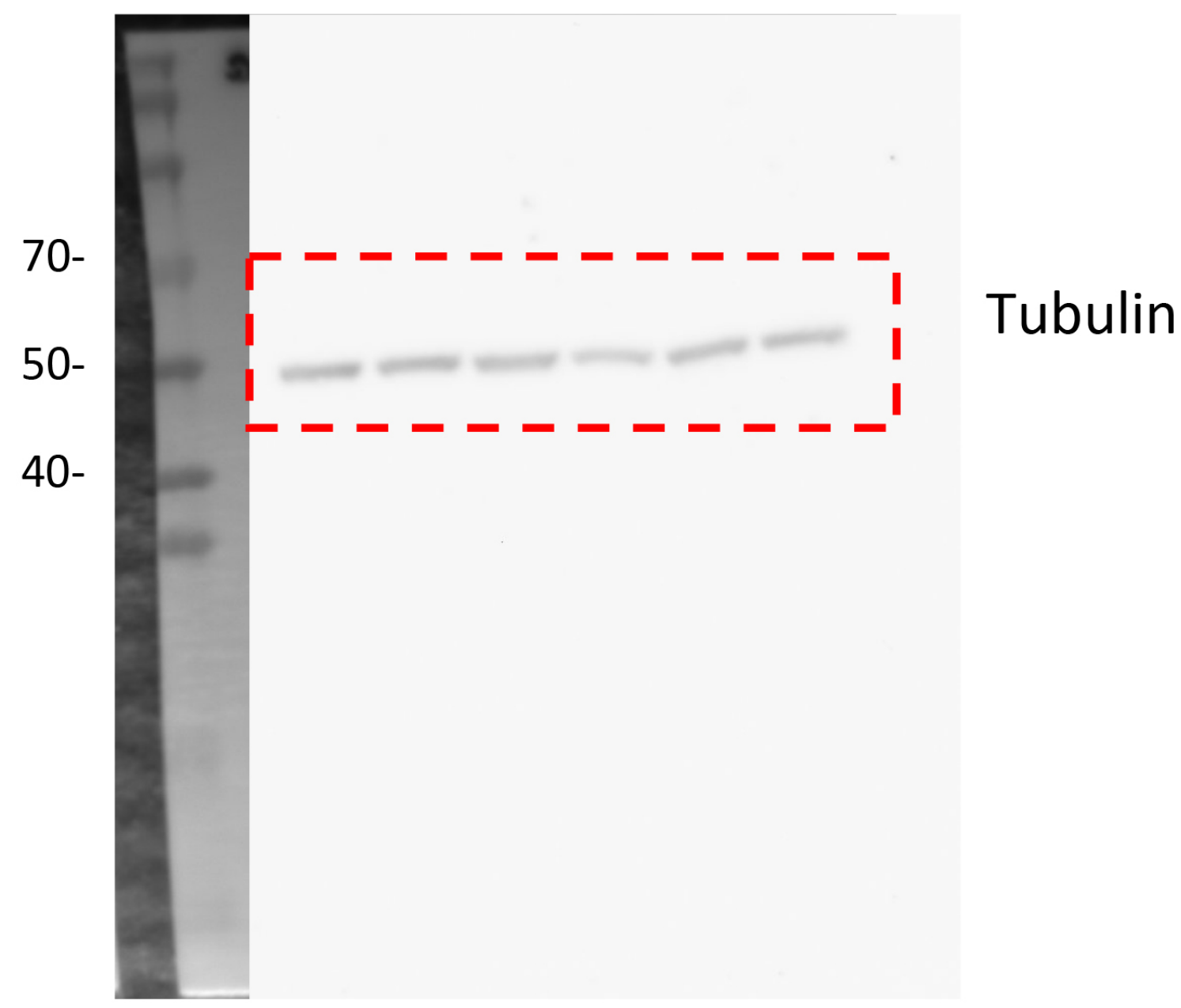

Supplementary Figure 3B

Densitometry triplicates

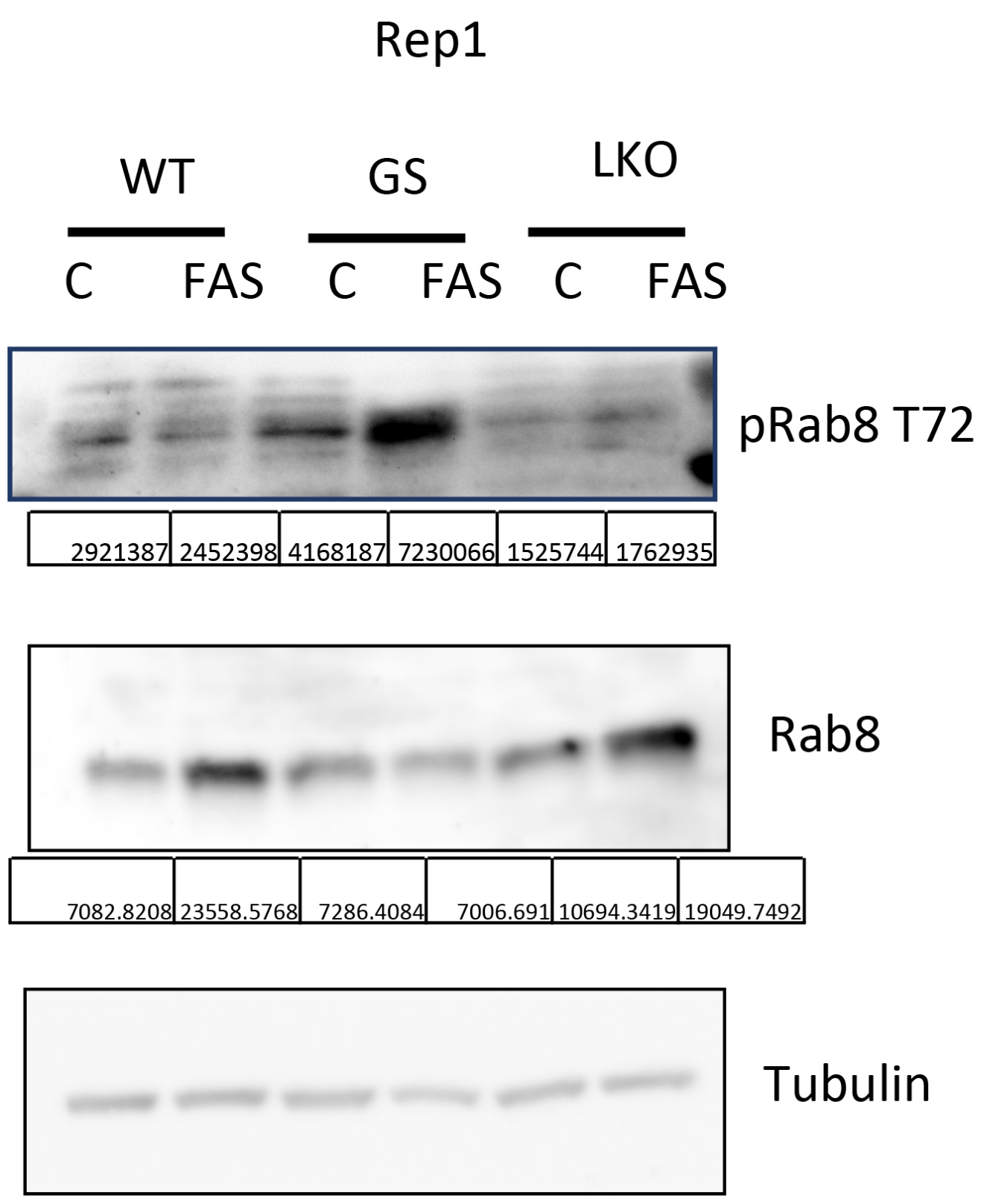

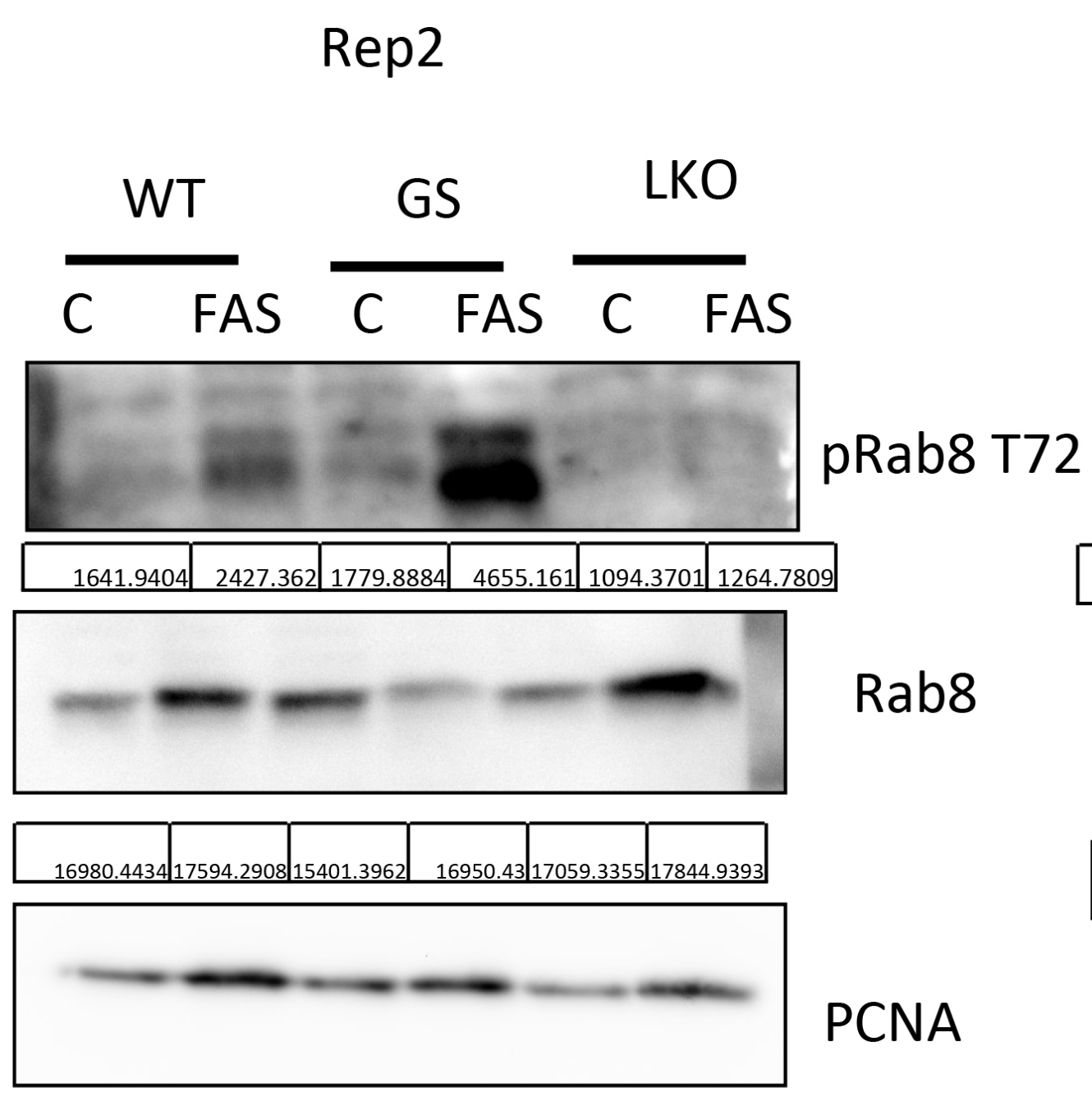

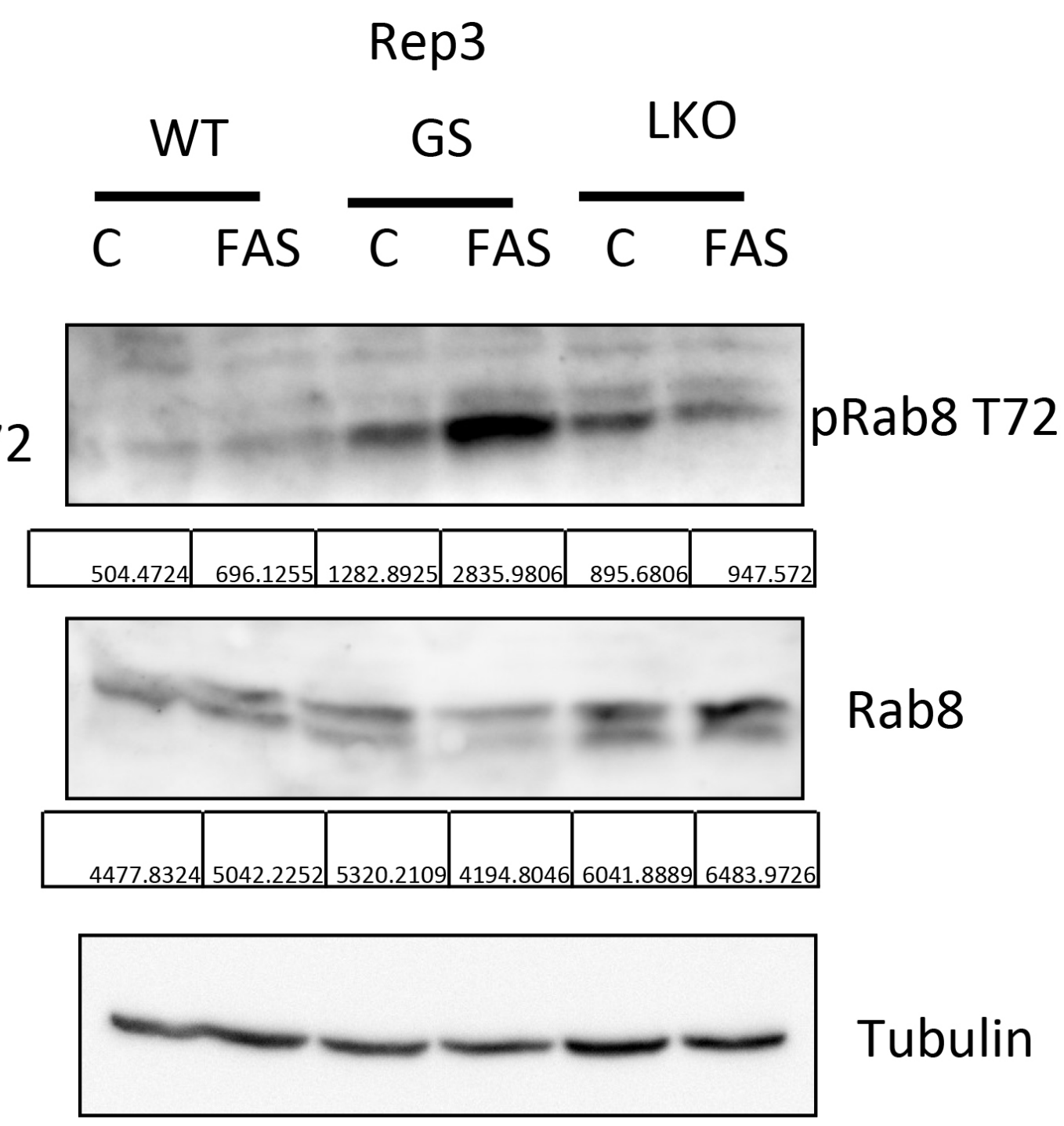

Journal of Cell Science • Supplementary information

Supplementary Figure 3C

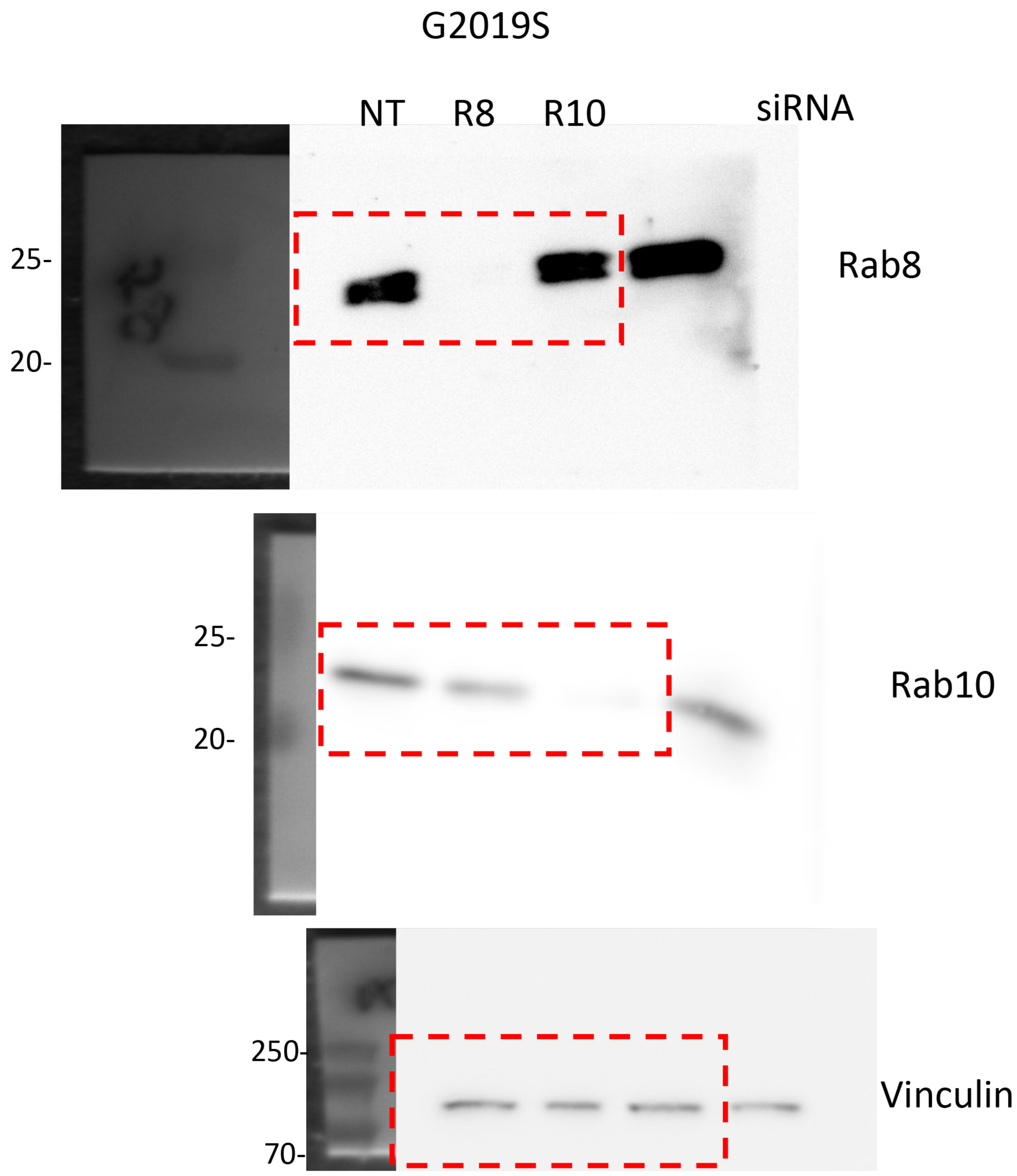

Supplementary Figure 3H

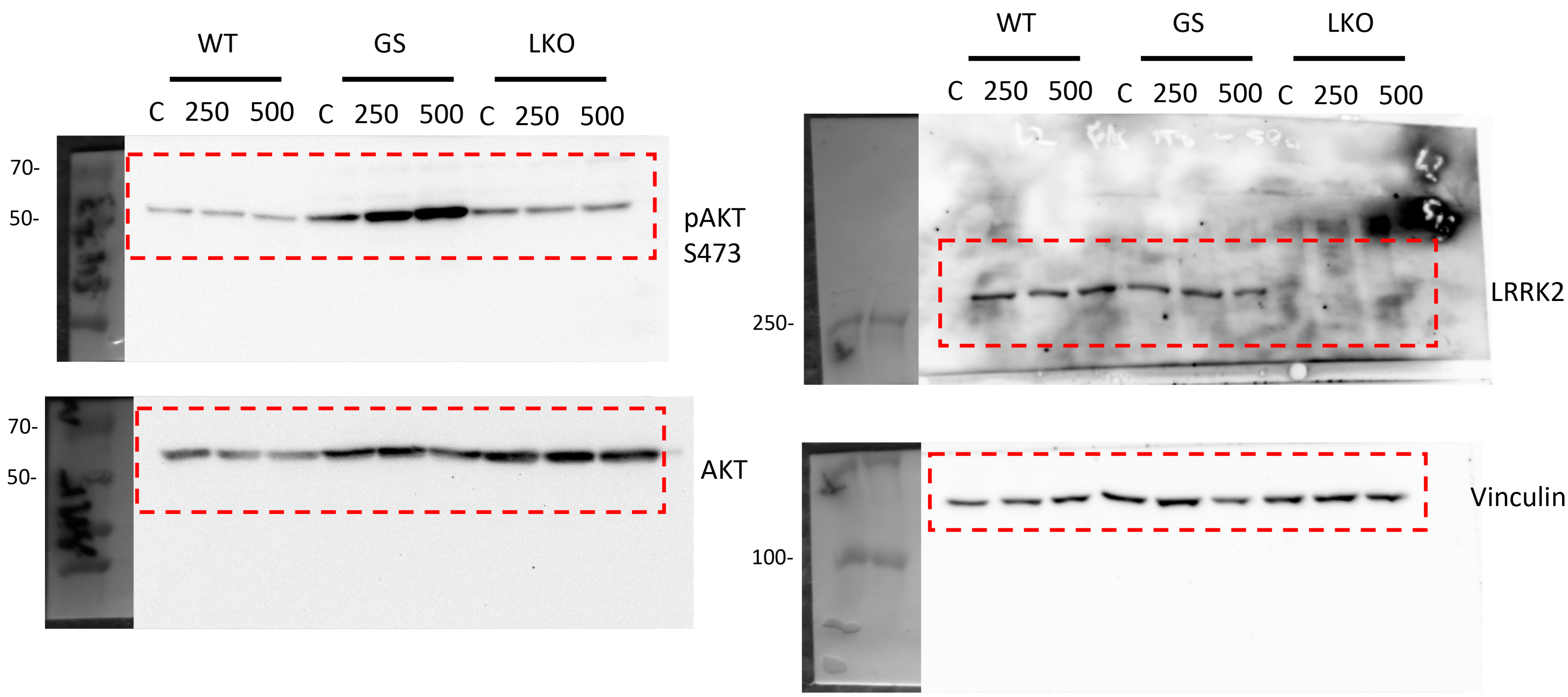

Supplementary Figure 3I

Densitometry triplicates

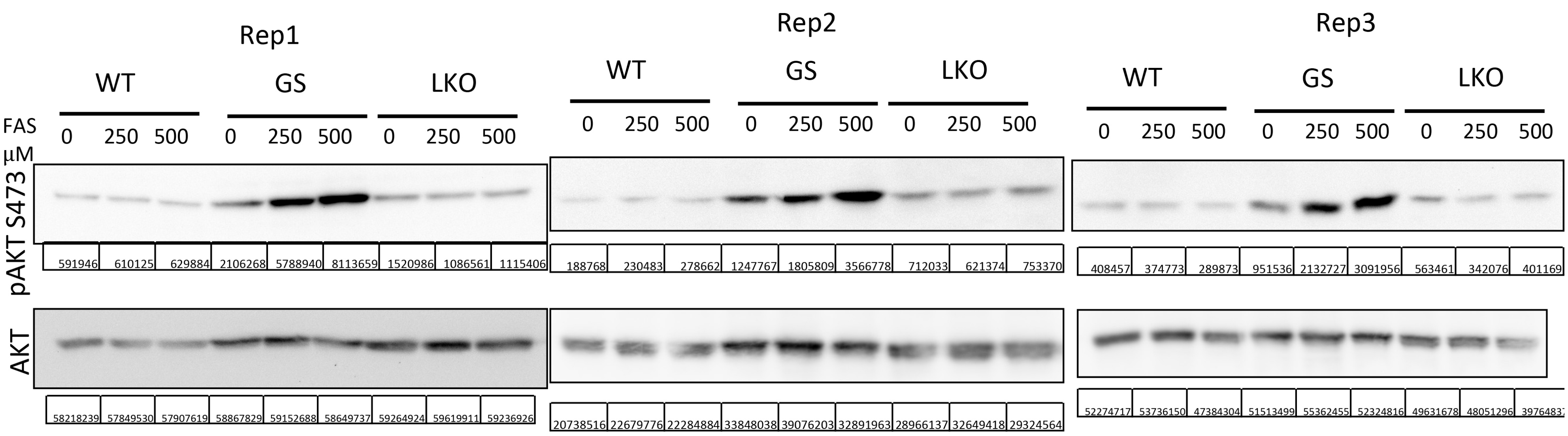

Journal of Cell Science • Supplementary information

Supplementary figure 3J

Densitometry triplicates

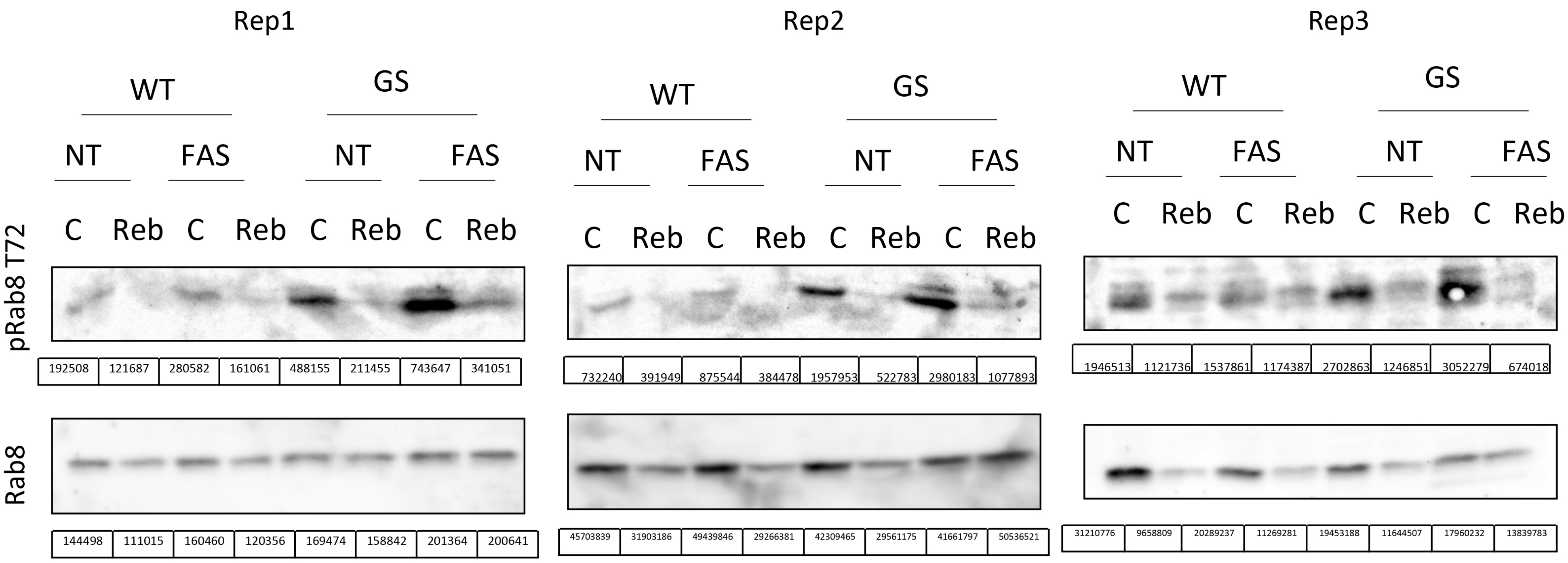

Supplementary Figure 4A

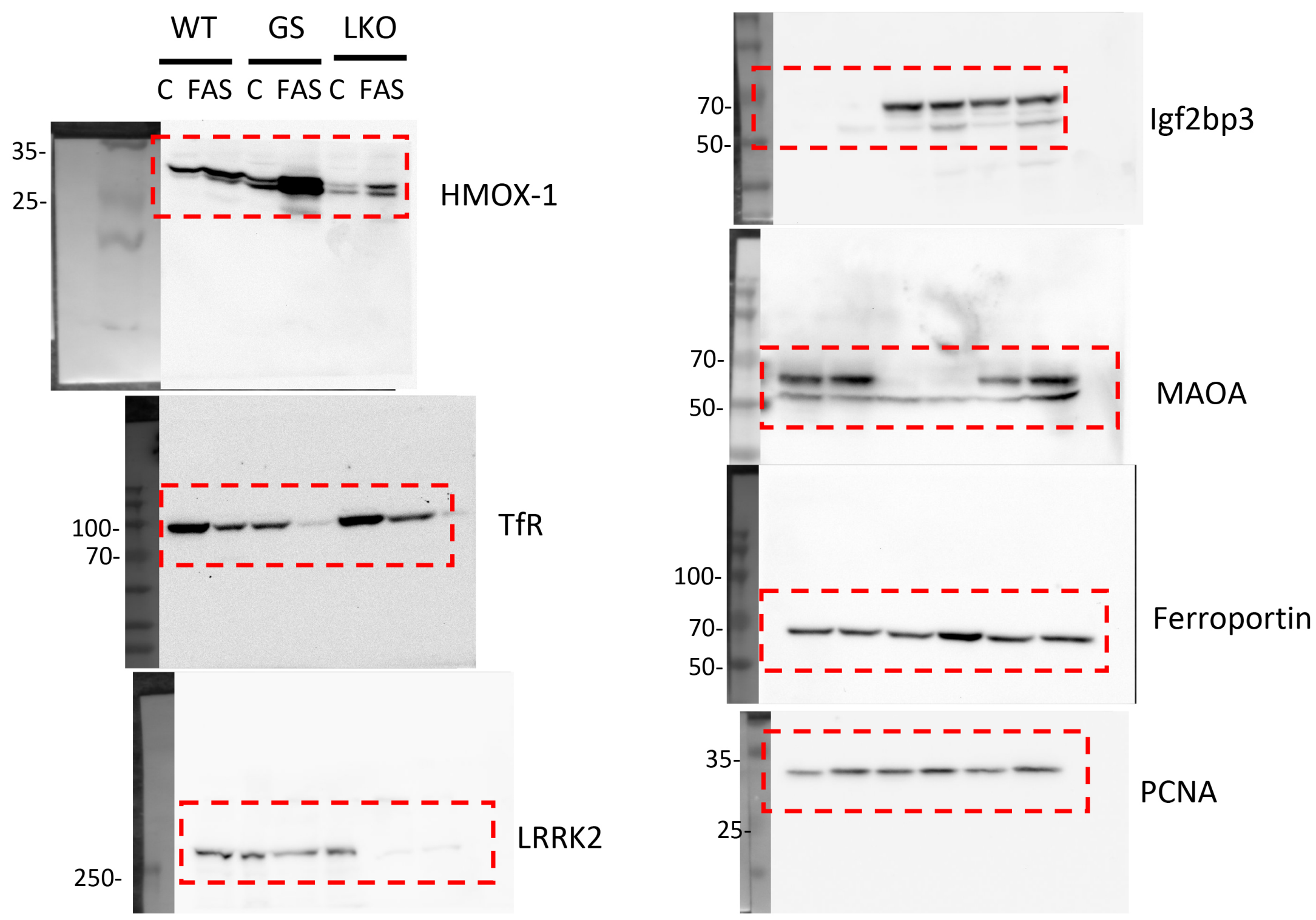

Fig. S5. Blot transparency

Journal of Cell Science • Supplementary information

Table S1. Proteomics dataset from LRRK2G2019S and wild type RAW macrophages.

Available for download at
https://journals.biologists.com/jcs/article-lookup/doi/10.1242/jcs.264977#supplementary-data

Table S2. Proteomics dataset from LRRK2$_{G2019S}$ and wild type RAW macrophages FAS treated macrophages.

Available for download at
https://journals.biologists.com/jcs/article-lookup/doi/10.1242/jcs.264977#supplementary-data

Journal of Cell Science • Supplementary information

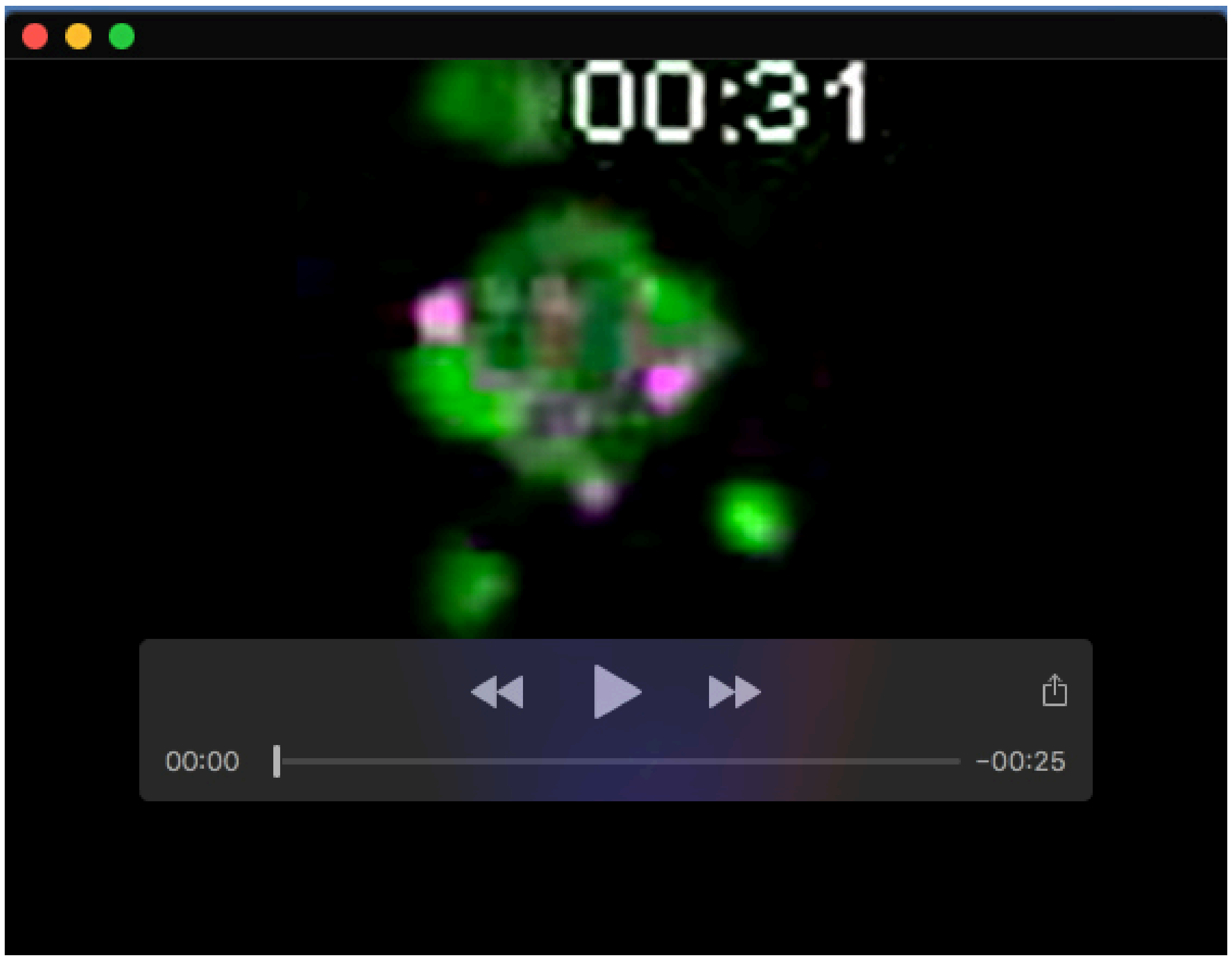

Movie 1. Time-lapse imaging of NCOA4-Cherry foci seen within phase-separated foci entering an enlarged lysosome labeled with Lysotracker Green in wild type cells after 48 hours of 100uM FAS treatment.

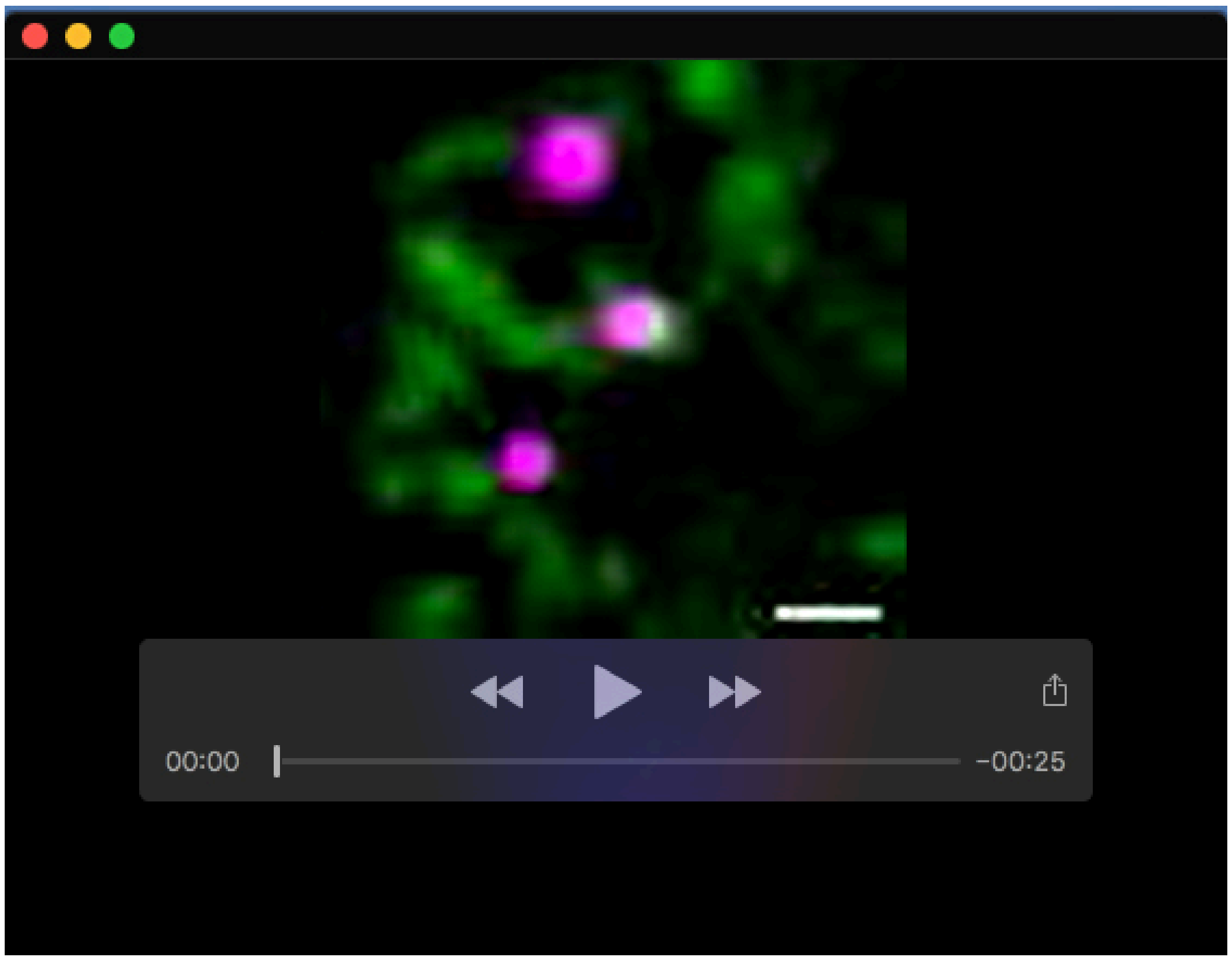

Movie 2. Time-lapse imaging of NCOA4-Cherry enlarged foci that do not become internalized within lysosomes labeled with Lysotracker Green in LRRK2$_{G2019S}$ cells after 48 hours of 100uM FAS treatment.

Journal of Cell Science • Supplementary information
