# Supplementary material for: An LRRK2 variant blocks NCOA4 trafficking upon iron overload, leading to ferroptotic death
Source: J Cell Sci. 2026 Jul 27;139(14):jcs264977. doi: 10.1242/jcs.264977 (PMC13490738; doi:10.1242/jcs.264977)
Supplement: Supplementary file 2 [file joces-139-264977_review_history.pdf]

An LRRK2 variant blocks NCOA4 trafficking upon iron overload, leading to ferroptotic death

Andres Goldman, Mai Nguyen, Joël Lanoix, Chongyang Li, Ahmed Fahmy, Yong Zhong Xu, Erwin Schurr, Pierre Thibault, Michel Desjardins and Heidi M. McBride
DOI: 10.1242/jcs.264977

Editor: Michael Way

Review timeline

Submission to Review Commons:	26 August 2025
Submission to Journal of Cell Science:	21 April 2026
Editorial decision:	27 April 2026
First revision received:	8 May 2026
Accepted:	8 May 2026

Reviewer 1

Evidence, reproducibility and clarity

Goldman et al describe some novel findings with respect to LRRK and iron handling in a series of RAW macrophage cell lines. This cell background is chosen for its recognized high levels of endogenous LRRK2 protein expression, its somewhat broad use in the field, and the investigators add its relevance due to phagocytosis of red blood cells, thus requiring iron robust metabolic processes. Proteomic analyses of WT and G2019S RAW cells revealed multiple iron-related proteins affected by LRRK2 mutation. A deeper candidate-based analysis revealed complex changes in ferritin heavy and light chain and changes in ferric and ferrous iron. Notably, reliable changes in the levels and/or solubility of NCOA4 result from this pathogenic LRRK2 mutation. Unexpectedly, however, these changes were not sensitive to LRRK2 kinase inhibitor treatment. The investigators suggest a dominant effect rather than loss-of-function as subsequent experiments revealed that these effects could be replicated with a LRRK2 variant lacking the kinase domain (LRRK2-ARM) and were not replicated by LRRK2 KO. The data are internally consistent throughout and could certainly shed new important light onto unique and unexpected effects of this LRRK2 mutation.

There are two major concerns with the data in their present form. In brief, first, the G2019S cells express much less LRRK2 and more Rab8 that the WT cells and this severely affects interpretability. Second, the investigators used CRISPR to truncate the endogenous LRRK2 locus to produce a hypothetical truncated LRRK2-ARM polypeptide. This appears to have robust effects on NCOA4, in particular, which drives the overall interpretation of the data.

However, the expression of this novel LRRK2 specie is not confirmed nor compared to WT or G2019S in these cells (although admittedly the investigators did seek to address this with subsequent KO in the ARM cells). It would be premature to account for the changes reported without evidence of protein expression. This latter issue may be more easily addressed and could provide very strong support for a novel function/finding, see more detailed comments below, most seeking clarifications beyond the above.

- Need to make clear in the results whether the G2019S CRISPR mutant is heterozygous or homozygous (presumably homozygous, same for ARM)
- The text of the results implies that MLi2 was used in both WT and G2019S Raw cells, but it's only shown for G2019S. Given the premise for the use of RAW cells, it's important to show that there is basal LRRK2 kinase activity in WT cells to go along with its high protein expression.

© 2026. Published by The Company of Biologists under the terms of the Creative Commons Attribution License (https://creativecommons.org/licenses/by/4.0/).

This is particularly important as the G2019S blot suggests minor LRRK2- independent phosphorylation of Rab8a (and other detected pRabs). One would imagine that pRab8 levels in both WT and G2019S would reduce to the same base line or ratio of total Rab in the presence of MLi2, but WT untreated is similar to G2019S with MLi2. This suggests no basal LRRK2 activity in the Raw cells, but I don't think that is the case.
- Also, in terms of these cells, the levels of LRRK2 are surprisingly unmatched (Fig 1A, 1D, 1H, S1D, etc.) as are total levels of Rab8 (but in opposite directions) between the WT and G2019S. This is not mentioned in the Results text and is clearly reproducible and significant.

Why do the investigators think this is? If Rab8 plays a role in iron, how do these differences affect the interpretation of the G2019S cells (especially given that MLi2 does not rescue)? Are other LRRK2-related Rabs affected at the protein (not phosphorylation level)? Could reduced levels of LRRK2 or increase Rab 8 alone or together account for some of these differences? Substantial further characterization is required as this seriously affects the interpretability of the data. Since pRab8 is not normalized to total Rab8, this G2019S model may not reflect a total increase in LRRK2 kinase activity, and could in fact have both less LRRK2 protein and less cellular kinase activity than WT (in this case).
- Presumably, the blots in 1H are whole cell lysates and account for the pooled soluble and insoluble NCOA4 (increased in G2019S), as there is no difference in soluble NCOA4 (Fig 2H). I suspect the prior difference is nicely reflected in the insoluble fraction (Fig 2H). This should be better explained in the Results text. This is a very interesting finding and I wonder what the investigators believe is driving this phenotype? Is the NCOA4 partitioning into a detergent-inaccessible compartment? Does this replicate with other detergents, those perhaps better at solubilizing lipid rafts? Is this a phenotype reversible with MLi2? Very interesting data.
- Figure 2 describes the increased NCOA4-positive iron structures after iron load, but does not emphasize that the G2019S cells begin preloaded with more NCOA4. How do the investigators account for differential NCOA4 in this interpretation? Is this simply a reflection of more NCOA4 available in G2019S cells? This seems reasonable.
- These are very long exposures to iron, some as high as 48 hr which will then take into account novel transcriptomic and protein changes. Did the investigators evaluate cell death? Iron uptake would be trackable much quicker.
- The legend for 2F is awkward (BSADQRED)
- Why are WT cells not included in Fig 2G?
- The biochemical characterization of NCOA4 in the LRRK2-arm cells is a great experiment and strength of the paper. The field would benefit by a bit further interrogation, other detergents, etc.
- Have the investigators looked for aberrant Rab trafficking to lysosomes in the LRRK2-arm cells? Is pRab8 mislocalized compared to WT? Other pRabs?
- The expression levels and therefore stability of the ARM fragment is not shown. This is necessary for interpretation. While very intriguing, the data in Aim 3 rely on the assumption that the ARM fragment is expressed, and at comparable levels to G2019S to account for phenotypes. The generation of second clone is admirable, but the expression of the protein must be characterized. This is especially true because of the different LRRK2 levels between WT and G2019S. One could easily conceive of exogenous expression of a tagged-ARM fragment into LRRK2 KO cells, for example, as another proof-of-concept experiment. If it is truly dominant, does this effect require or benefit from some FL LRRK2? It seems easy enough to express the LRRK2-ARM in at least WT and KO RAW cells.
- Does iron overload induce Rab8a phosphorylation in a LRRK2 KO cell? This would be a solid extension on the ARM data and support the important finding that an additional kinase(s) can phosphorylate Rab8a under these conditions, and while not unexpected, this may not have been demonstrated by others as clearly. It also addresses whether the ARM domain is important to this other putative kinase(s), which may add value to the authors' model.

Minor concern - the abstract but not the introduction emphasizes a hypothesis that loss of neuromelanin may promote cell loss in PD (through loss of iron chelation), while post mortem studies are by definition only correlative, early works suggested that the higher melanized DA neurons were preferentially lost when compared to poorly melanized neurons in PD. This speculation in the abstract is not necessary to the novel findings of the paper.

Significance

© 2026. Published by The Company of Biologists under the terms of the Creative Commons Attribution License (https://creativecommons.org/licenses/by/4.0/).

This study could shed light on a both novel and unexpected behavior of the LRRK2 protein, and open new insights into how pathogenic mutations may affect the cell. While studied in one cell line known for unusually high LRRK2 expression levels, data in this cell type have been broadly applicable elsewhere. Give the link to Parkinson's disease, Rab-dependent trafficking, and iron homeostasis, the findings could have import and relevance to a rather broad audience.

Reviewer 2

Evidence, reproducibility and clarity

In this manuscript the authors describe an interesting connection between the Parkinson's kinase LRRK2 and iron trafficking in RAW macrophages. Expression of the LRRK2 G2029S mutation affects the abundance of ferritin heavy and light chains and therefore the uptake and storage of iron. Interestingly, the loss of the kinase domain still had a strong phenotype, suggesting that this is independent of the kinase function.

The paper is well written and excellently cited. The data is convincing and of good quality.

I have only one request and else very minor comments:

Major: Please confirm that the observed phenotype is conserved within bone marrow-derived macrophages of LRRK2 G2019S mice. These mice are widely available within the community and frozen bone marrow could be sent to the labs.

The main reason for this experiment is that CRISPR macrophage cell lines do sometimes acquire weird phenotypes (at least in our lab they sometimes do!) and it would strengthen the validity of the observations.

Minor comments:

Supplementary Fig 1: I don't think one should normalize all controls to 1 and then do a statistical test as obviously the standard deviation of control is 0. I would normalize to the average of the control, which will provide an error for the control.

The raw data needs to be submitted to PRIDE or similar. This has not happened yet.

Some of the western blots could be improved. If these are the best shown, I am a little concerned about the reproducibility. How often has they been done?

Significance

Considering the importance of LRRK2 biology in Parkinson's and the new biology shown, this paper will be of great interest to the community and wider research fields.

Reviewer 3

Evidence, reproducibility and clarity

In this paper, the authors report an interesting phenotype of the LRRK2 G2019S mutation on iron homeostasis in RAW264.7 macrophages. The phenotype is well characterised through proteomic and western blot approaches investigating transferrin and ferritin trafficking. The study is well conducted and data of high quality. The authors also appear to have discovered a cellular context where Rab8 is phosphorylated independently of LRRK2. This is a major finding which can potentially have an important impact in the LRRK2 field.
What is missing in the study is the physiological relevance of these findings, mainly whether this effect actually results in higher cell death during iron overload. Since iron overload is known to result in ferroptosis, it is surprising that the authors have not checked whether the

© 2026. Published by The Company of Biologists under the terms of the Creative Commons Attribution License (https://creativecommons.org/licenses/by/4.0/).

LRRK2 G2019S and ARM cells undergo more ferroptosis relative to LRRK2 WT cells. Moreover, their conclusion of the findings as "resistant to LRRK2 kinase inhibitors" is not convincing, since in most of the studies, they have removed the kinase domain, and this description implies the use of pharmacological kinase inhibition which has not been done in this paper.

Significance

Major comments
In Figure 1:
- There is lower LRRK2 expression in LRRK2 G2019S cells, have the authors checked Rab phosphorylation to validate the mutation?
- The authors should specify if their cells are heterozygous or homozygous since they are discussing a dominant interfering mutant.
- The transferrin phenotype validated through proteomics and western blot is solid.
- Quantification in figure 1F-G is problematic, not clear what they mean by "diffuse and lysosomal". Puncta is either colocalising with lysosomes or not colocalising. This needs to be clarified and re-analysed.
- Text in the first results part called "LRRK2G2019S RAW macrophages have altered iron homeostasis" is very long. It could be divided into more sections to improve readability.

In Figure 2:

If the effect is armadillo-dependent, where does LRRK2 G2019S is implicated since there is no kinase domain in these cells?
- The authors do not show any controls (PCR, sequencing) confirming knockout or truncation.
- The data is interesting and the image quality with the insets is very high. In Figure 3:
- Mutant not clearly described in text, did the authors remove just the kinase and ROC-COR domains or all the domains downstream of the Armadillo domain? This is not clear.
- The authors cannot conclude that their phenotype is due to the independence of the kinase domain specifically as they are also interfering with the GTPase activity by removing the ROC-COR domains.
- In Figure 3E, is the difference between the "ARM CTRL" and the "ARM FAS" conditions significant? A trend appears to be there, but the p-value is not shown.

In Figure 4:

- In figure 4A, it would have been important to check if Rab8 phosphorylation is also observed in LRRK2 KO cells after administration of FAS to further evaluate the mechanism through which this Rab8 phosphorylation is occurring.
- The vinculin bands in figure 4A are misaligned with the rest of the bands.
- The authors do not have any controls to validate the pRab8 staining in IF. This is an important caveat and needs to be addressed.
- The authors should have checked if FAS administration in the LRRK2 G2019S and the ARM cells is leading to ferroptotic cell death (or cell death in general). This is key to validate the link between the altered iron homeostasis in LRRK2 G2019S cells and increased cytotoxicity observed during neurodegeneration.
- Regarding the literature, the authors are missing some important papers that are preprinted and these studies need to be discussed. This includes a report with opposite findings https://www.biorxiv.org/content/10.1101/2025.09.26.678370v1.full and a report showing kinase independent cell death in macrophages https://www.biorxiv.org/content/10.1101/2023.09.27.559807v1.abstract

---

Author response to reviewers' comments

© 2026. Published by The Company of Biologists under the terms of the Creative Commons Attribution License (https://creativecommons.org/licenses/by/4.0/).

Goldman et al., Response to Review April 16, 2026

Referee 1 (R1): The revised manuscript by Goldman et al has changed significantly since its first submission. Upon further analysis of a putatively truncated mutant, the authors have removed the interpretation that G2019S LRRK2 alters iron handling through a kinase-independent mechanism. They should be commended for the transparency and rigor applied in this revision and the resulting data are conceptually easier to digest.

Heidi McBride (HM): We thank the reviewer for their understanding. We would point out that our phenotypes remain resistant to the commonly used LRRK2 kinase inhibitor MLi2, yet are sensitive to the type II kinase inhibitors which we believe is novel.

R1: However, now that the proposed pathway involves over-active LRRK2 kinase activity and a putative role for Rab8a phosphorylation, the manuscript is rather descriptive in nature. The mechanisms underlying dramatic changes in NCOA4, FTL, and FTH protein levels upon iron exposure are not explored. In addition, the reliance on these effects, or the precise involvement of LRRK2 substrates is not established.

HM: We agree there remains much to be uncovered mechanistically. However, the fact that phosphorylated Rab8 is active at the plasma membrane upon iron overload is novel, suggesting more broad roles beyond the Golgi/lysosomal localization of LRRK2 substrates. We would also argue that the majority of studies on LRRK2 follow the phosphorylation of Rab substrates by western blot, but the functional roles of these Rabs, their GTPase cycles and effectors, in membrane traffic is not at all clear within the field. It is difficult for us to determine the mechanisms by which NCOA4, FTL and FTH are altered, but we did expand our work in the revision to include full proteomics analysis of control and iron treated cells with wild type or LRRK2G2019S to get a better picture of the changes that occur. This included major changes in lysosomal hydrolyases, induction of oxidative stress pathways linked to ferroptotic cell death, and cytoskeletal changes that may link to the plasma membrane blebbing we observe. Our data indicate that the hyperactivity of LRRK2 during iron overload is having significant impact on the capacity of the lysosome to handle the iron, which we observe also at the morphological level in the loss of microautophagy of NCOA4. Taken together, the impact of our work lies in setting a new framework by which to examine LRRK2 function in iron homeostasis.

R1: The limitation of a single LRRK2 mutant (in the context of a more canonical increased kinase activity mechanism) is also more evident. G2019S is a relatively weak Rab kinase when compared to Y1699C or R1441C - is there a possibility for G2019S In addition, the impact of this iron defect is limited to oxidative cell injury and implications for ferroptosis in macrophages (not neurons, where these outcomes might be of greater relevance) whereas how these phenotypes affect the immune-like functions of the chosen cell type (e.g. cytokine release, chemotaxis, phagocytosis) is not addressed.

HM: We agree that the examination of additional LRRK2 mutations in our model would be important, but the G2019S is the most common patient mutation and has a very strong impact on the cellular response to iron. As for the impact on cytokine release, chemotaxis and phagocytosis was not requested in the first round of review and we believe is beyond the scope of our current study.

R1: While the proteomics and other analyses provide strong rigor for the data that are presented, without more mechanistic interrogation or consideration of cell type specific behaviors, the manuscript does not advance very far beyond confirming that iron related proteins altered in parallel with iron itself and highly dependent on iron loading to evoke phenotypes.

HM: We thank the reviewer for recognizing the rigour of our revisions. We did interrogate the function of the oxidative stress signatures and performed experiments to demonstrate the oxidation of lipids in the lysosome along with assays for ferroptotic cell death. We highlighted key signatures that were changed, including the selective loss of lysosomal enzymes, and changes in cellular cytoskeletal elements. We agree that there is more to do, but our data has provided many important new observations on what exactly is changed in LRRK2G2019S cells in response to iron, from lysosomal morphology changes, to critical analysis of NCOA4 and ferritin dynamics, the novel

© 2026. Published by The Company of Biologists under the terms of the Creative Commons Attribution License (https://creativecommons.org/licenses/by/4.0/).

observation of phosphorylated Rab8 at the cell surface, and a very unexpected insensitivity to MLi2 yet selective inhibition by new type II LRRK2 kinase inhibitors. With this we believe our work does provide a great deal of novelty and impact on LRRK2 functions in iron homeostasis.
A final point on the concept that the phenotypes are only induced upon iron overload. We would point out that we observed changes in ferritin and NCOA4

Original submission

First decision letter

MS ID#: jcs.264977

MS TITLE: LRRK2G2019S interferes with NCOA4 trafficking in response to iron overload leading to oxidative stress and ferroptotic cell death.

AUTHORS: Andres Goldman; Mai Nguyen; Joël Lanoix; Ahmed Fahmy; Yong Zhong Xu; Erwin Schurr; Pierre Thibault; Michel Desjardins; Heidi McBride; Chongyang Li
ARTICLE TYPE: Review Commons Transfer

Thank you for forwarding your paper from review commons. Based on your response to reviewers and full revision, I am prepared to accept your paper. However, I feel that the addition of a limitations section at the end of the discussion would benefit the manuscript and help the readers.

Author response to reviewers' comments

We were thrilled to receive the positive response from JCS to our submitted manuscript entitled “LRRK2 mutations block NCOA4 trafficking upon iron overload leading to ferroptotic death”. We have taken the time to add a new heading within the discussion related to the limitations of our study, as requested. We also made a few small edits and corrections within the methods as we made final checks and updated the zenodo files. I am including the response to reviews (below) from the Review Commons system that we included in the initial submission to JCS.

This work was funded through an organization (ASAP) that has a rather complex and formal system to check and confirm the manuscript follows their rules for open science and AI accessibility to all raw data, reagents and files. This was all sent to them for approval some time ago and we are confident that will come in the next few days. But the zenodo links are all live now and we hope we have done everything in accordance with your guidelines.

We are sincerely grateful for the opportunity to publish our work in JCS, one of the foundational journals in Cell Biology for which I’ve always held in very high esteem!

We look forward to hearing the final word if this study is now acceptable for publication and will work rapidly to get to the final edits/proofs with your team.

Referee 1 (R1): The revised manuscript by Goldman et al has changed significantly since its first submission. Upon further analysis of a putatively truncated mutant, the authors have removed the interpretation that G2019S LRRK2 alters iron handling through a kinase-independent mechanism. They should be commended for the transparency and rigor applied in this revision and the resulting data are conceptually easier to digest.

Heidi McBride (HM): We thank the reviewer for their understanding. We would point out that our phenotypes remain resistant to the commonly used LRRK2 kinase inhibitor MLi2, yet are sensitive to the type II kinase inhibitors which we believe is novel.

© 2026. Published by The Company of Biologists under the terms of the Creative Commons Attribution License (https://creativecommons.org/licenses/by/4.0/).

R1: However, now that the proposed pathway involves over-active LRRK2 kinase activity and a putative role for Rab8a phosphorylation, the manuscript is rather descriptive in nature. The mechanisms underlying dramatic changes in NCOA4, FTL, and FTH protein levels upon iron exposure are not explored. In addition, the reliance on these effects, or the precise involvement of LRRK2 substrates is not established.

HM: We agree there remains much to be uncovered mechanistically. However, the fact that phosphorylated Rab8 is active at the plasma membrane upon iron overload is novel, suggesting more broad roles beyond the Golgi/lysosomal localization of LRRK2 substrates. We would also argue that the majority of studies on LRRK2 follow the phosphorylation of Rab substrates by western blot, but the functional roles of these Rabs, their GTPase cycles and effectors, in membrane traffic is not at all clear within the field. It is difficult for us to determine the mechanisms by which NCOA4, FTL and FTH are altered, but we did expand our work in the revision to include full proteomics analysis of control and iron treated cells with wild type or LRRK2G2019S to get a better picture of the changes that occur. This included major changes in lysosomal hydrolyases, induction of oxidative stress pathways linked to ferroptotic cell death, and cytoskeletal changes that may link to the plasma membrane blebbing we observe. Our data indicate that the hyperactivity of LRRK2 during iron overload is having significant impact on the capacity of the lysosome to handle the iron, which we observe also at the morphological level in the loss of microautophagy of NCOA4. Taken together, the impact of our work lies in setting a new framework by which to examine LRRK2 function in iron homeostasis.

R1: The limitation of a single LRRK2 mutant (in the context of a more canonical increased kinase activity mechanism) is also more evident. G2019S is a relatively weak Rab kinase when compared to Y1699C or R1441C - is there a possibility for G2019S In addition, the impact of this iron defect is limited to oxidative cell injury and implications for ferroptosis in macrophages (not neurons, where these outcomes might be of greater relevance) whereas how these phenotypes affect the immune-like functions of the chosen cell type (e.g. cytokine release, chemotaxis, phagocytosis) is not addressed.

HM: We agree that the examination of additional LRRK2 mutations in our model would be important, but the G2019S is the most common patient mutation and has a very strong impact on the cellular response to iron. As for the impact on cytokine release, chemotaxis and phagocytosis was not requested in the first round of review and we believe is beyond the scope of our current study.

R1: While the proteomics and other analyses provide strong rigor for the data that are presented, without more mechanistic interrogation or consideration of cell type specific behaviors, the manuscript does not advance very far beyond confirming that iron related proteins altered in parallel with iron itself and highly dependent on iron loading to evoke phenotypes.

HM: We thank the reviewer for recognizing the rigour of our revisions. We did interrogate the function of the oxidative stress signatures and performed experiments to demonstrate the oxidation of lipids in the lysosome along with assays for ferroptotic cell death. We highlighted key signatures that were changed, including the selective loss of lysosomal enzymes, and changes in cellular cytoskeletal elements. We agree that there is more to do, but our data has provided many important new observations on what exactly is changed in LRRK2G2019S cells in response to iron, from lysosomal morphology changes, to critical analysis of NCOA4 and ferritin dynamics, the novel observation of phosphorylated Rab8 at the cell surface, and a critical insensitivity to MLi2 with selective inhibition by new type II LRRK2 kinase inhibitors. With this we believe our work does provide a great deal of novelty and impact on LRRK2 functions in iron homeostasis.

A final point on the concept that the phenotypes are only induced upon iron overload. We would point out that we observed changes in ferritin and NCOA4 levels in LRRK2G2019S cells even without iron overload, so there are indeed impact on lysosomal and iron homeostasis in steady state. It is also perhaps important to note that LRRK2 is established to be activated in response to cellular/lysosomal stressors, so it is not unusual to observe the impact of the mutations only in certain conditions. This would be consistent with the mutations being linked to an age-related disease with relatively low penetrance among heterozygous carriers. We would argue that it is

© 2026. Published by The Company of Biologists under the terms of the Creative Commons Attribution License (https://creativecommons.org/licenses/by/4.0/).

important to understand exactly which types of cellular stressors may lead to pathology, and that iron dysregulation has been a long-standing candidate stressor in PD.

****

Referee 2 (R2): The authors have done all I have requested before.

HM: We thank the reviewer for acknowledging our efforts in addressing each of the reviewers concerns.

R2: I am concerned about the substantial changes to the original manuscript including the realisation that some clones were not the knock-outs expected. However, removing these, is the right thing.

HM: We agree that the removal of the truncated clone, along with our identification of a class of kinase inhibitors that reversed the p-Rab8 upon iron overload led to changes in a key conclusion of the first submission; namely that the effects were kinase-independent. In our opinion, this does not change the fundamental observations we have made on the impact of LRRK2 on iron homeostasis, many of which are made here for the first time.

R2: I still have major concerns that the phenotype could not be reproduced in BMDMs. The RAW cells are a good tool, but far from physiological. The authors' arguments that others have also seen a phenotype in iron homeostasis is however reassuring.

HM: We tried to make it very clear throughout our manuscript that there is a cell specificity to the iron phenotypes observed in LRRK2G2019 backgrounds. As stated in our response to review, we did observe changes in NCOA4 within BMDM but not the levels of FTL and FTH. We will continue to explore the impact of LRRK2 mutations in primary lines in future work. However, this should not negate the phenotypes we observe in the RAW cells that are consistent with other observations from ssRNAseq, and the work of others looking at microglia upon LPS treatment in vivo, as an example.

R2: I leave it to the editor if they feel that a somewhat artificial cell line is the right model.

HM: The RAW macrophages are not “artificial”. Widely used in Parkinson’s research, cancer and immunity, the ATCC site describes “RAW 264.7 are an adherent cell line isolated from a mouse tumor that was induced by Abelson murine leukemia virus. This cell line, with macrophage differentiation, can be used in oxidative stress, inflammatory, and antibacterial activity studies.” We understand that the standard of cell culture is rapidly moving entirely to human iPSC derived lines and organoids. We argue that there is still a role for fundamental cell biological studies to be done in established cultured cell lines where we can more rapidly generate critical hypothesis to be tested in other model systems in future studies. There has been no published work linking LRRK2 to ferritinophagy and NCOA4 microautophagy before this, and we humbly submit that we have made some critical new observations in our study that will direct future work in a highly targeted manner.

****

Referee 3 (R3): We thank the authors for addressing our concerns. As a result, the work looks very different, and the main message has changed.

HM: We thank the reviewer for acknowledging our efforts in the revision. We agree that one point of our study, that the effects of the LRRK2G2019S were kinase independent, has changed. However, the rest of our work stands and was extended in the revision with many new experiments highlighting the impact of LRRK2G2019S on iron homeostasis. In addition, our experiments still confirmed that the main kinase inhibitor used across all studies on LRRK2 did not block the phenotypes we observed. In revision we sought out additional classes of LRRK2 kinase inhibitors and then demonstrated that these phenotypes were sensitive to inhibition by Type II kinase inhibitors. Therefore the fact that LRRK2 linked iron phenotypes are not blocked by the common kinase inhibitor remains unchanged.

© 2026. Published by The Company of Biologists under the terms of the Creative Commons Attribution License (https://creativecommons.org/licenses/by/4.0/).

R3: The main issue with this new manuscript relative to the previous one is that a key part of the novelty has been lost. In the original draft the authors claimed to have found a LRRK2 kinase-independent phosphorylation of Rab8. This was a major new finding potentially hinting that LRRK2 is not the only kinase for Rab8 which would have had major ramifications for the field. All data from this truncation mutant has been withdrawn from the manuscript and the message is now significantly different. It is unfortunate that the armadillo domain data from the original manuscript has been withdrawn due to the results of the proteomics experiment revealing 7 peptides C-terminal to their stop codon. I suggest that, independently of the fate of the current manuscript, the authors update the biorxiv preprint as in the current manuscript this is not addressed (as this part has been removed) and the community needs to be aware of the changes that are potentially misleading.

HM: While the new data show that p-Rab8 can be inhibited by type II kinase inhibitors upon iron overload, we present a great deal of novelty showing that this activated Rab8 is at the plasma membrane upon iron loading in G2019S cells. We also performed experiments examining ferritinophagy and NCOA4 microautophagy in these conditions, which we argue are the first instance of these pathways being impacted by LRRK2. Our full proteomics datasets in Figure 4 provide an unbiased view of all changes driven by LRRK2G2019S that led us to additional functional analysis of lipid oxidation and ferroptosis pathways being impacted. Again, we agree that the concept that our phenotypes are no longer kinase independent, however this should not negate the many important observations we have made in our study.

We apologize for not updating the manscript on Biorxiv. I had linked our response to reviews to the bioRxiv during submission to EMBO J and thought the manuscript would automatically be updated. So it was an oversight on my part, which has now been remedied, including all links to raw data files and protocols linked to Zenodo.

R3: Despite the changes and modifications, the main concern remains. The authors are claiming in the title to have discovered that the LRRK2 G2019S mutation leads to more ferroptotic cell death. However, the only cell death data that is shown is a Sytox experiment and a C11-Bodipy stain. Although this is informative about whether cell death is occurring, the authors need more evidence that this is being caused by ferroptosis. For example, they could have probed for GPX4 levels in their lysates as the levels would decrease in ferroptosis. Including inducers and inhibitors of ferroptosis (erastin and ferrostatin-1) would confirm if the FAS-induced LRRK2 G2019S cell death phenotype is mediated by ferroptosis.

HM: We appreciate that the impact of LRRK2G2019S on lipid oxidation and cell death were new to the revision and could have been explored further. However, as we were under a time constraint for the revisions we didn’t employ erastin and ferrostatin-1 in our study. The decision to look at ferroptosis came from the new proteomics analysis we did on wild type and G2019S cells in absence or presence of iron overload. This led us to uncover a major oxidation signature consistent with a sensitivity to ferroptosis. Therefore we used two common assays to test this, that was included in our new Figure 4. Ferroptosis is driven by oxidized lipids, which we observe in lysosomes using the C-11 Bodipy stains, and a rupture of the plasma membrane that can be monitored by the uptake of SYTOX green. SYTOX green uptake also occurs in pyroptotic and necrotic cell death, but the combination of oxidized lipids with SYTOX green is very commonly used to conclude the death is ferroptotic. In our case, we are also adding very high levels of iron to the cells, which would be further suggestive of ferroptosis.

Our proteomics analysis does not show any changes in GPX4, but again, we are in conditions of high iron overload, which is very different from experiments with erastin that inhibit this channel to drive ferroptosis. The accumulation of iron in the late endosome, where we see the accumulation of oxidized C-11 Bodipy is consistent with several recent studies all demonstrating critical roles for lysosomal iron in the regulation of ferroptosis (see new review in Trends in Cell Biology, March 30 2026 DOI: 10.1016/j.tcb.2026.03.007). The bioRxiv paper looking at LRRK2G2019S and other mutants also showed increased oxidized lipids and suggested a susceptibility to ferroptosis (the paper R3 wanted us to cite during first review). Therefore, we agree that there will be much more to learn about how exactly LRRK2 may govern the lysosomal/cellular oxidation pathways, we consider our use of proteomics, lipid probes and cell death assays to be sufficient to

© 2026. Published by The Company of Biologists under the terms of the Creative Commons Attribution License (https://creativecommons.org/licenses/by/4.0/).

conclude that LRRK2G2019 renders our macrophages sensitive to ferroptotic cell death upon iron overload.

Second decision letter

MS ID#: jcs.264977R1

MS Title: LRRK2 mutations block NCOA4 trafficking upon iron overload leading to ferroptotic death

Authors: Andres Goldman; Mai Nguyen; Joël Lanoix; Ahmed Fahmy; Yong Zhong Xu; Erwin Schurr; Pierre Thibault; Michel Desjardins; Heidi McBride; Chongyang Li
Article Type: Review Commons Transfer

Dear Heidi,

I am happy to tell you that your manuscript has been accepted for publication in Journal of Cell Science, pending standard publication integrity checks. It was accepted on 08 May 2026.

Thank you for sending your manuscript to Journal of Cell Science through Review Commons.

© 2026. Published by The Company of Biologists under the terms of the Creative Commons Attribution License (https://creativecommons.org/licenses/by/4.0/).
